# Gene regulatory networks in lactation: identification of global principles using bioinformatics

**DOI:** 10.1186/1752-0509-1-56

**Published:** 2007-11-27

**Authors:** Danielle G Lemay, Margaret C Neville, Michael C Rudolph, Katherine S Pollard, J Bruce German

**Affiliations:** 1 Department of Food Science and Technology, University of California, One Shields Ave., Davis, CA 95616, USA; 2 Department of Physiology and Biophysics, University of Colorado Health Sciences Center, 12800 E. 19th Ave., Room 5103, P.O. Box 6511, Aurora, CO 80045, USA; 3 Department of Statistics, University of California, One Shields Ave., Davis, CA 95616, USA; 4 UC Davis Genome Center, University of California, One Shields Ave., Davis, CA 95616, USA; 5 Nestlé Research Centre, Vers-chez-les-Blanc CH-1000, Lausanne 26, Switzerland

## Abstract

**Background:**

The molecular events underlying mammary development during pregnancy, lactation, and involution are incompletely understood.

**Results:**

Mammary gland microarray data, cellular localization data, protein-protein interactions, and literature-mined genes were integrated and analyzed using statistics, principal component analysis, gene ontology analysis, pathway analysis, and network analysis to identify global biological principles that govern molecular events during pregnancy, lactation, and involution.

**Conclusion:**

Several key principles were derived: (1) nearly a third of the transcriptome fluctuates to build, run, and disassemble the lactation apparatus; (2) genes encoding the secretory machinery are transcribed prior to lactation; (3) the diversity of the endogenous portion of the milk proteome is derived from fewer than 100 transcripts; (4) while some genes are differentially transcribed near the onset of lactation, the lactation switch is primarily post-transcriptionally mediated; (5) the secretion of materials during lactation occurs not by up-regulation of novel genomic functions, but by widespread transcriptional suppression of functions such as protein degradation and cell-environment communication; (6) the involution switch is primarily transcriptionally mediated; and (7) during early involution, the transcriptional state is partially reverted to the pre-lactation state. A new hypothesis for secretory diminution is suggested – milk production gradually declines because the secretory machinery is not transcriptionally replenished. A comprehensive network of protein interactions during lactation is assembled and new regulatory gene targets are identified. Less than one fifth of the transcriptionally regulated nodes in this lactation network have been previously explored in the context of lactation. Implications for future research in mammary and cancer biology are discussed.

## Background

Lactation is one of the most remarkable products of evolution. The signature feature and basis of the competitive emergence of mammals, including humans, is the production of complete early nourishment of neonates by the mother. The processes of lactation include the development of mammary tissue, as well as the synthesis and secretion of milk. At weaning, the mammary gland morphologically returns to a near pre-pregnant state. Thus, in addition to the important nutritional implications, lactation provides a model for basic biological processes such as the proliferation, differentiation, survival and death of cells.

Although lactation is believed to be a product of Darwinian selective pressure, little is known of its molecular origins or its regulation. Current knowledge of the molecular regulation of mammary development and lactation has largely been derived from dissection of signaling networks in cell culture systems and phenotypic characterization of genetically altered mice. Some proteins modulated during pregnancy and lactation have been identified and characterized in the context of hormonal and metabolic pathways (reviewed in [1,2]). Beyond these signaling pathways, the regulation of mammary gland development and lactation is incompletely understood. Of particular interest are the major molecular events that govern macroscopic and histological changes in the mammary gland during secretory differentiation, secretory activation (the lactation switch), and the onset of involution (the involution switch). Unbiased genome-wide approaches are likely to identify novel genes and gene products involved in the regulation of lactation, particularly when incorporated into a larger picture of mammary development and function.

In this study, bioinformatic techniques are applied to transcriptomic and proteomic data to enhance understanding of how the mammary gland is regulated through pregnancy, lactation, and involution. Using non-hypothesis-driven analyses, transcriptional and post-transcriptional trends are described and putative key regulatory targets are identified. Gene products and their interactions unexplored in the current literature are visualized as a network, providing a framework on which to base future research. Such exploratory methods can be applied to other areas of biological inquiry to establish a quantitative representation of current knowledge and to facilitate the generation of new hypotheses.

## Results

### Global transcriptional trends during mammary development

**Figure 1:**
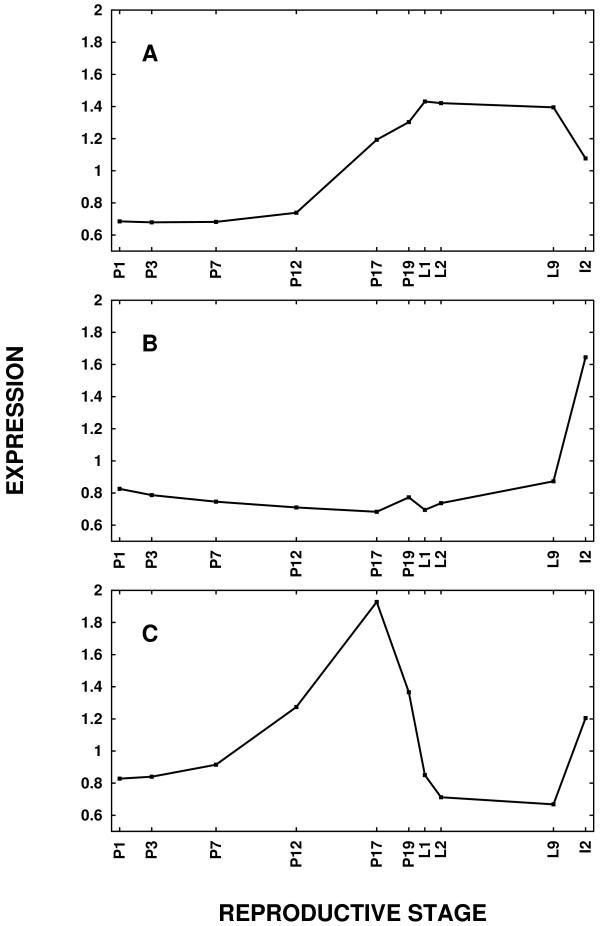
**The major trend is a gradual increase in gene expression from mid to late pregnancy**. The (A) first, (B) second, and (C) third principal components of genome-wide gene expression data from early pregnancy through involution explain (A) 50.0%, (B) 13.6%, and (C) 11.6% of the variance, respectively.

Using microarray data from the Neville study [3] (see Methods), a statistical analysis of genome-wide transcriptional changes in the mammary gland was applied to identify 4,832 genes differentially expressed (*p *< 0.001) of the 12,488 measured during a full mouse lactation cycle. To understand the major trends in gene transcription across developmental stages of the mammary gland from initial pregnancy to involution, a principal component analysis with mean centering and scaling was applied to these differentially expressed genes across all ten time points. (For descriptions of these time points, see Materials and methods.) The top three principal components of the data in the time domain are diagrammed in Figures 1A–C. The first principal component describes 50.0% of the variance in the data. This major trend is a rise in gene expression during late pregnancy that remains high during lactation and falls during involution. A substantial set of genes – 592 – has a standard correlation of 0.90 or better with this first principal component (Additional data file 1). The second and third principal components appear to be minor trends, explaining 13.6 and 11.6% of the variance in the data. In the second principal component (Figure 1B), expression is unchanged during pregnancy and lactation, but rises during involution. In the third principal component (Figure 1C), gene expression rises in late pregnancy, is depressed during lactation, and then rises again during involution. Few genes, twelve and three, respectively, have a standard (uncentered) correlation of 0.90 or better with the second and third principal components. Each of the remaining principal components explains less than 10% of the variance in the data.

Because few genes are strongly correlated with the second and third principal components, other gene lists associated with these trends may be of greater biological interest. For the second component, the 2,064 genes that are statistically up-regulated during involution day 2 (I2) compared to lactation day 9 (L9) are provided as part of Additional data file 5. With respect to the third principal component, the 634 genes that are down-regulated by lactation day 2 (L2) relative to pregnancy day 17 (P17) and up-regulated by I2 relative to L9 are provided in Additional data file 2.

How do these principal components in Figure 1 relate to the known histology (reviewed in [4,5]) of the mouse mammary gland? In the first part of pregnancy, the epithelial compartment of the gland undergoes a remarkable expansion, from less than 10% of the total volume to about 90% by birth. This proliferative activity is greatest prior to time point pregnancy day 12 (P12). The rise between pregnancy day 1 (P1) and P12 in the third principal component may reflect an increase in expression of epithelial genes due to the increase in this cell population. Secretory differentiation, characterized by an increase in lipid droplets in the cells, begins about mid-pregnancy and continues through P17, at which point the droplets mostly fill the alveolar cells until parturition, pregnancy day 19 (P19), when the lipid droplets move into the lumen representing the first onset of milk secretion. Approximately half of the variance in the microarray data can be described by the first principal component (see Figure 1A) in which gene expression increases from P12 to P19. This suggests that the transcriptional changes necessary to prepare the gland for secretion during this time period are the strongest trend across the entire developmental cycle. Soon after parturition, P19, the lipid droplets disappear from the lumen as milk is removed by the suckling offspring and the gland begins copious milk production. Lactation reaches full maturation by about lactation day 7 and certainly by time point L9. The third principal component implies that the transcription of a subset of genes may be suppressed during lactation.

At weaning, the mammary gland returns to the pre-pregnant state within four to ten days [6]. In the microarray study, the I2 time point may be interpreted as early involution because it occurred two days after the pups were removed. The second principal component shows a sharp upward trend in gene expression between L9 and I2. Very few genes are strongly correlated with this principal component, despite the fact that over 2,000 genes are statistically up-regulated between L9 and I2. This suggests that these genes that are markedly induced during involution are not coordinately regulated during pregnancy or lactation. In other words, involution represents a totally new phase of mammary development that follows a pathway distinct from the pathways that lead to milk secretion.

To investigate the biological functions represented by the first principal component, gene ontology (GO) analysis was applied to the 592 genes with a standard correlation of 0.90 or better with the component's profile (Additional data file 1). Non-synonymous enriched GO terms that describe the annotated biological processes represented by these genes are listed in Table 1 in statistically ranked order. Some of the annotated functions associated with this profile are consistent with known functions of mammary epithelial cells (MECs) during lactation – ion transport, calcium-mediated signaling, transferase activity, and cell proliferation. It is interesting that genes associated with these known functions appear to be transcribed well in advance of the onset of lactation. The remaining GO terms in Table 1 suggest that some genes that have been annotated with respect to immune cells are important to the development of the mammary gland during late pregnancy.

**Table 1:** First principal component, significant biological processes

GOID	GO Term
45410	Positive regulation of interleukin-6 biosynthesis
50850	Positive regulation of calcium-mediated signaling
42108	Positive regulation of cytokine biosynthesis
50671	Positive regulation of lymphocyte proliferation
51347	Positive regulation of transferase activity
30001	Metal ion transport

The genes correlated with the first principal component were also analyzed for enrichment in metabolic and signaling pathways. None of the 152 canonical pathways were statistically significant (*p *< 0.05) or even marginally significant (unadjusted *p *< 0.05). It is likely that these genes that are coordinately regulated during mammary development participate in pathways that are not yet annotated.

**Figure 2:**
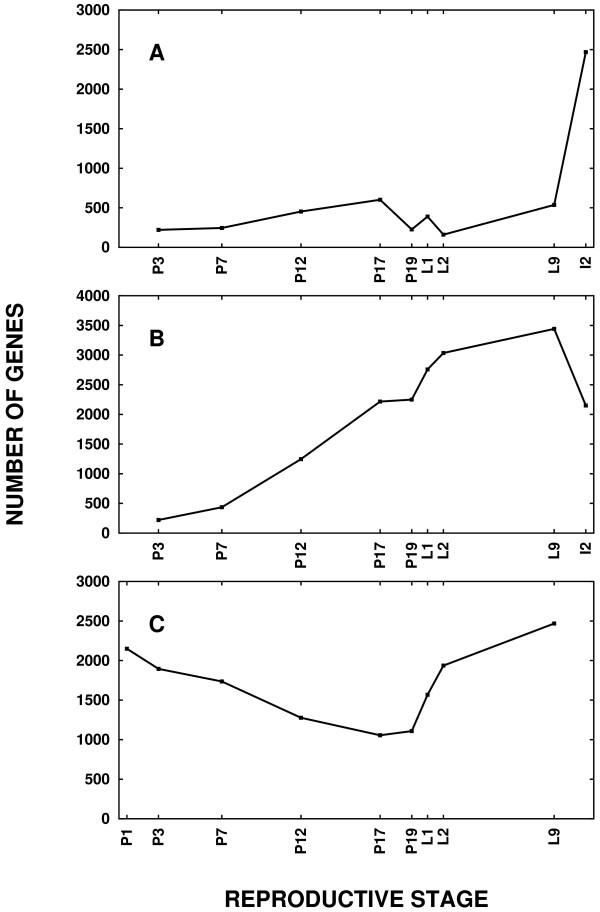
**Genome-wide transcriptional changes occur at the involution switch, but not at the lactation switch**. (A) Each data point represents the number of genes that are differentially transcribed relative to the previous time point. Five times as many transcriptional changes occur between full lactation, L9, and involution, I2, compared to any other transition between time points. (B) Each data point represents the number of genes that are differentially transcribed at that reproductive stage relative to early pregnancy, time point P1. These transcriptional changes suggest a gradual divergence from early pregnancy that increases until full lactation, L9, with a possible convergence to the pre-lactation state at involution. (C) Each data point represents the number of genes that are differentially transcribed at that time point relative to early involution, time point I2. The closest transcriptional state to I2 is P17. Of the 2,468 genes with statistically different expression in the transition from L9 to I2, 1,688 of these genes are not different between P17 and I2. Therefore, the involution switch is characterized by a partial reversion to the pre-lactation transcriptional state.

Pairwise comparisons between developmental stages were examined to find other underlying trends in the data. The number of genes whose expression differs significantly from the previous time point is plotted in Figure 2A. Clearly, the transcriptional changes are very gradual across pregnancy until involution, suggesting that there is no sudden transcriptional switch at the time of parturition. However, there does appear to be a massive transcriptional change between full lactation and involution.

To assess cumulative transcriptional changes over the full lactation cycle, the number of genes whose expression differs significantly (*p *< 0.001) from the first time point, P1, is plotted in Figure 2B. The late pregnancy transcriptional state is quite divergent from the initial pregnancy state and the greatest extent of divergence has occurred by full lactation. This graph hints at the possibility of a convergence to the pre-lactation state at the time of early involution, but it does not directly test the number of genes different between the late pregnancy time points and involution. The number of genes whose expression differs significantly (*p *< 0.001) from the involution time point, I2, is plotted in Figure 2C. The time point with the closest transcriptional state to I2 is the late pregnancy time point, P17. Of the 2,468 genes with statistically different expression in the transition from L9 to I2 (Figure 2A), the expression of 1,688 of these genes is not different between P17 and I2. In other words, 68% of the transcriptional changes between L9 and I2 can be attributed to a partial return to the pre-lactation state. Conceptually, the MECs may be exploring new frontiers of the transcriptional state space to satisfy the unusual requirements of lactation, frontiers from which they must retreat before they can proceed with the involution program.

### Transcriptionally regulated biological functions during mammary development

Functions of genes clustered by transcriptional profiles during mammary development have been previously described [3,7,8]. Here, GO analysis is used to screen for additional unexpected transcriptionally regulated biological functions and trends during pregnancy, lactation, and involution (Tables 2, 3, and 4, respectively). To facilitate future study, lists of genes associated with the GO terms in Tables 2, 3, 4 are provided (Additional data files 7, 8, 9).

**Table 2:** Pregnancy Gene Set, significant biological processes

GO ID	GO Term
**UPREGULATED**	
48193	Golgi vesicle transport
45045	Secretory pathway
41	Transition metal ion transport
45047	Protein targeting to ER
46903	Secretion
6888	ER to Golgi vesicle-mediated transport
6810	Transport
51179	Localization
51234	Establishment of localization
	
**DOWNREGULATED**	
6817	Phosphate transport
15698	Inorganic anion transport
6820	Anion transport
6635	Fatty acid beta-oxidation
19395	Fatty acid oxidation
6631	Fatty acid metabolism
1944	Vasculature development
48514	Blood vessel morphogenesis
1568	Blood vessel development
7155	Cell adhesion
6082	Organic acid metabolism
19752	Carboxylic acid metabolism
9653	Morphogenesis

**Table 3:** Lactation Gene Set, significant biological processes

GO ID	GO Term
**DOWNREGULATED**	
46907	Intracellular transport
6886	Intracellular protein transport
9059	Macromolecule biosynthesis
6412	Protein biosynthesis
51649	Establishment of cellular localization
15031	Protein transport
6635	Fatty acid beta-oxidation
51641	Cellular localization
19538	Protein metabolism
6878	Copper ion homeostasis
16043	Cell organization and biogenesis
6512	Ubiquitin cycle
45184	Establishment of protein localization
8104	Protein localization
245	Spliceosome assembly
398	Nuclear mRNA splicing, via spliceosome
377	RNA splicing, via transesterification reactions with bulged adenosine as nucleophile
375	RNA splicing, via transesterification reactions
30163	Protein catabolism
8380	RNA splicing
43632	Modification-dependent macromolecule catabolism
19941	Modification-dependent protein catabolism
43170	Macromolecule metabolism
19395	Fatty acid oxidation
44249	Cellular biosynthesis
6511	Ubiquitin-dependent protein catabolism
44267	Cellular protein metabolism
44260	Cellular macromolecule metabolism
44257	Cellular protein catabolism
51603	Proteolysis during cellular protein catabolism
16071	mRNA metabolism
9058	Biosynthesis
51170	Nuclear import
17038	Protein import
43285	Biopolymer catabolism
19883	Antigen presentation, endogenous antigen
30036	Actin cytoskeleton organization and biogenesis
51169	Nuclear transport
30333	Antigen processing
6913	Nucleocytoplasmic transport
6397	mRNA processing
6606	Protein import into nucleus
43037	Translation
6413	Translational initiation
6605	Protein targeting
7010	Cytoskeleton organization and biogenesis

**Table 4:** Involution Gene Set, significant biological processes

GO ID	GO Term
**UPREGULATED**	
6512	Ubiquitin cycle
16043	Cell organization and biogenesis
46907	Intracellular transport
51649	Establishment of cellular localization
51641	Cellular localization
43632	Modification-dependent macromolecule catabolism
19941	Modification-dependent protein catabolism
6886	Intracellular protein transport
15031	Protein transport
6878	Copper ion homeostasis
6511	Ubiquitin-dependent protein catabolism
8104	protein localization
45184	Establishment of protein localization
30163	Protein catabolism
16192	Vesicle-mediated transport
43285	Biopolymer catabolism
15992	Proton transport
6979	Response to oxidative stress
245	Spliceosome assembly
19538	Protein metabolism
	
**DOWNREGULATED**	
48009	Insulin-like growth factor receptor signaling pathway
7595	Lactation
9309	Amine biosynthesis
44271	Nitrogen compound biosynthesis
8652	Amino acid biosynthesis
6220	Pyrimidine nucleotide metabolism
6084	Acetyl-CoA metabolism
44237	Cellular metabolism
8152	Metabolism
9058	Biosynthesis
44249	Cellular biosynthesis
9117	Nucleotide metabolism

#### Pregnancy Gene Set: early pregnancy (P1) to parturition (P19)

By the end of pregnancy, P19, 985 genes are up-regulated and 1,265 genes are down-regulated relative to the start of pregnancy, P1. To screen for transcriptionally modulated biological functions during pregnancy, this Pregnancy Gene Set (see Methods) was analyzed for enriched GO terms. Statistically significant biological process GO terms associated with genes up-regulated during pregnancy are listed in Table 2. Biological processes associated with genes up-regulated during this period are dominated by the synthesis, transport, localization, and secretion of proteins and by transport of ions. MECs appear to be involved in a massive operation of synthesizing and packaging materials before the onset of lactation. Eight of the nine enriched GO terms are related to protein transport, localization, and secretion. This analysis suggests that many of the genes for milk synthesis and secretion are transcribed prior to the onset of lactation.

One of the nutritional properties of milk is the co-delivery of mineral ions in excess of their normal poor bioavailability in aqueous matrices, implying that the processes of lactation must include substantial ion transport capabilities. GO analysis (Table 2) implies that the increased transcription of genes associated with ion transport begins during pregnancy. With respect to ion transport, both zinc and iron ion transport systems are up-regulated. However, some genes associated with iron transport are also down-regulated. *Ceruloplasmin *(Cp, [GenBank:U49430]), *ferritin heavy chain 1 *(Fth1, [GenBank:X52561]), *lactotransferrin *(Ltf, [GenBank:J03298]), *sideroflexin 2 *(Sfxn2, [GenBank:AA189555]), and *transferrin receptor *(Tfrc, [GenBank:X57349]) are up-regulated, while *ferritin light chain 1 *(Ftl1, [GenBank:L39879]), *hephaestin *(Heph, [GenBank:AF082567]), *hemochromatosis *(Hfe, [GenBank:Y12650]), *sideroflexin 1 *(Sfxn1, [GenBank:NM_027324]), and *solute carrier family 11 member 1 *(Slc11a1, [GenBank:L13732]) are down-regulated. As zinc, copper, and iron are preferentially channeled through the MECs into the milk [9], this transcriptional modulation of ion transport systems probably reflects this process.

Table 2 also lists statistically significant biological process GO terms associated with genes down-regulated during pregnancy. These GO terms point to three major functions – anion transport, fatty acid oxidation, and blood vessel development. 'Phosphate transport' is the most highly enriched GO term associated with genes down-regulated by late pregnancy. Genes annotated with this GO term are almost all pro-collagen genes, and it is this collagen-forming function that more accurately describes genes up-regulated during pregnancy than 'phosphate transport'. The 'phosphate transport' annotation appears to be out of context when applied to the mammary gland. It was previously suggested that down-regulation of collagen genes is either due to a reduction of the stromal compartment or the expansion of the epithelial compartment in the mammary gland [3]. Table 2 also lists 'fatty oxidation'. It is expected that oxidation of fatty acids would be down-regulated during lactation, and these results show that, consistent with prior study [3], this function is already down-regulated by late pregnancy.

Surprisingly, genes usually associated with vasculature development appear to be down-regulated, a finding that is not consistent with what is known to be true at the macroscopic level. The gland experiences significant angiogenesis during pregnancy as this process is a necessary prerequisite for lactation. In the rat, the vasculature doubles by mid-pregnancy through angiogenesis by sprouting and intersucception [10]. The 'angiogenesis' GO annotations are likely dictated by cancer research, so there may be genes in the genetic circuit controlling vascularization of normal mammary development that are not yet annotated as such. To facilitate further study, genes associated with these GO terms are provided in Additional files 2, 3, 4. Although some of the biological functions listed in Table 2 are expected given prior study [3], screening with GO terms indicates the following: some GO terms may generally be out of context when applied to the mammary gland, elements of the secretory pathway are up-regulated prior to parturition, and the vascularization of the normal mammary gland may be incompletely understood at the level of transcription.

#### Lactation Gene Set: parturition (P19) to mature lactation (L9)

Relative to parturition (P19), 122 genes are up-regulated and 1,704 are down-regulated by the time of mature lactation, L9. This Lactation Gene Set (see Methods) was analyzed for enriched GO terms. Surprisingly, no GO terms are statistically enriched among genes up-regulated during this time period. This leads one to question whether genes up-regulated during lactation are not annotated. However, both up-regulated and down-regulated probes are annotated to approximately the same level, 87 and 89%, respectively. It is possible that the gene set is too small to find statistically enriched terms, or that the genes up-regulated during lactation have disparate functions, or that the common functions have not yet been annotated for their lactation-specific functions.

Enriched GO terms associated with genes down-regulated during lactation relative to parturition are given in Table 3. As during pregnancy, beta-oxidation of fatty acids is suppressed. Because one of the major functions of the mammary gland during lactation is the synthesis and packaging of lipids, it would be expected that oxidative consumption of such lipids for energy by the same cells would be counterproductive. Surprisingly, the transcription of genes involved in protein transport is suppressed during lactation. Thus, the main functions of packaging and exporting proteins by the traditional secretory pathway during the latter part of pregnancy may be transcriptionally attenuated during lactation. As shown in Table 3, 'protein metabolism' – both biosynthesis and catabolism – has also been transcriptionally turned down during lactation. In particular, ubiquitin-dependent protein catabolism is suppressed. Likewise, genes annotated for 'spliceosome assembly' – a ribonucleoprotein apparatus that catalyzes nuclear mRNA splicing – are also down-regulated, implying that both mRNA and protein processing are essentially halted or dramatically slowed during lactation. However, synthesis of the major milk proteins is known to be highly up-regulated [3]. Thus, it is likely that a small subset of genes is specifically utilized by the lactating gland to process proteins for secretion.

Genes associated with 'antigen processing' or 'antigen presentation' are also statistically down-regulated during lactation relative to late pregnancy. Although the complete immune function of colostrum may not be known, it is well-accepted that colostrum, which would be expected to be functionally tied to the genes transcribed in late pregnancy, has substantial immune components. Perhaps these genes, or a subset of them, are unique to colostrum and thus, their transcription is switched off by the time of full lactation. In summary, the lactation period appears to be characterized by widespread suppression of transcription across many functional classes.

#### Involution Gene Set: mature lactation (L9) to involution (I2)

By involution, time point I2, 2,064 genes are up-regulated and 404 genes are down-regulated relative to mature lactation, L9. GO analysis of this Involution Gene Set (see Methods) yields enriched GO terms for both up-regulated and down-regulated genes (Table 4). Seventeen of the twenty enriched GO terms (85%) associated with up-regulation during involution exactly match GO terms of the genes down-regulated during lactation. Transcriptionally, the switch to involution largely reverses the state of lactation. Genes involved in protein transport are transcribed again, re-enabling the traditional secretory pathway, for example. Protein catabolism is up-regulated, an expected major function during involution of the mammary gland. The 'response to oxidative stress' GO term is probably associated with the massive protein catabolism that is taking place, either to protect the MECs or the surrounding cells. Genes involved in metal ion homeostasis, especially copper, are also transcribed again.

Proton transport is up-regulated during involution. Because both ATP synthesis-coupled and ATP hydrolysis-coupled proton transport are up-regulated, the up-regulation of these genes is probably not reflective of an energetic transition of the mammary cells. It is possible that this set of genes is up-regulated to facilitate the degradation of milk components and the transfer of the small molecules to the interstitial compartment.

Far fewer genes, only 404, are down-regulated during involution. Nevertheless, a few clustered biological functions are represented (Table 4). In general, biosynthetic and metabolic processes are down-regulated, as would be expected of involuting cells. Overall, early involution appears to be characterized by a suppression of specific lactation functions and up-regulation of genes associated with novel functional categories.

### Transcriptionally regulated pathways during mammary development

To identify which of the known metabolic and signaling pathways are transcriptionally regulated, pathway analysis was applied to the Pregnancy, Lactation, and Involution Gene Sets (see Methods). Of the 152 canonical pathways evaluated, 16 are marginally significant during pregnancy, 22 during lactation, and 40 during involution (unadjusted *p *< 0.05). When a multiple testing correction is applied (see Methods), no pathways are significant during pregnancy, 8 are significant during lactation, and 24 are significant during involution (*p *< 0.05). The complete list of pathways, their p-values, and the molecules regulated in those pathways are provided (Additional data file 19), as well as color-coded diagrams for the significant pathways (Additional data file 20, 21, 22). An interpretation of all of these pathways is well beyond the scope of this paper. Therefore, the three pathways that most significant during lactation are briefly explored in the following sections.

#### PI3K-AKT pathway

**Figure 3:**
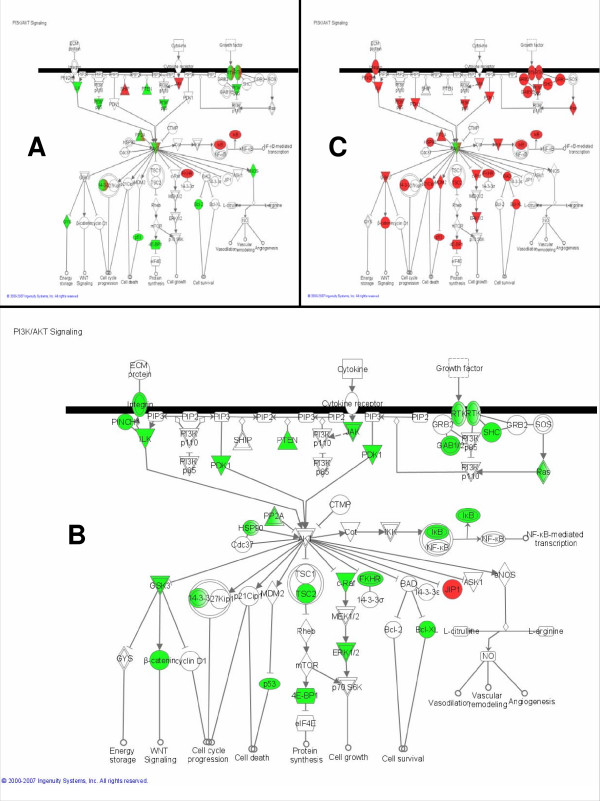
**The PI3K-AKT pathway is transcriptionally regulated throughout the lactation cycle**. The PI3K-AKT pathway is enriched with genes differentially regulated during (A) pregnancy, (B) lactation, and (C) involution. Green nodes are down-regulated, red nodes are up-regulated.

In this pathway analysis, the PI3K-AKT pathway (Figure 3) is the most significant transcriptionally regulated pathway during pregnancy. At the center of Figure 3A, the AKT node is a mixture of red and green because the members that comprise this node are differentially regulated. Akt1 [GenBank:X65687] is up-regulated, Akt2 [GenBank:U22445] is down-regulated, and calmodulin, which complexes with Akt2, is down-regulated. During early involution (Figure 3C): Akt1 is down-regulated while Akt2 is up-regulated. Studies with transgenic and knock-out mice illustrate differential physiological roles and tissue-specific functions for the two Akt isoforms [11]. It is possible that the differential regulation throughout the pathway seen during pregnancy (Figure 3A) is due to the heterogeneous cell populations of the mammary gland with Akt2 regulated in adipose tissue and Akt1 is regulated in epithelial cells. However, the two isoforms are reciprocally modulated during early involution when the epithelial cells are the predominant cell type, so it is also possible that the isoforms are co-regulated in the same tissue.

The PI3K-AKT pathway is highly significant during lactation (*p *= 0.0101) and involution (*p *< 0.0001) (Figure 3B–C). Thus, the pathway analysis suggests that Akt is a central regulator throughout the lactation cycle. Also, Akt1 appears to be the sole down-regulated protein in the pathway during involution, implying a critical regulatory role during this developmental stage. Experimental data supports these findings. Akt expression increases dramatically at the onset of lactation but is relatively low during involution [12]. In transgenic mice with constitutively active Akt1, involution is delayed, suggesting that Akt1 is an essential regulator of involution [13-15]. Interestingly, the overexpression of Akt1 in these mice during the pregnancy and lactation stages pathologically enhanced lipogenesis [12]. The fat content of the milk was 65–70%, compared to 25–30% in wild-type mice. Thus, experimental evidence supports Akt1 as a central regulator during all phases of lactation. In Figure 3, genes that are transcribed to form products upstream and downstream of Akt1 are also significantly transcriptionally regulated. The enrichment of the entire PI3K-AKT pathway for differentially regulated genes in the three time periods analyzed in this paper suggests that the entire pathway, and not solely Akt1, is coordinately regulated throughout the lactation cycle.

#### Integrin pathway

**Figure 4:**
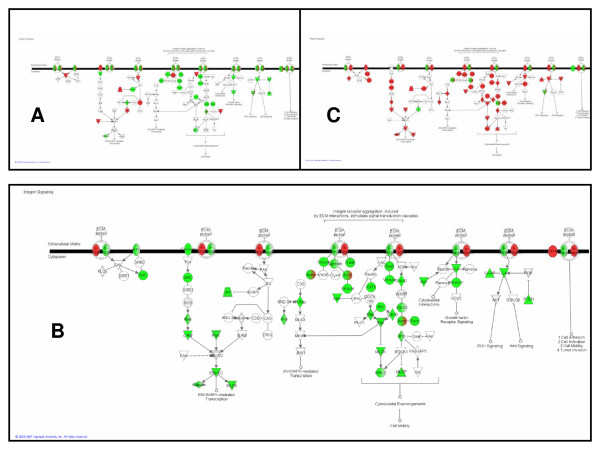
**Downstream components of the integrin pathway are broadly suppressed during lactation**. The integrin pathway is enriched with genes differentially regulated during (A) pregnancy, (B) lactation, and (C) involution. Green nodes are down-regulated, red nodes are up-regulated.

Integrins are cell-surface receptors that attach the cell to the surrounding extra-cellular matrix and transduce signals from the matrix to the cell. Through integrins, the extra-cellular matrix influences the growth, differentiation, and survival of mammary epithelial cells [16]. In this analysis, the integrin pathway (Figure 4) is marginally significantly enriched for transcriptionally regulated genes during pregnancy (unadjusted *p *= 0.0063) and significantly enriched during lactation (*p *< 0.0001), and involution (*p *< 0.0001). During pregnancy, the two major tissue types of the mammary gland change dramatically: the adipose compartment shrinks while the epithelial cell compartment differentiates and proliferates. Thus, the pathway diagram in Figure 4A with both up- and down-regulated regulation of both the integrins and their downstream components is unsurprising given the opposite developmental programs of the heterogeneous cell populations. During lactation, the regulation of the integrin pathway (Figure 4B) illustrates down-regulation of the downstream components of integrins. In other words, signals coming from the extra-cellular matrix to the mammary epithelial cells are attenuated. This reflects a major change in integrin signaling and further implies that the primary function of MECs during lactation – milk synthesis – does not require much communication with surrounding environment. During early involution (Figure 4C), the downstream components of the integrin pathways are again up-regulated, perhaps reflecting a need for increased cell-environment communication to coordinate the complex tissue remodeling that characterizes this time period. However, the diagrams in Figure 4 may incompletely describe the status of the integrin pathway due to post-transcriptional regulation. Western blots of beta-integrins during early involution demonstrate a decrease in this protein [17], not the increase that is forecasted based on microarray data in Figure 4C. Nevertheless, significant enrichment of this pathway with transcriptionally regulated genes makes a strong argument for intense regulation of the integrin pathway throughout the lactation cycle.

#### Protein ubiquitination pathway

**Figure 5:**
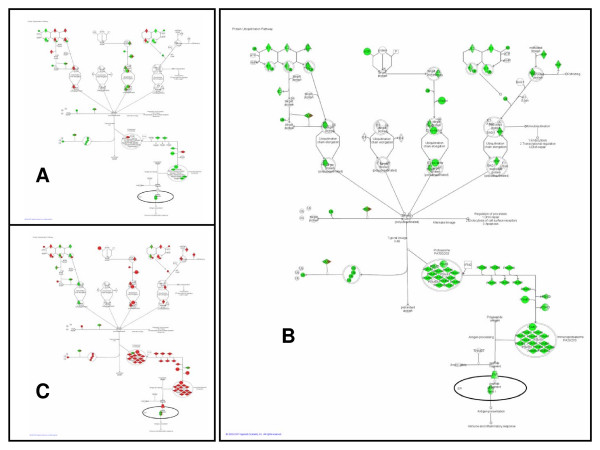
**Protein ubiquitination is the most significantly regulated pathway during both lactation and involution**. The protein ubiquitination pathway is not significantly enriched with differentially regulated genes during (A) pregnancy. However, it is the most significantly enriched pathway during (B) lactation, and (C) involution. Green nodes are down-regulated, red nodes are up-regulated.

The protein ubiquitination pathway (Figure 5) is not significantly enriched for transcriptionally regulated genes during pregnancy. However, it is the most significantly enriched pathway during both lactation and involution (*p *< 0.0001). The degradation of cell cycle regulators by ubiquitination is an essential part of normal cell cycle control and thus it has been studied in the mammary gland in the context of breast cancer. However, its significance in normal mammary biology is less obvious. A decline in proteosomal proteins in the mammary gland during lactation has been previously noted [3]. Here, the pathway diagram in Figure 5B demonstrates that the suppression of the protein ubiquitination pathway is nearly unanimous. Then, during involution, most of the same nodes are again up-regulated (Figure 5C). These results imply that mammary epithelial cells, in their highly specialized state during lactation, require complete suppression of ubiquitin-directed protein degradation. Furthermore, in terms of the total number of genes regulated within a pathway, the highest priority during early involution is to re-enable the degradation of proteins. In summary, modulation of the protein ubiquitination pathway is perhaps the most important innovation of lactating mammary epithelial cells.

### Biological principles of the lactation proteome

**Figure 6:**
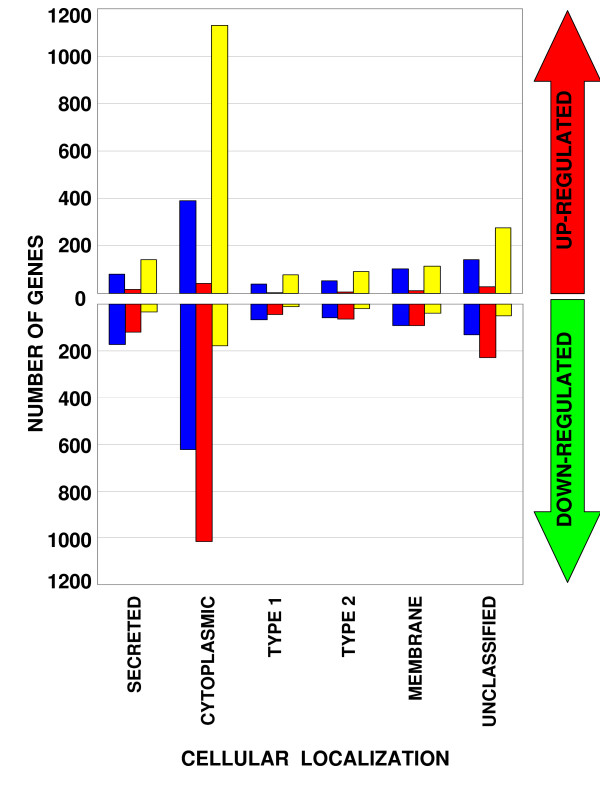
**Cellular localization data suggests a minority of secreted gene products during pregnancy and lactation**. Differentially transcribed genes are sorted by cellular localization. Blue columns, the first of each triad, represent genes from the Pregnancy Gene Set. Red columns, the second of each triad, represent the Lactation Gene Set. Yellow columns, the third of each triad, represent the Involution Gene Set. The top half of the graph displays up-regulated genes, while the bottom half displays down-regulated genes. 'CYTOPLASMIC' refers to soluble intracellular gene products. These proteins have no transmembrane domain or signal peptide. 'SECRETED' refers to soluble secreted gene products. These proteins have a signal peptide, but no transmembrane domains. 'TYPE I,' 'TYPE II,' and 'MULTIPASS' refer to type I, type II, and multipass membrane proteins, respectively. TYPE 1 proteins have one transmembrane domain and a signal peptide, TYPE II proteins have one transmembrane domain and no signal peptide, and MULTIPASS proteins have multiple transmembrane domains. 'UNCLASSIFIED' gene products are those that do not exist in the LOCATE database.

Because GO analysis suggested that components of the traditional secretory pathway are up-regulated prior to parturition, the potential mammary secretome was next explored. The Pregnancy, Lactation, and Involution Gene Sets were converted to mouse Swissprot IDs, which were used to query LOCATE, a mouse database of cellular localization data [18]. The cellular localization of the up-regulated and down-regulated genes across each developmental stage is tallied in Figures 6A and 6B, respectively.

Interestingly, more secreted proteins (transcripts with signal peptides) are down-regulated during pregnancy and lactation than are up-regulated. In fact, fewer than 100 transcripts with signal peptides are up-regulated during pregnancy or lactation. This suggests that the secreted proteins are limited to those important to milk composition and that other secretory functions are suppressed as the gland single mindedly pursues its lactational course. Thus, it is quite possible that the secretory pathway components serve primarily to synthesize and secrete large volumes of only a few proteins.

**Figure 7:**
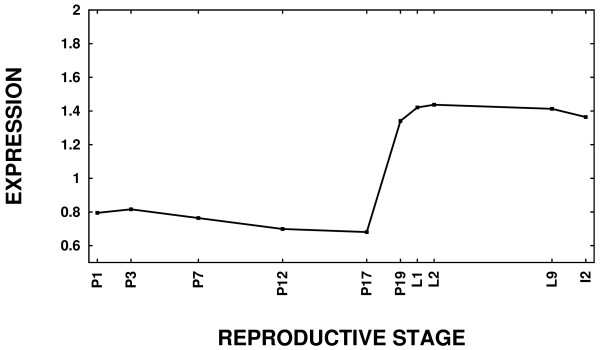
**Genes transcribed to form milk proteins are up-regulated sharply near parturition and sustained through involution**. Principal component analysis was applied to the major milk proteins (see text). The first principal component depicted here explains 54.8% of the variance.

The caseins are high-abundance proteins synthesized in the mammary gland. Rudolph *et al*. [3] studied the expression profiles of seventeen major milk protein genes: *alpha-casein *[GenBank:M36780], *beta-casein *[GenBank:X04490], *gamma-casein *[GenBank:D10215], *kappa-casein *[GenBank:M10114], *milk fat globule membrane protein E8 *[GenBank:M38337], *extracellular proteinase inhibitor *[GenBank:X93037], *adipose differentiation related protein *[GenBank:M93275], *whey acidic protein *[GenBank:V00856], *alpha-lactalbumin *[GenBank:M87863], *bile salt stimulated lipase *[GenBank:U37386], *lactotransferrin *[GenBank:J03298], *butyrophilin *[GenBank:U67065], *mucin 1 *[GenBank:M84683], *xanthine dehydrogenase *[GenBank:X75129], *casein delta *[GenBank:V00740], *epidermal growth factor *[GenBank:V00741], and *parathyroid hormone-like peptide *[GenBank:M60057]. Principal component analysis of these major milk proteins demonstrates that, as a class, these genes are up-regulated sharply near the time of parturition (Figure 7). All seventeen of these genes are among the up-regulated genes in the Pregnancy Gene Set, but only three of them are part of the Lactation Gene Set. Thus, both the statistical analysis and principal component analysis confirm that transcription of the major milk proteins has reached steady state by the onset of lactation.

### Protein interactions of genes expressed in the mammary gland

**Figure 8:**
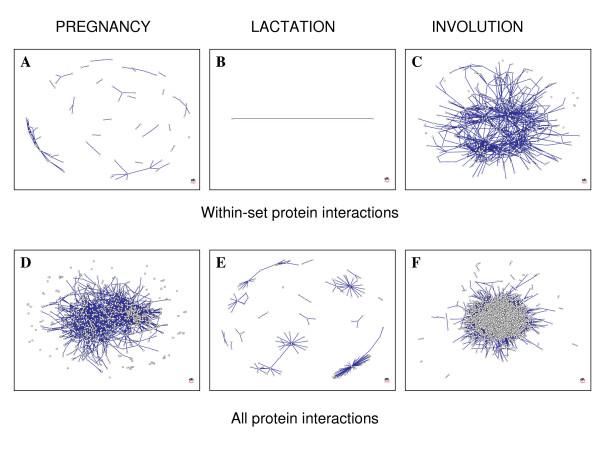
**Protein interaction networks represent differing post-transcriptional programs as a function of reproductive stage**. (A-C) In each network, nodes represent proteins whose genes are up-regulated in the (A) Pregnancy, (B) Lactation, and (C) Involution Gene Sets, while (A-C) links represent interactions with other proteins in the set. (D-F) In these networks, nodes represent either a protein whose gene is up-regulated in the (D) Pregnancy, (E) Lactation, or (F) Involution Gene Set, or (D-F) another protein not in the set that directly interacts with a protein in the set. (D-F) Links represent interactions between proteins in the set with each other or with other proteins not in the set. (A-F) All network figures were produced using the Fructerman-Reingold 3D layout algorithm. Three-dimensional animations of each network with labeled nodes are provided in Additional data files 11, 12, 13, 14, 15, 16. Spreadsheets of each protein-protein interaction are provided in Additional data file 10.

To characterize the interactions of proteins encoded by genes transcribed during mammary development, protein interaction networks were developed using the Pregnancy, Lactation, and Involution Gene Sets. Up-regulated genes from the three gene sets were converted from mouse Affymetrix probe identifiers to human UniProt/Swissprot Accessions and used to query the Online Predicted Human Interaction Database (OPHID) [19]. Custom scripts were written to create two types of networks for each gene set: (1) networks that contain only interactions of gene products with other gene products in the set ('within-set protein interactions' in Figure 8), and (2) networks that contain all known protein interactions with gene products in the set ('all protein interactions' in Figure 8). Each node in the networks represents a protein and each link between nodes represents a known protein-protein interaction from OPHID. Three-dimensional animations of these networks with labeled nodes and spreadsheets detailing all of the linkages are available in Additional data files 10, 11, 12, 13, 14, 15, 16.

Networks with few highly-connected nodes of high degree (i.e., with many links) and with most nodes of low degree (i.e., with few links) are considered to be scale-free. The networks in Figure 8A, C, and 8D–F all have degree distributions with similar shapes that appear to follow a power law, as would be expected of scale-free biological networks (data not shown). The degree distribution of the network in Figure 8B is, trivially, a single point.

From the network in Figure 8C, it is clear that genes up-regulated during involution do directly interact with each other at the protein level and are highly connected. Genes up-regulated by late pregnancy interact with each other to a limited extent (Figure 8A), either because there are fewer protein-protein interactions or because the protein interactions in which they are involved in mammary cells have not been observed in the high-throughput biology experiments used to derive protein interaction data. Few genes are up-regulated during lactation, and here (Figure 8B) there is no evidence that they directly interact with each other at the protein level. Again, some protein interactions may occur that are unique to mammary cells and would therefore not be present in these networks derived from OPHID.

The star patterns in Figure 8E suggest that a couple of the genes up-regulated during lactation directly interact with many other proteins. Network analysis indicates that these four lactation proteins are the most highly connected: KH domain-containing, RNA-binding, signal transduction-associated protein 1 (SAM68, [PIR:Q07666]), guanine nucleotide-binding protein subunit beta 4 (GBB4, [PIR:Q9HAV0]), C-jun-amino-terminal kinase-interacting protein 1 (JIP1, [PIR:Q9UQF2]), and insulin-like growth factor IB precursor (IGF1B, [PIR:P05019]). SAM68, GBB4, JIP1, and IGF1B interact with 35, 16, 12, and 11 proteins, respectively. Annotations in the UniProt database [20] provide possible functions for these proteins. SAM68 is an RNA binding protein that is involved in mRNA processing. GBB4 is a G protein likely involved as a modulator or transducer in various trans-membrane signaling systems. JIP1 may function as a regulator of vesicle transport, through interactions with the JNK-signaling components and motor proteins or as an anti-apoptotic protein. IGF1B is an insulin-like growth factor. In summary, the networks in Figure 8 suggest that the extent of interactions between proteins that are transcriptionally regulated may be a function of reproductive stage, with very few interactions occurring during lactation.

### Development of a lactation protein interaction network

To develop a more comprehensive lactation protein-protein interaction network, the scientific literature was mined for genes involved in lactation. Three gene sets – the Literature Gene Set (see Methods), up-regulated genes from the Pregnancy Gene Set, and up-regulated genes from the Lactation Gene Set – were combined and used to query the protein interaction database. Restricting the network only to interactions between proteins in the query list, this network contains 313 nodes and 438 linkages. Approximately 71% of the nodes belong to a single major component (subnetwork). The remaining nodes are associated with subnetworks consisting of 6 or fewer nodes each.

**Figure 9:**
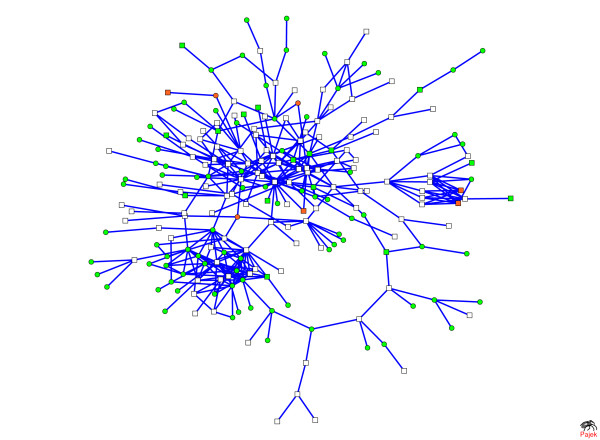
**Protein interactions during lactation are visualized in this major component of the comprehensive lactation network**. This network provides a visualization of putative protein interactions between genes known and not previously known to be involved in lactation and those that are and are not transcriptionally regulated. Nodes in this network are members of the Literature Gene Set or up-regulated members of the Pregnancy or Lactation Gene Sets that have protein interactions in OPHID with other members of these sets. Node shape indicates whether or not the gene product is represented in the lactation literature. Square-shaped nodes are gene products from the Literature Gene Set. Circle-shaped nodes are up-regulated gene products either the Pregnancy or Lactation Gene Set that are not in the Literature Gene Set. Node color indicates whether the gene product is up-regulated during pregnancy (green), or lactation (orange), or neither (white). Links between nodes represent protein-protein interactions listed in OPHID. A three-dimensional animation of this network with labeled nodes is provided in Additional data file 18; each protein interaction is detailed in Additional data file 17.

Viewing the major component (Figure 9), one can visualize interesting targets that are not already described in the literature. A three-dimensional animation of this network is available in Additional data file 18 with links described in Additional data file 17. Circles in Figure 9 represent those genes not in the lactation literature. Green circles are up-regulated by late pregnancy and orange circles are up-regulated by full lactation. Of the three transcripts up-regulated during full lactation that have not been previously described in the lactation literature, two of the proteins – D site-binding protein (DBP, [PIR:Q10586]) and thyrotroph embryonic factor (TEF, [PIR:Q10587]) – are transcription factors known to be related to circadian rhythms and the third, SAM68 [PIR:Q07666], is a mediator of RNA translation.

To understand what known functions are associated with this comprehensive lactation network, GO analysis was applied to the proteins in this network relative to all human UniProt/SwissProt Accessions available through Ensembl [21]. The top three non-synonymous GO terms enriched in the lactation network (*p *< 0.001) are 'JAK-STAT cascade,' 'anti-apoptosis,' and 'COPI coated vesicle membrane'. All might have been predicted from previous understanding of genes regulated during lactation.

Pathway analysis was also applied to the proteins in this lactation network. The pathways most significantly enriched (*p *< 0.005) with these proteins are the 'neuregulin', 'JAK/Stat', 'NF-kappaB', 'PI3K/AKT', 'IL-4', 'PTEN', and 'IGF-1' signaling pathways. In total, 38 pathways are marginally statistically enriched (unadjusted p < 0.05), 22 of which are significantly enriched (*p *< 0.05). However, no more than 20% of any one of these significant pathways is represented by nodes from the lactation network. These facts together suggest that proteins in this network are interacting in a way that is not well-characterized by any individual canonical signaling or metabolic pathways of this set of 152 curated pathways.

Hubs of a network are those nodes that are most highly connected. The hubs of the lactation network are listed in Table 5, ranked in order of connectivity. Hubs of protein interaction networks are the proteins most essential to the overall function of the network and they tend to be evolutionarily conserved [22]. In other words, small perturbations of the concentration or activation of these proteins can have large effects. The 27 hubs of this lactation network include receptors (EGFR, GHR, INSR, NR3C1, SMAD2), signaling proteins (JAK2, PTPN6, ERBB2, SOCS1, SOCS3, IRS1), transcription factors (STAT5A, STAT3, CEBPB), translation initiation factors (EIF4E, EIF3S4), as well as proteins involved in metabolism (OAT, CBS, SARS, PGD, HDLBP), vesicle formation (ATP6V1E1, COPB2, SEC23B), DNA repair (BRCA1), gene silencing (HAT1), and protein degradation (UFM1). Additional data file 23 contains manually annotated general and mammary-specific functions of these hubs. Many of these hub proteins have functions described in the scientific literature, but their annotations are either not descriptive of their role in mammary development or their function has not been evaluated in the context of mammary development. Unannotated hubs from the table in additional data file 23, (CBS, ATP6V1E1, PGD, HDLBP, COPB2, PTPN6, SARS, SEC23B, HAT1, UFM1, and EIF3S4), are prime candidates for future exploration.

**Table 5:** Major hubs of the comprehensive lactation network

No. of Links	UniProt Accession/HGNC Symbol	Protein Name
21	[PIR:P00533]/EGFR	Epidermal growth factor receptor precursor
17	[PIR:P04181]/OAT	Ornithine aminotransferase, mitochondrial
14	[PIR:P35520]/CBS	Cystathionine beta-synthase
13	[PIR:O60674]/JAK2	Tyrosine-protein kinase JAK2
12	[PIR:P04626]/ERBB2	Receptor tyrosine-protein kinase erbB-2 precursor
12	[PIR:P40763]/STAT3	Signal transducer and activator of transcription 3
12	[PIR:P36543]/ATP6V1E1	Vacuolar ATP synthase subunit E
11	[PIR:P38398]/BRCA1	Breast cancer type 1 susceptibility protein
11	[PIR:P42229]/STAT5A	Signal transducer and activator of transcription 5A
11	[PIR:P52209]/PGD	6-phosphogluconate dehydrogenase, decarboxylating
11	[PIR:Q00341]/HDLBP	Vigilin
11	[PIR:P35606]/COPB2	Coatomer subunit beta
10	[PIR:P10912]/GHR	Growth hormone receptor precursor
10	[PIR:P29350]/PTPN6	Tyrosine-protein phosphatase non-receptor type 6
10	[PIR:P06730]/EIF4E	Eukaryotic translation initiation factor 4E
10	[PIR:P49591]/SARS	Seryl-tRNA synthetase, cytoplasmic
10	[PIR:Q15437]/SEC23B	Protein transport protein Sec23B
10	[PIR:O14929]/HAT1	Histone acetyltransferase type B catalytic subunit
9	[PIR:O14543]/SOCS3	Suppressor of cytokine signaling 3
9	[PIR:P35568]/IRS1	Insulin receptor substrate 1
9	[PIR:O15524]/SOCS1	Suppressor of cytokine signaling 1
9	[PIR:Q15796]/SMAD2	Mothers against decapentaplegic homolog 2
9	[PIR:P61960]/UFM1	Ubiquitin-fold modifier 1 precursor
8	[PIR:P06213]/INSR	Insulin receptor precursor
8	[PIR:O75821]/EIF3S4	Eukaryotic translation initiation factor 3 subunit 4
8	[PIR:P17676]/CEBPB	CCAAT/enhancer-binding protein beta
8	[PIR:P04150]/NR3C1	Glucocorticoid receptor

## Discussion

### The lactome

The identification of all genes responsible for lactation was previously proposed [23]. However, the lactome is spatially and temporally complex. For successful lactation to occur, molecular events outside of the mammary gland would be expected to support maternal-offspring behaviors, offspring sucking and rooting reflexes, offspring digestive response to the milk, and so forth. If one limits the spatial scope to the mammary gland, this study demonstrates temporal complexity as well. Even with a very strict Bonferonni multiple testing correction (*p *< 0.001), nearly a third of the mouse transcriptome is in flux from pregnancy through involution. It is possible that some of these changes are due to the expansion and contraction of the epithelial compartment and concurrent proportional representation of the stromal cells. Ultimately, housekeeping genes of these changing cell populations may need to be separated from genes that are uniquely regulated in the context of the lactation apparatus. Despite these obvious limitations, an initial list of genes that comprise the lactome – those differentially regulated by late pregnancy relative to early pregnancy and by full lactation relative to late pregnancy – are provided with this study (see Pregnancy and Lactation Gene Sets in Methods).

### Annotation of the lactome

Gene ontology annotations are not necessarily applicable to the lactome as these annotations have arisen from studies in culture cells of other tissues. Here, the use of GO annotations provides a method to screen for unexpected biological functions (i.e., down-regulation of known angiogenesis-related genes during pregnancy) and to identify trends (i.e., reciprocal modulation of gene classes between lactation and involution). Annotation with respect to the specific biological context of lactation will ultimately be required. The comprehensive lactation protein interaction network derived in this paper provides a working model for future work on molecular interactions in mammary cells and highlights gene targets that have not yet been annotated for lactation in the literature. Only 17% of the regulated gene products in the network (Figure 9) co-occur in the literature with the keyword, 'lactation'. Even among the most central regulators – the hubs of the network in Table 5 – eleven of the 27 (40.7%) do not yet have a described role with respect to lactation or mammary development.

### The lactation switch

The transcriptional profiles of genes by functional class (i.e., milk proteins, lipid synthesis, etc.) have been well-described by a previous study [3], and the molecular events surrounding secretory activation have been recently reviewed [2]. This study illustrates, on a broader perspective, very gradual transcriptional changes with progression of reproductive stage until involution (Figure 2A). In other words, aside from small subsets of proteins such as the major milk proteins, there is no sudden transcriptional switch around the time of parturition. Preparations of the mammary gland for lactation include modifications to the transcriptional program, but the onset of lactation appears to be primarily controlled by post-transcriptional mechanisms.

Regulation of proteins upstream of mRNA transcription during lactation is not a new concept. In the presence of lactogenic hormones, the mRNA of casein genes accumulates rapidly due to increased RNA stability [24]. More recently, enhancement of translation of the mRNA of the beta-casein protein was found to occur by lengthening the poly(A) tract via cytoplasmic polyadenylation synergized by the activity of both prolactin and insulin signaling [25]. The rate of translation has also been found to be reduced by amino acid deprivation [26].

This study provides further targets for exploration of post-transcriptional regulation in the mammary gland. Of the mere 82 genes up-regulated during lactation relative to late pregnancy, the gene with the most known interactions at the protein level – the hub of the network with the highest number of links – is SAM68, an RNA binding signal transduction protein. SAM68, also known as KHDRBS1, is a putative regulator of mRNA splicing, translation, and nuclear export [27-29] and has recently been shown to play a role in regulation of apoptotic genes [29]. SAM68, therefore, has the potential to be an important post-transcriptional regulator of both milk secretion and mammary cell survival during lactation. Other potential post-transcriptional mediators of lactation exist among the hubs of the comprehensive lactation network: the eukaryotic translation initiation factors 4E (IF4E) and 3 subunit 4 (IF34). Long *et al*. hypothesized that IF4E may effect translation of certain mRNA or the acceleration of overall protein synthesis [30]. The existence of IF4E as a hub in the lactation network certainly supports a central regulatory role.

Network analysis in this study demonstrates that the proteins encoded by genes transcriptionally up-regulated in the mammary gland during lactation do not interact with each other. Thus, regulation in MECs may substantially occur through protein-protein interactions of gene products that pre-exist in the cell. To some extent, the differences seen in network connectivity, density, and shape may be an artifact of gene set size. There are roughly twice as many genes up-regulated during involution as there are during pregnancy and more than ten times as many compared to lactation. Also, because protein interaction data is derived by high-throughput screens of protein interactions in other cell systems, it is possible that unique protein interactions exist in the mammary gland have not yet been observed.

### Mammary epithelial cells during lactation

During lactation, MECs are essentially biofactories of lipids, proteins, and carbohydrates for milk. The transcriptional trends highlighted in this study imply that these cells become biofactories not by gain of function, but by a broad suppression of function to effectively push all of the cell's resources towards a very few important tasks such as the massive synthesis of lipids and of a large quantity of a minority of proteins. During lactation, MECs are highly specialized, inhabiting an extreme transcriptional state space that is the most divergent from the early pregnant state. The near-universal attenuation of integrin signaling suggests that MECs isolate themselves from their environment while they carry out their singular agenda. The striking suppression of the proteolytic machinery in both the GO and pathway analysis, particularly the ubiquitination pathway, suggests that an early step in mammary evolution was the acquisition of tolerance for such quantities of protein structures beyond that normally acceptable within cells. Such a suppression of proteolysis may have been necessary to allow the emergence of nutritionally functional proteins, particularly those with little stabilizing secondary and tertiary structure. MECs during lactation are truly unique and could serve as useful model for biological questions that transcend mammary biology.

### Secretory diminution

Secretory diminution is the gradual decline in milk production after peak lactation has been attained. The fact that the machinery for milk synthesis appears to be in place by the time of late pregnancy lends evidence towards a new theory of secretory diminution. With the genes contributing to the machinery down-regulated during lactation, the machinery is not replenished as it degrades over time. Thus, secretory diminution is neither programmed nor pathological as recently reviewed [31]; rather, it is an inevitable feature of this biological design.

### The involution switch

Using microarray technology, transcriptional profiles during involution have been studied by numerous groups [7,8,32,33]. Here, analysis of the transcriptome more clearly demonstrates that a massive transcriptional switch occurs between lactation and early involution (Figure 2A). Five times as many transcripts are differentially regulated at weaning relative to other times in the development of the gland from the onset of the pregnancy. Conceptually, a genome-wide transcriptionally-driven switch at involution may be biologically necessary because the timing of weaning is determined by the offspring and therefore, is unpredictable. Thus, the mammary gland cannot make gradual preparations for its onset. The signals responsible for this event are likely to be of interest for many biological processes particularly related to tissue remodeling.

The analysis in this study implies that transcriptional changes during early involution may function, in part, to revert the mammary gland to the pre-lactation state. First, the state space of the transcriptome gradually diverges from the onset of pregnancy through lactation and then, at involution, the transcriptome partially converges to the pre-lactation state (Figure 2B). More than half of the transcriptional changes during the transition from lactation to early involution bring the gland back to the transcriptional state of late-pregnancy. Second, GO analysis demonstrated that functionally, the transcriptional state is largely a reversion to the pre-lactation state – 85% of the enriched GO terms associated with up-regulation during involution exactly match GO terms of the genes down-regulated during lactation. To study involution, prior syntheses of microarray data have been applied exclusively to involution time points [34]. However, comparison of these involution time points to transcriptional changes during pregnancy should improve the ability to sort the many signals that are multiplexed during this very complex time period.

The transcriptional state space of a full lactation cycle was previously explored by Master et al using a three-dimensional visualization of the dimensionality-reduced pairwise distances between developmental time points [7]. The observation in this study that the state space of the transcriptome gradually diverges from the onset of pregnancy through lactation is consistent with their model. However, the observed partial convergence of the early involution transcriptional state towards the pre-lactation state is not suggested by their visualization. There are a couple of possibilities for this discrepancy. It is possible that the early involution time point is much closer to the near-parturition time point in their model than appears on the page in two dimensions. Second, the shape of the entire trajectory may be overly constrained in low dimensions such that the goodness-of-fit between any two of the developmental time points is compromised. In this study, for the particular question of whether the early involution transcriptional state is a partial reconvergence to the pre-lactation state, the pairwise differences are directly measured so the observed distances are unequivocal.

The network of interactions between proteins that are encoded from genes up-regulated during early involution is well-connected. This suggests that the process of early involution is coordinately regulated. That is, genes up-regulated together produce proteins that interact with each other. Furthermore, the cellular processes are unlikely to be unique to involution of the mammary gland because they are described by protein interaction data derived from other cell cultures. The protein interaction network at the time of involution (Additional data file 16) provides a roadmap for future molecular studies of involution.

### The milk proteome

GO analysis in this study suggests that much of the machinery for the secretory pathway is transcribed prior to lactation. Because very few genes are up-regulated during lactation relative to late pregnancy, this secretory pathway is likely devoted to the secretion of large amounts of a few proteins. Highly expressed milk protein genes, such as the caseins, account for as much as 30% of total RNA (personal communication, MC Neville). As a group, genes transcribed to form these major milk proteins are up-regulated sharply around the time of parturition.

Assessments of the number of unique proteins in the milk proteome differ due to technical limitations in available proteomic technologies. In this study, fewer than 100 genes whose proteins are destined for secretion were up-regulated during either pregnancy or lactation. This suggests that the endogenous portion of the milk proteome that is synthesized through the secretory pathway is derived from fewer than 100 transcripts. Some proteins such as immunoglobins, transferrin, and albumin enter the milk by the transcytosis pathway [35-37]. Thus, if the diversity of the milk proteome proves to be substantially greater than 100 proteins, that diversity is due to either to post-translational modifications of these 100 transcripts or to the import by the gland of many more exogeneous proteins than previously realized.

The small number of up-regulated transcripts with signal peptides also suggests that secretory functions not related to lactation are turned off while only those important to milk composition are retained. This biological principle is consistent with the view that the MECs have reached a very specialized transcriptional state that is single mindedly devoted to milk secretion.

### Implications for cancer research

During pregnancy, lactation, and involution, nearly a third of the mouse transcriptome is in flux within the mammary gland during which the proliferation, differentiation, and death of cells are exquisitely controlled. The survival of mammalian species depends on the ability of these cells to traverse this vast transcriptional state space repeatedly without malignancy. As such, normal mammary development provides a rich model system for the study of cellular development and the effects of perturbation.

Intriguingly, GO analysis revealed that genes known to be involved in angiogenesis in other tissues are, as a class, down-regulated during pregnancy. On a macroscopic level, the vascularization of the gland clearly increases. In the rat, the vasculature doubles by mid-pregnancy [10]. The 'angiogenesis' GO annotations are likely dictated by cancer research, so there may be genes that are important to angiogenesis in normal mammary development that are not yet annotated as such. Angiogenesis within the normal developing mammary gland may fundamentally differ from that of the neoplastic transformation. Other evidence of this hypothesis is provided by HIF1alpha. In human tumors, HIF1alpha was up-regulated, particularly in breast tumors that exhibit high rates of proliferation[38,39]. However, in the HIF1alpha null mouse mammary gland, vasculature development was unchanged [40]. Taken together, these studies imply that the angiogenesis of breast cancer is not merely the untimely enabling of the normal mammary vascularization circuit.

## Limitations

The techniques and data used in this study have several limitations worth noting. First, principal component analysis reduces the dimensionality of a data set by retaining components of the data that contribute most to its variance. These lower order components are generally the most important; however, it is possible that these components are unimportant or that the discarded higher order components are biologically relevant. Second, the application of GO analysis is unlikely to completely describe the function of genes transcribed during mammary development as many genes have not yet been annotated in this biologically unusual context. Third, the statistical tests commonly available to assess the significance of enrichment in the GO and pathway analyses are anti-conservative and based on flawed assumptions [41]. A multiple testing correction was used to mitigate the former, but solutions for the latter are not readily available. Also, enrichment of small gene sets, such as the set of genes that form products of the JAK-Stat pathway, are overly sensitive to small perturbations in the gene set under test, a property that will be inherent to any statistical test of enrichment. Fourth, the network analysis in this study is limited by the integrity of the underlying data. Protein interaction data, derived largely from large-scale yeast two-hybrid experiments, is known to contain both false positives and missed interactions [42]. It is also highly likely that protein interactions important to mammary development are incompletely represented by experiments with other cell types. Lastly, the microarray chips utilized were limited to 12,488 probes so it is possible that some differentially regulated transcripts were not measured.

## Conclusion

Bioinformatic techniques were applied to various data sets to derive global biological principles governing the molecular events in the mammary gland during pregnancy, lactation, and involution. The key biological principles identified include the following: (1) nearly a third of the transcriptome fluctuates to build, run, and disassemble the lactation apparatus; (2) genes encoding the secretory machinery are transcribed prior to lactation; (3) the diversity of the endogenous portion of the milk proteome is derived from fewer than 100 transcripts; (4) while some genes are differentially transcribed near the onset of lactation, the lactation switch is primarily post-transcriptionally mediated; (5) the secretion of materials during lactation occurs not by up-regulation of novel genomic functions, but by widespread transcriptional suppression of functions such as protein degradation and cell-environment communication; (6) the involution switch is primarily transcriptionally mediated; and (7) during early involution, the transcriptional state is partially reverted to the pre-lactation state. These guiding principles begin to establish a road map for future study of mammary development during pregnancy, lactation, and involution.

The seeds for new hypotheses are also provided in this study. First, secretory diminution, usually attributed to programmed cell death or oxidative damage, may actually be due to lack of replenishment of the secretory machinery at the mRNA level. GO analysis in this analysis suggests that once lactation has commenced, transcription of RNA to maintain the secretory machinery is not sustained. It is, therefore, inevitable that milk production will gradually decrease over time. Second, angiogenesis-related genes are down-regulated during pregnancy, while vasculature in the gland clearly increases. This suggests that the angiogenesis of breast cancer is fundamentally pathological, rather than an inappropriate launch of normal vascularization processes.

In addition to the biological principles derived in this paper, extensive supplementary materials are provided as a mineable resource for mammary biologists. The 22 additional data files include sets of differentially expressed genes, associated GO terms, libraries of annotated pathway diagrams, and developmental stage-specific protein interaction networks. The comprehensive lactation network of protein interactions assembled in this study prioritizes regulatory gene targets for future study and provides a framework from which to base future research. The network enables the visualization of connectivity between those gene products known to be involved in lactation and those previously unexplored in the lactation literature. These integrative methods can be applied to other areas of biological inquiry to establish a snapshot of current knowledge and to facilitate the generation of new hypotheses.

## Methods

### Microarray study design

This microarray study was previously described in Rudolph *et al*. [3]. Briefly, mammary gland RNA samples were collected from FVB mice, isolated, and hybridized onto Affymetrix MG_U74Av2 chips. In total, ten time points in mammary development with four biological replicates for each time point were produced: P1, the day a vaginal plug was observed; P3, pregnancy day 3; P7, pregnancy day 7; P12, pregnancy day 12; P17, pregnancy day 17; P19, pregnancy day 19; L1, early lactation, the first day pups are observed in the cage, L2, lactation day 2; L9, lactation day 9; and I2, two days after pup removal on lactation day 9. Histologically, the mammary proliferative stage is represented by P1, P3, and P7, the secretory differentiation stage by P12, P17, and P19, early lactation by L1 and L2, full lactation by L9, and involution by I2. These data have been deposited in NCBI's Gene Expression Omnibus [43] and are accessible through GEO Series accession number GSE8191.

### Microarray data analysis

GeneSpring GX 7.3.1 was used to analyze the data. First, GC-RMA preprocessing was applied to all CEL files. Signal intensity values were normalized as follows. Values below 0.01 were set to 0.01. Each measurement was divided by the 50.0th percentile of all measurements in that sample. Each gene was divided by the median of its measurements in all samples. If the median of the raw values was below 10, then each measurement for that gene was divided by 10 if the numerator was above 10, otherwise the measurement was discarded.

The 12,488 genes were then pre-filtered to remove those genes with unreliable or undetectable signals. Those that did not have an Affymetrix call of 'Present' in at least four of the forty samples were discarded. Additionally, genes with consistently low expressing signal across all ten developmental stages were removed using a minimum threshold of 0.712, empirically decided using GeneSpring's Cross Gene Error Model. Genes that did not exceed this threshold in at least one of the ten developmental stages were removed. The remaining 7,534 genes were used for subsequent analysis.

To find genes that were significantly differentially expressed during different developmental stages of the mammary gland, from initial pregnancy to involution, a one-way ANOVA was applied with a Bonferroni multiple testing correction, *p*-value cutoff of 0.001. No genes would be expected to pass this restriction by chance. 4,832 genes passed. To find genes significantly differentially expressed between individual development stages, all pairwise comparisons were examined with a Tukey post hoc test (*p *< 0.001).

### Pregnancy, lactation, and involution gene sets

The Pregnancy Gene Set, listed in Additional data file 3, is the cluster of genes that are significantly differentially regulated between the start of pregnancy (time point P1) and late pregnancy (time point P19). The Lactation Gene Set, Additional data file 4, is the group of genes significantly differentially regulated between late pregnancy (time point P19) and full lactation (time point L9). The Involution Gene Set, Additional data file 5, refers to those genes significantly differentially regulated between full lactation (time point L9) and involution (time point I2). The Tukey post hoc test was applied to all pairwise comparisons to determine significance (*p *< 0.001).

### Literature Gene Set

The Single Gene Biological Term Mapper feature of CoPub Mapper [44] was used to extract all of the genes co-occuring in PubMed publications with the biological process keyword 'lactation'. In total, 685 genes have been co-published with 'lactation' two or more times. Of these genes, 456 co-occur with a relative score greater than zero. The relative score, previously described [44], is a measure of the frequency of co-occurrence adjusted for the frequency of occurrence of each item individually. This set of 456 genes – those genes known to be involved in lactation – comprise the Literature Gene Set, provided in Additional data file 6.

### Gene ontology analysis

MAPPFinder 2.0 within GenMAPP 2.0 [45] was used to identify gene ontology (GO) terms that are over-represented by significantly regulated genes compared to the full genome-wide set of Affymetrix ProbeIDs given the most recent mouse gene database, Mm-Std_20060628.gdb. Custom Perl and shell scripts were written to format the data for analysis. To determine statistical significance, a Benjamini and Hochberg multiple testing correction was applied to GenMAPP's permuted *p *values with a custom R script using the multtest library [46,47]. In each analysis, statistically enriched GO terms are those terms with 3 or more associated genes, a positive z-score, and a Benjamini and Hochberg adjusted *p*-value less than or equal to 0.05. Thus, a False Discovery Rate of 5% or less would be expected for these enriched GO terms. These same statistical tests were applied to determine enriched GO terms among genes correlated with the principal components and among gene products represented in the lactation network.

### Pathway analysis

Ingenuity Pathways Analysis [48] was used to identify metabolic and signaling pathways that are over-represented by significantly regulated genes compared to all of the genes that are part of the Ingenuity Pathways Knowledge Base. The Ingenuity Pathways Analysis library of canonical pathways includes 80 metabolic and 72 signaling pathways that have been incorporated from various resources and hand-curated. Genes that were statistically up-regulated or down-regulated between the time points studied (i.e. P1 to P19, P19 to Lac9, Lac9 to Inv2) and were associated with a canonical pathway were considered for the analysis. A Fischer's exact test was used to calculate a p-value to determine the probability that the enrichment of the canonical pathway with these genes is explained by chance alone. In this paper, this p-value is referred to as the unadjusted p-value. To improve the stringency of the test, a Benjamini and Hochberg multiple testing correction was applied to the unadjusted *p *values with a custom R script using the multtest library [46,47]. Unless otherwise stated, pathways reported to be statistically significant are those with a Benjamini and Hochberg adjusted *p*-value less than or equal to 0.05. These same statistical tests were applied to determine enriched pathways among genes correlated with the principal components and among gene products represented in the lactation network.

### Protein interaction network analysis

Gene lists were converted to Human Swissprot IDs using tables from the Ensembl database, release 41 [21]. For each list of Human Swissprot IDs, interactions between those gene products were obtained from OPHID [19] and post-processed using custom scripts to determine all of the linkages in the network and to generate a network file. This network file was then explored using Pajek 1.16 [49], a program for large network analysis.

## List of abbreviations

GO, gene ontology; 

I2, involution day 2;  

L2, lactation day 2; 

L9, lactation day 9;  

MECs, mammary epithelial cells; 

OPHID, Online Predicted Human Interaction Database; 

P1, pregnancy day 1; 

P12, pregnancy day 12;

P17, pregnancy day 17;

P19, pregnancy day 19.

## Authors' contributions

DL conceived of the study, wrote the custom scripts, performed all analyses, interpreted results, and drafted the manuscript. MN provided microarray data, interpreted results, and helped draft the manuscript. MR provided microarray data and reviewed the manuscript. KP supervised the statistical analyses, wrote the primary R script, and contributed to the manuscript. JBG participated in the coordination of the study and contributed to the manuscript. All authors read and approved the final manuscript.

## Supplementary Material

Additional file 1 Additional data file 1 is an Excel spreadsheet that lists genes with a standard correlation of 0.90 or better with the first principal component. Click here for file

Additional file 2 Additional data file 2 is an Excel spreadsheet that lists genes that are suppressed during lactation. These genes are down-regulated by L2 relative to P17 and up-regulated by I2 relative to L9. Click here for file

Additional file 5 Additional data file 5 is an Excel file that lists genes that are members of the Involution Gene Set. These genes are differentially regulated between time points L9 and I2. Two spreadsheets are provided in the file, one for up-regulated and one for down-regulated genes. Click here for file

Additional file 7 Additional data file 7 is an Excel file of genes that are associated with the GO terms in Table 2 and are members of the Pregnancy Gene Set (i.e. differentially regulated between time points P1 and P19). Two spreadsheets are provided in the file, one for up-regulated and one for down-regulated genes. Click here for file

Additional file 8 Additional data file 8 is an Excel file of genes that are associated with the GO terms in Table 3 and are members of the Lactation Gene Set (i.e. differentially regulated between time points P19 and L9). Two spreadsheets are provided in the file, one for up-regulated and one for down-regulated genes. Click here for file

Additional file 9 Additional data file 9 is an Excel file of genes that are associated with the GO terms in Table 4 and are members of the Involution Gene Set (i.e. differentially regulated between time points L9 and I2). Two spreadsheets are provided in the file, one for up-regulated and one for down-regulated genes. Click here for file

Additional file 3 Additional data file 3 is an Excel file that lists genes that are members of the Pregnancy Gene Set. These genes are differentially regulated between time points P1 and P19. Two spreadsheets are provided in the file, one for up-regulated and one for down-regulated genes. Click here for file

Additional file 4 Additional data file 4 is an Excel file that lists genes that are members of the Lactation Gene Set. These genes are differentially regulated between time points P19 and L9. Two spreadsheets are provided in the file, one for up-regulated and one for down-regulated genes. Click here for file

Additional file 19 Additional data file 19 is an Excel file that provides the adjusted and unadjusted p-values and associated genes for all pathways which contain one or more differentially regulated genes. The file contains three spreadsheets, one each for the Pregnancy, Lactation, and Involution Gene Sets. Click here for file

Additional file 20 Additional data file 20 is a Word document that contains pathway diagrams for the significant pathways associated with the Pregnancy Gene Set. Click here for file

Additional file 21 Additional data file 21 is a Word document that contains pathway diagrams for the significant pathways associated with the Lactation Gene Set. Click here for file

Additional file 22 Additional data file 22 is a Word document that contains pathway diagrams for the significant pathways associated with the Involution Gene Set. Click here for file

Additional file 10 Additional data file 10 is an Excel file that provides HGNC symbols, Swissprot identifiers, and descriptions for each protein interaction pair associated with Figure 8. Six spreadsheets are provided in the file, one for each of the networks in Figure 8. Click here for file

Additional file 11 Additional data file 11, an SWF movie file, is a 3D animation of the network in Figure 8A (the within-set pregnancy network) in which the nodes are labeled with HGNC symbols. The HGNC symbols, Swissprot identifiers, and descriptions for each protein interaction pair are given in Additional file 10. Click here for file

Additional file 12 Additional data file 12, an SWF movie file, is a 3D animation of the trivial network in Figure 8B (the within-set lactation network) in which the nodes are labeled with HGNC symbols. The HGNC symbols, Swissprot identifiers, and descriptions for each protein interaction pair are given in Additional file 10. Click here for file

Additional file 13 Additional data file 13, an SWF movie file, is a 3D animation of the network in Figure 8C (the within-set involution network) in which the nodes are labeled with HGNC symbols. The HGNC symbols, Swissprot identifiers, and descriptions for each protein interaction pair are given in Additional file 10. Click here for file

Additional file 14 Additional data file 14, an SWF movie file, is a 3D animation of the network in Figure 8D (the pregnancy network) in which the nodes are labeled with HGNC symbols. The HGNC symbols, Swissprot identifiers, and descriptions for each protein interaction pair are given in Additional file 10. Click here for file

Additional file 15 Additional data file 15, an SWF movie file, is a 3D animation of the network in Figure 8E (the lactation network) in which the nodes are labeled with HGNC symbols. The HGNC symbols, Swissprot identifiers, and descriptions for each protein interaction pair are given in Additional file 10. Click here for file

Additional file 16 Additional data file 16, an SWF movie file, is a 3D animation of the network in Figure 8F (the involution network) in which the nodes are labeled with HGNC symbols. The HGNC symbols, Swissprot identifiers, and descriptions for each protein interaction pair are given in Additional file 10. Click here for file

Additional file 17 Additional data file 17 is an Excel file that provides HGNC symbols, Swissprot identifiers, and descriptions for each protein interaction pair that comprises the comprehensive lactation network in Figure 9. Click here for file

Additional file 18 Additional data file 18, an SWF movie file, is a 3D animation of the network in Figure 9 (comprehensive lactation network) in which the nodes are labeled with HGNC symbols. The HGNC symbols, Swissprot identifiers, and descriptions for each protein interaction pair are given in Additional file 17. Click here for file

Additional file 23 Additional data file 23 is a Word document that contains manually curated general and mammary-specific functions of the major hubs of the comprehensive lactation network in Figure 9. Click here for file

Additional file 6 Additional data file 6 is an Excel file that lists genes that are members of the Literature Gene Set. These genes have been co-published with the keyword 'lactation'. Click here for file
