# Supplementary material for: Gene regulatory networks in lactation: identification of global principles using bioinformatics
Source: BMC Syst Biol. 2007 Nov 27;1:56. doi: 10.1186/1752-0509-1-56 (PMC2225983; doi:10.1186/1752-0509-1-56)

**Transcriptionally Regulated Pathways During Pregnancy**

All pathways in this document are marginally significantly (unadjusted p < 0.05) enriched with genes from the Pregnancy Gene Set. No pathways are significant after a Benjamini and Hochberg multiple testing correction. See additional data file 19 for the complete list of pathways and associated molecules from the Pregnancy Gene Set.

| Pathway | B&H adjusted p-value | unadjusted p-value |
| --- | --- | --- |
| PI3K/AKT Signaling | 0.1368 | 0.0038 |
| Wnt/β-catenin Signaling | 0.1368 | 0.0038 |
| LPS/IL-1 Mediated Inhibition of RXR Function | 0.1368 | 0.0045 |
| Complement and Coagulation Cascades | 0.1368 | 0.005 |
| Axonal Guidance Signaling | 0.1368 | 0.0054 |
| Leukocyte Extravasation Signaling | 0.1368 | 0.0055 |
| Integrin Signaling | 0.1368 | 0.0063 |
| Ephrin Receptor Signaling | 0.1444 | 0.0076 |
| Huntington's Disease Signaling | 0.146933333 | 0.0091 |
| NRF2-mediated Oxidative Stress Response | 0.146933333 | 0.0098 |
| Apoptosis Signaling | 0.146933333 | 0.0114 |
| ERK/MAPK Signaling | 0.146933333 | 0.0116 |
| Amyloid Processing | 0.3192 | 0.0273 |
| NF-κB Signaling | 0.417493333 | 0.0404 |
| IGF-1 Signaling | 0.417493333 | 0.0412 |
| Xenobiotic Metabolism Signaling | 0.4636 | 0.0488 |


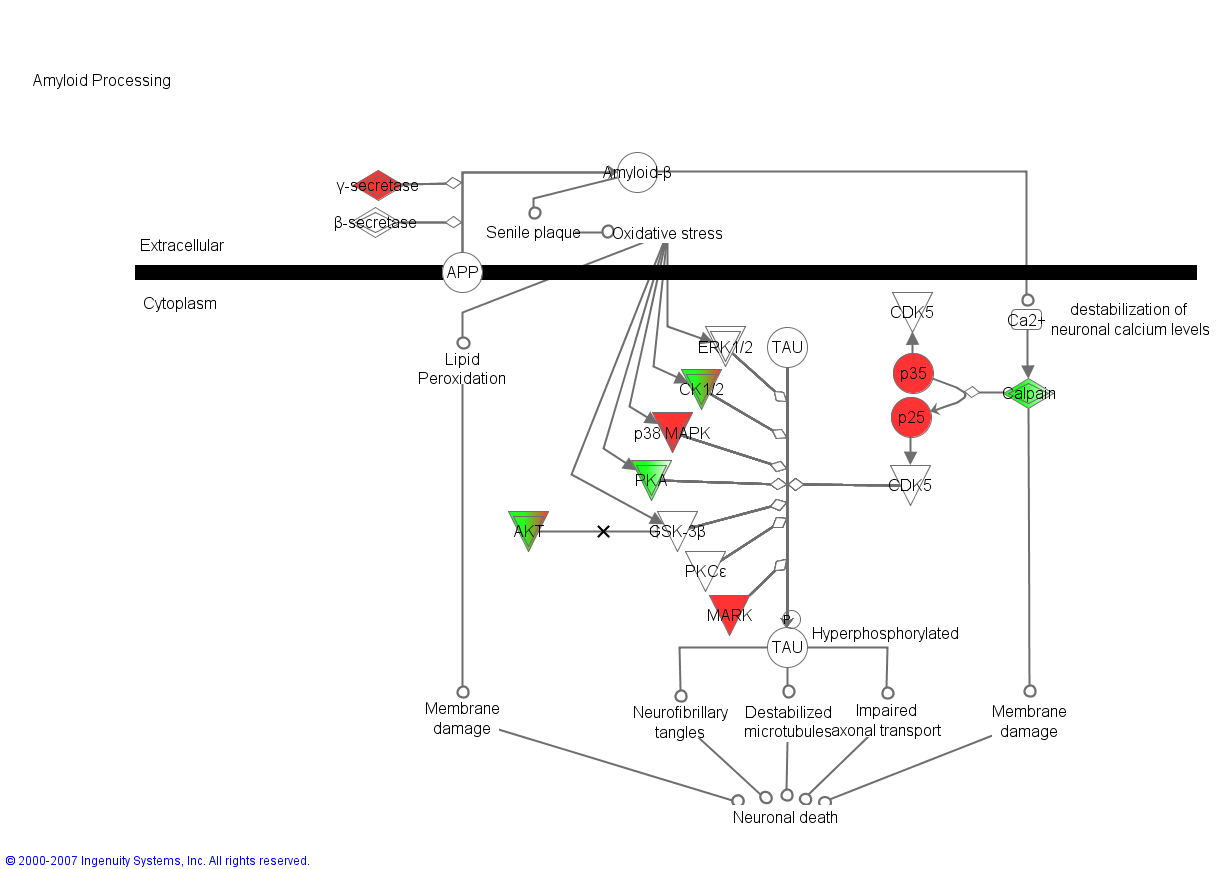


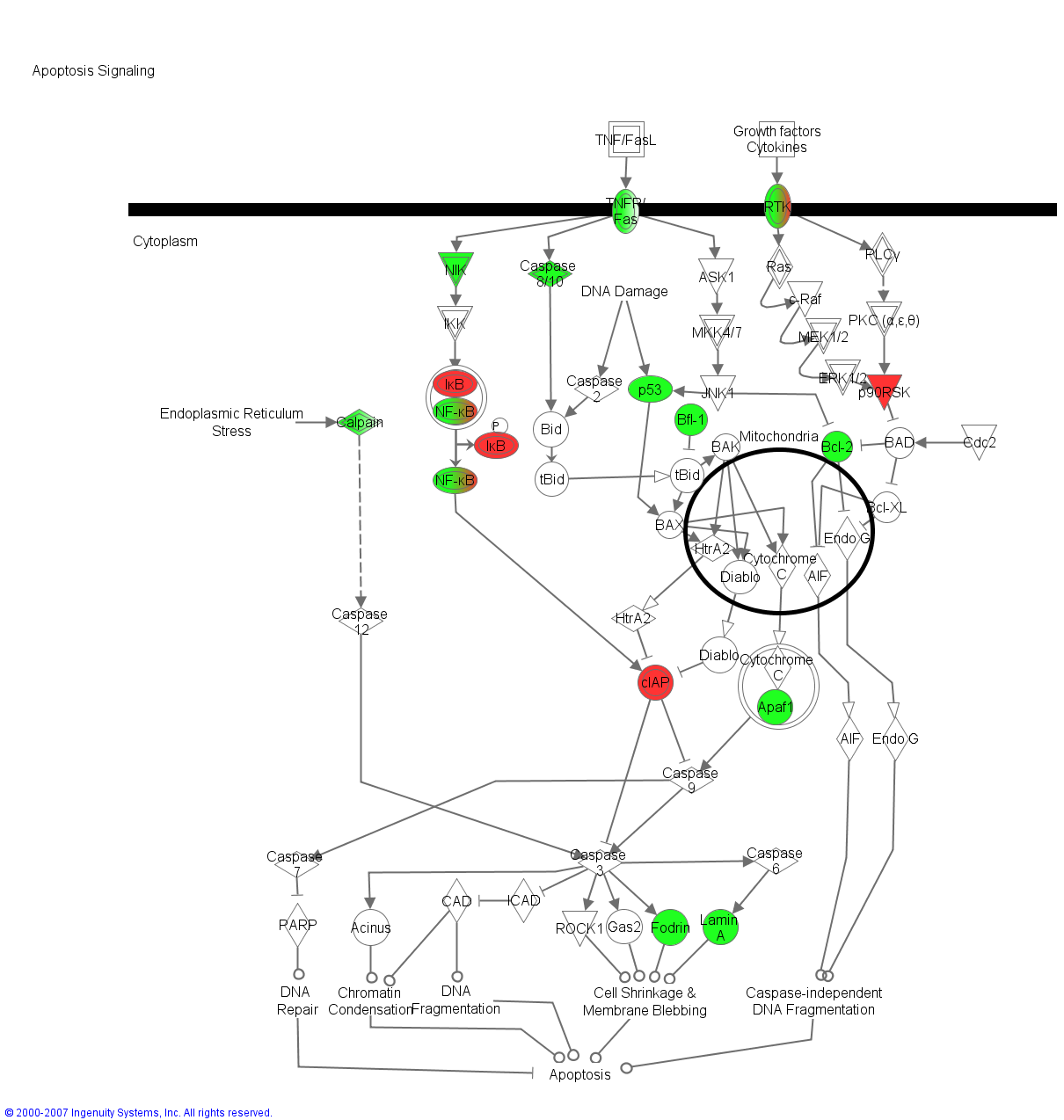


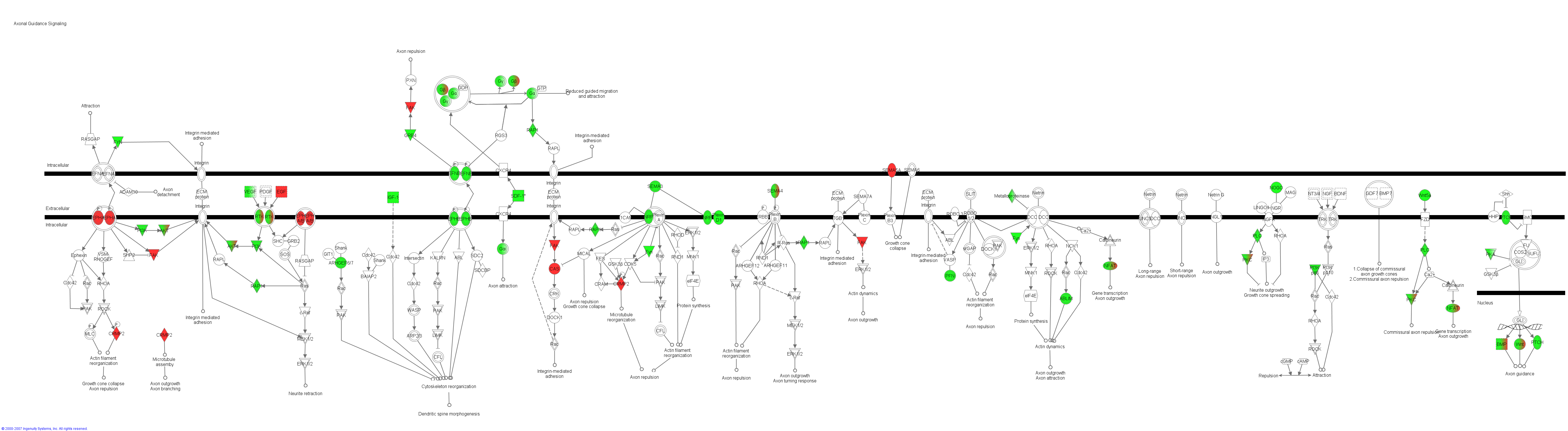


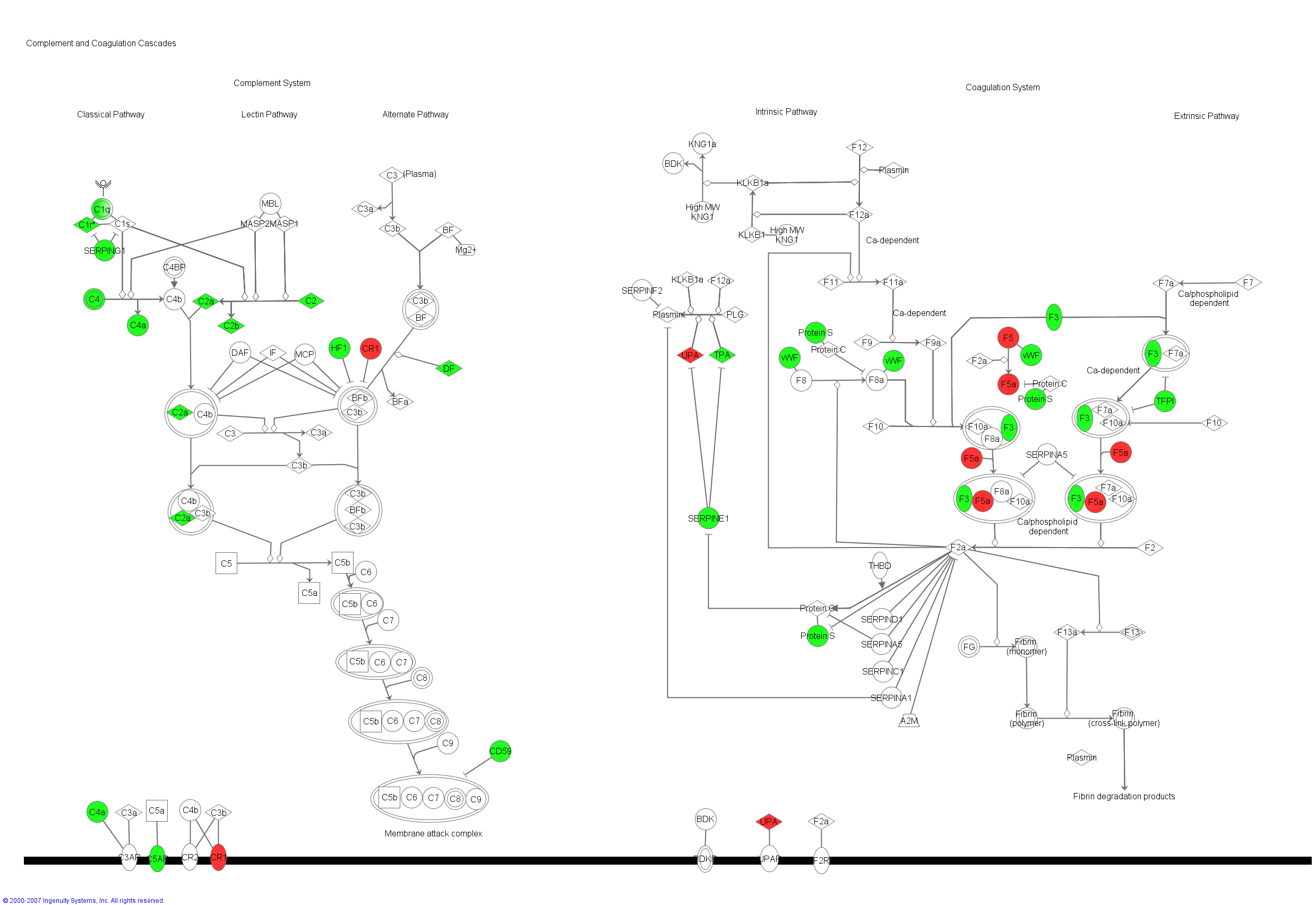


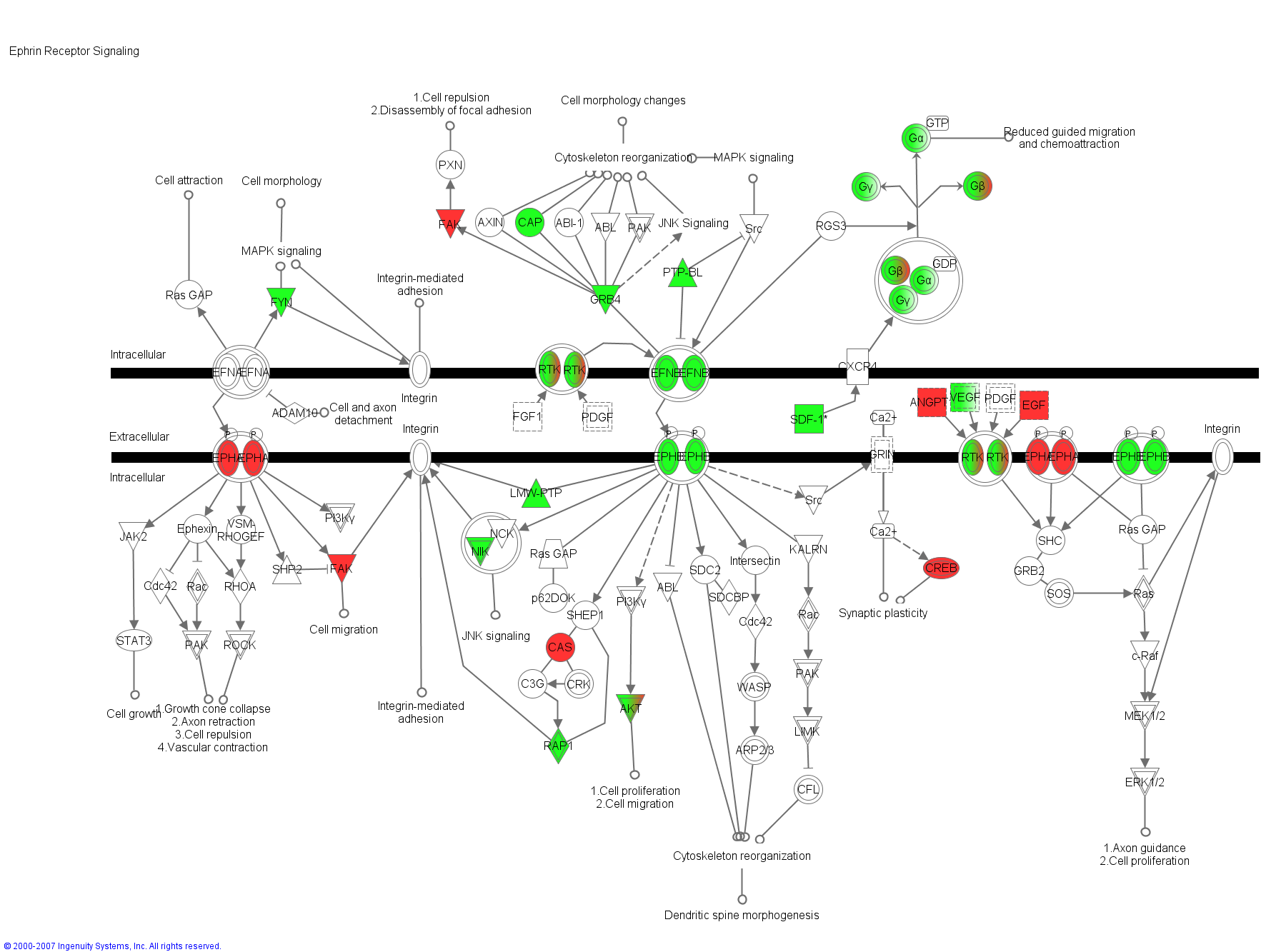


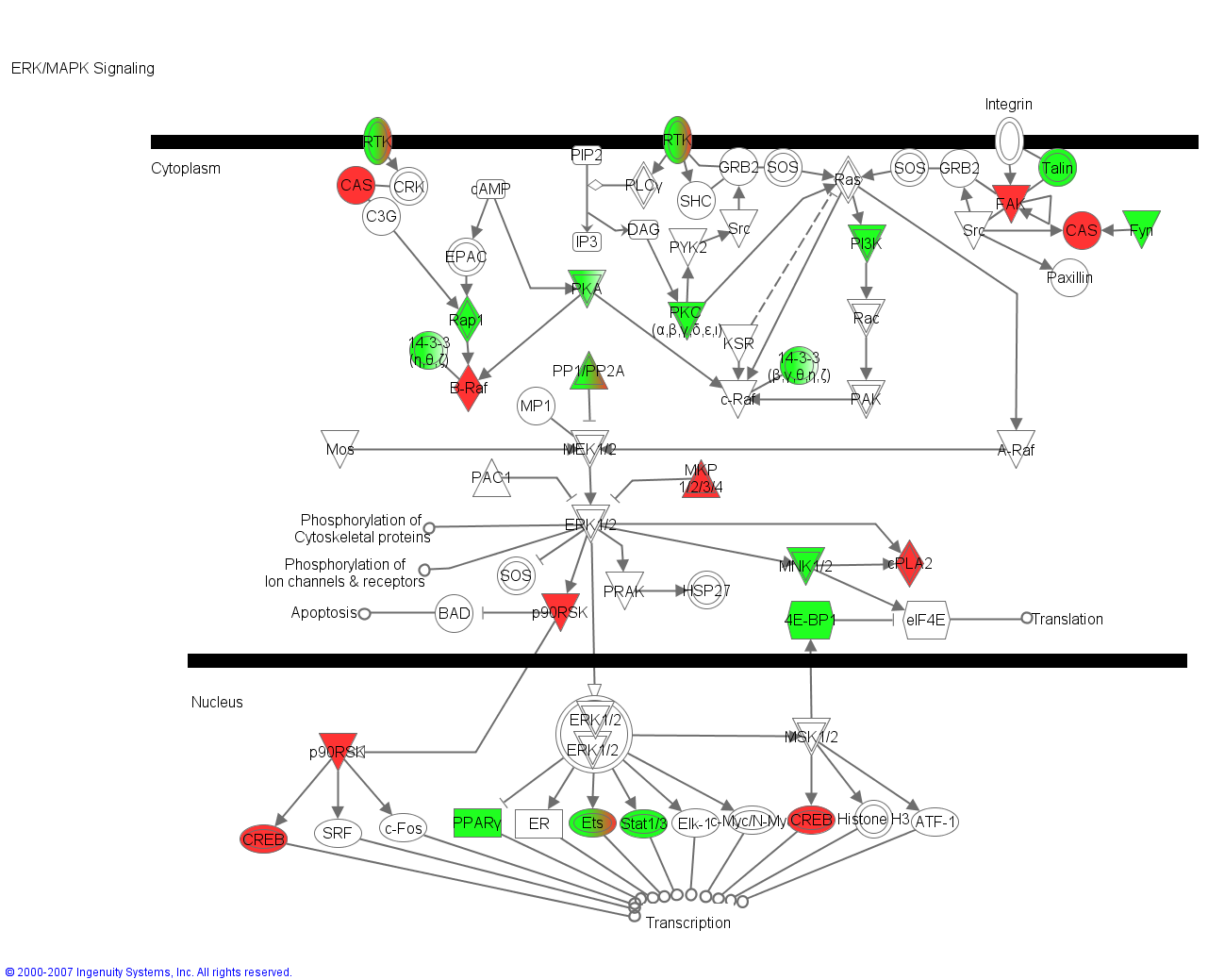


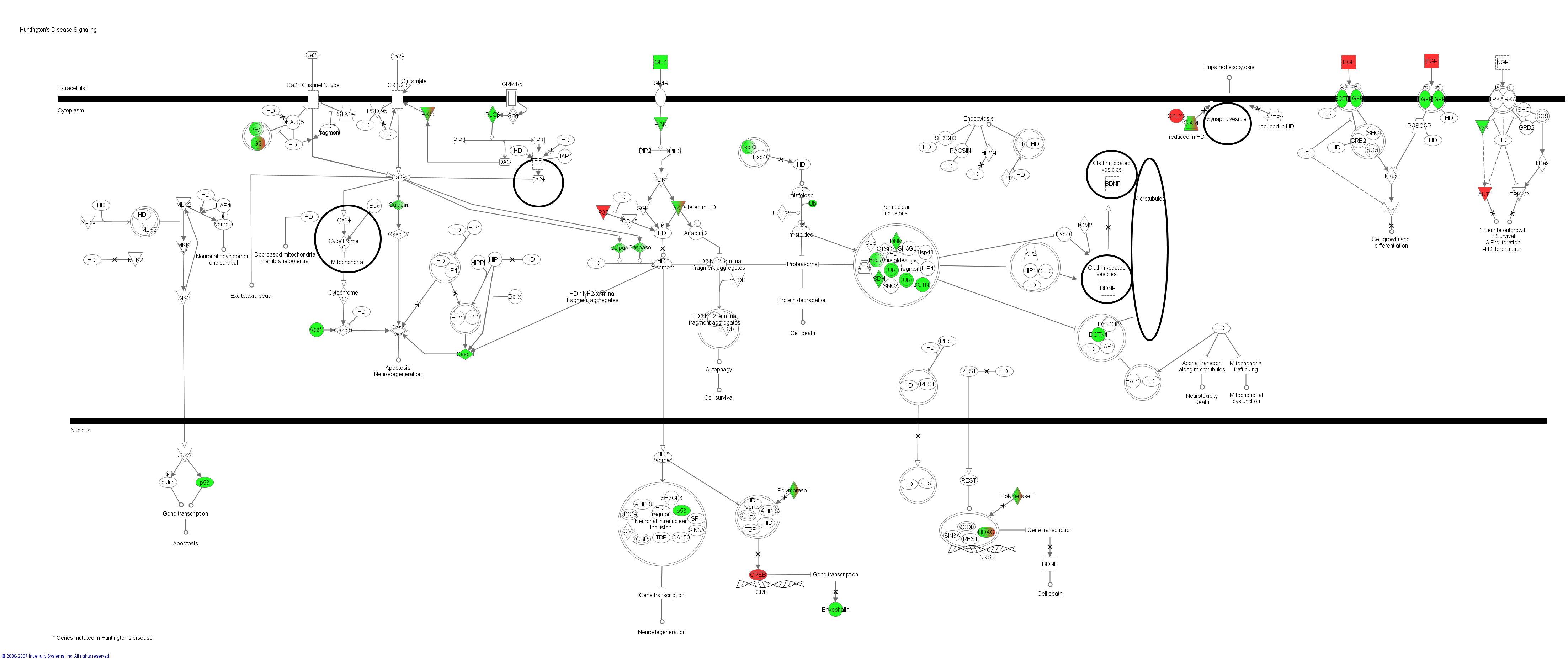


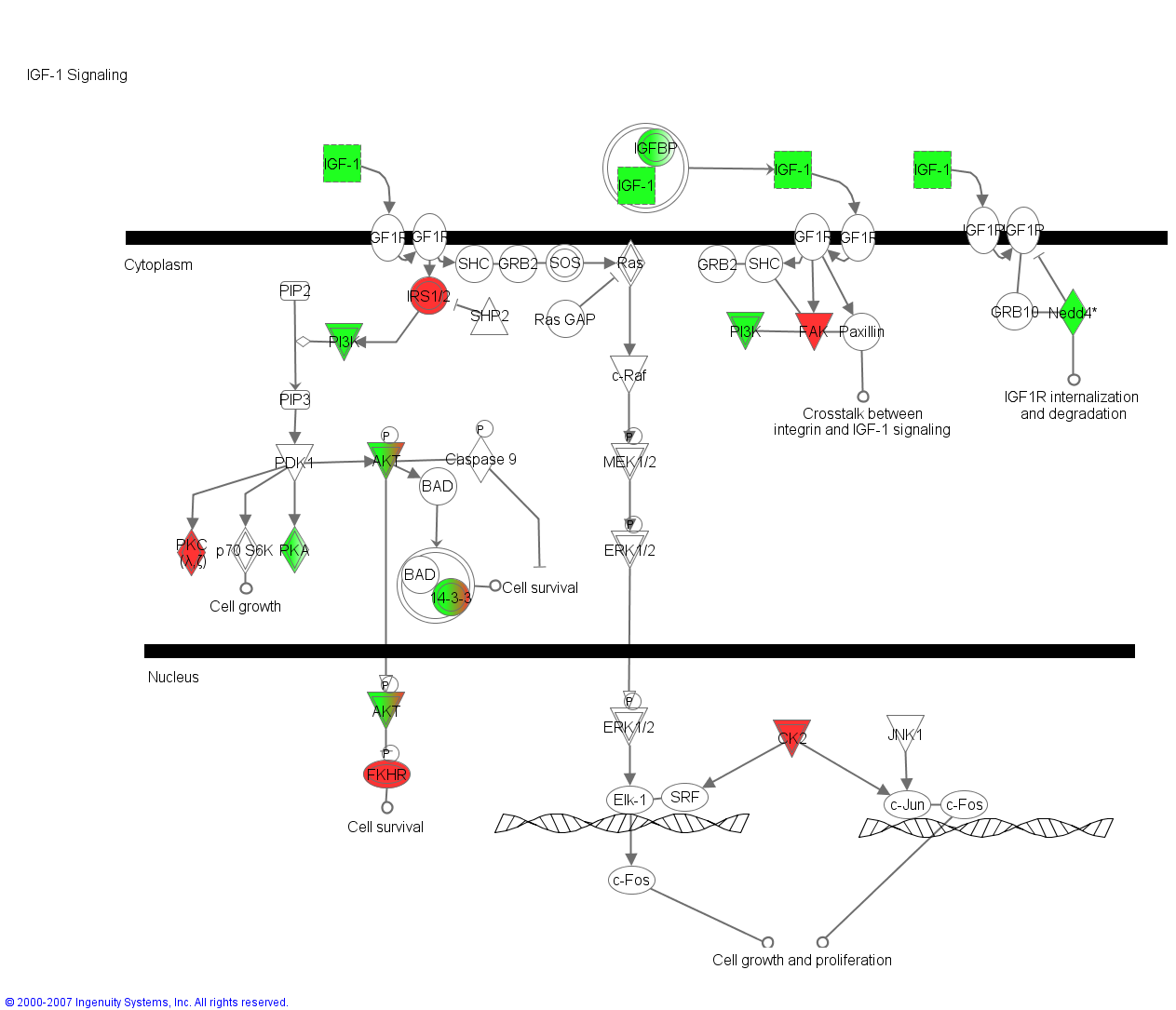


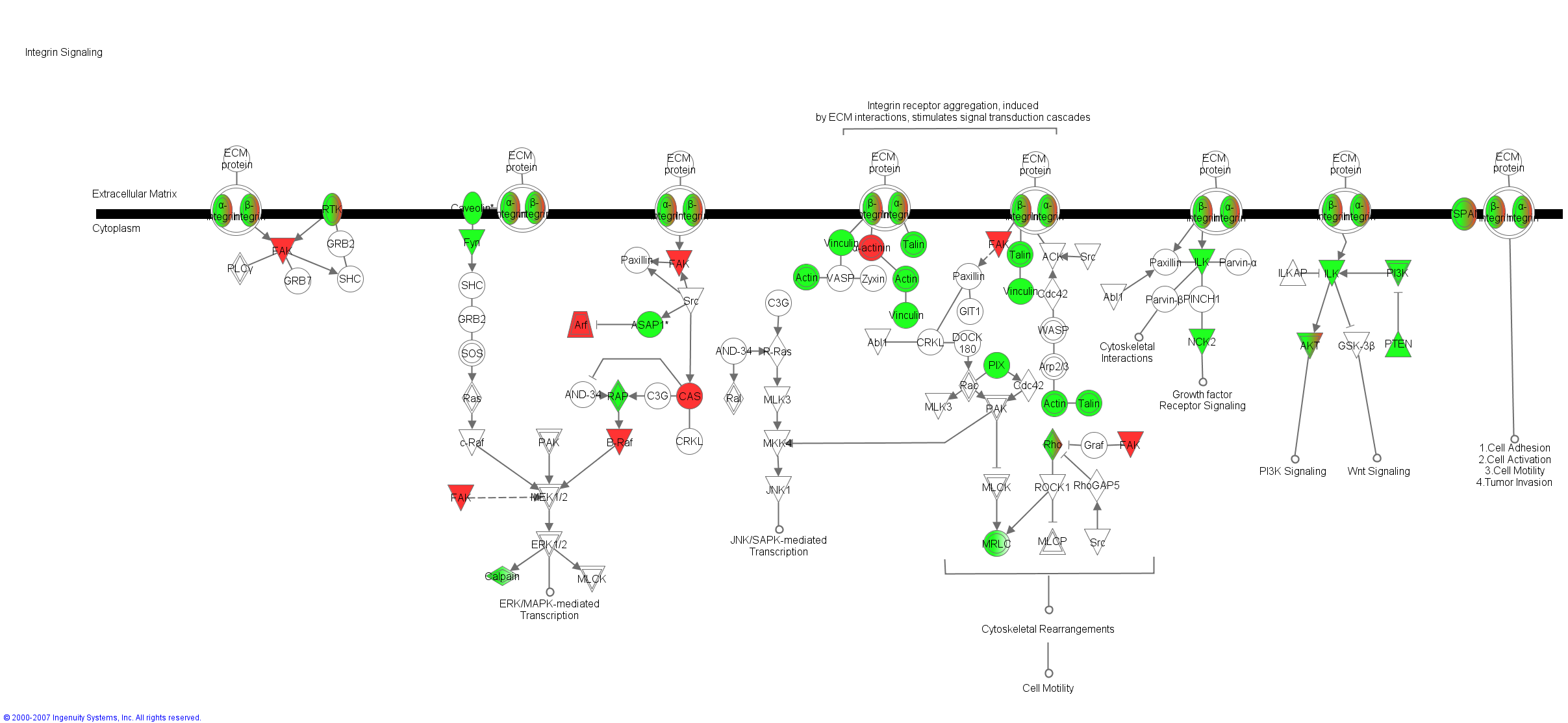


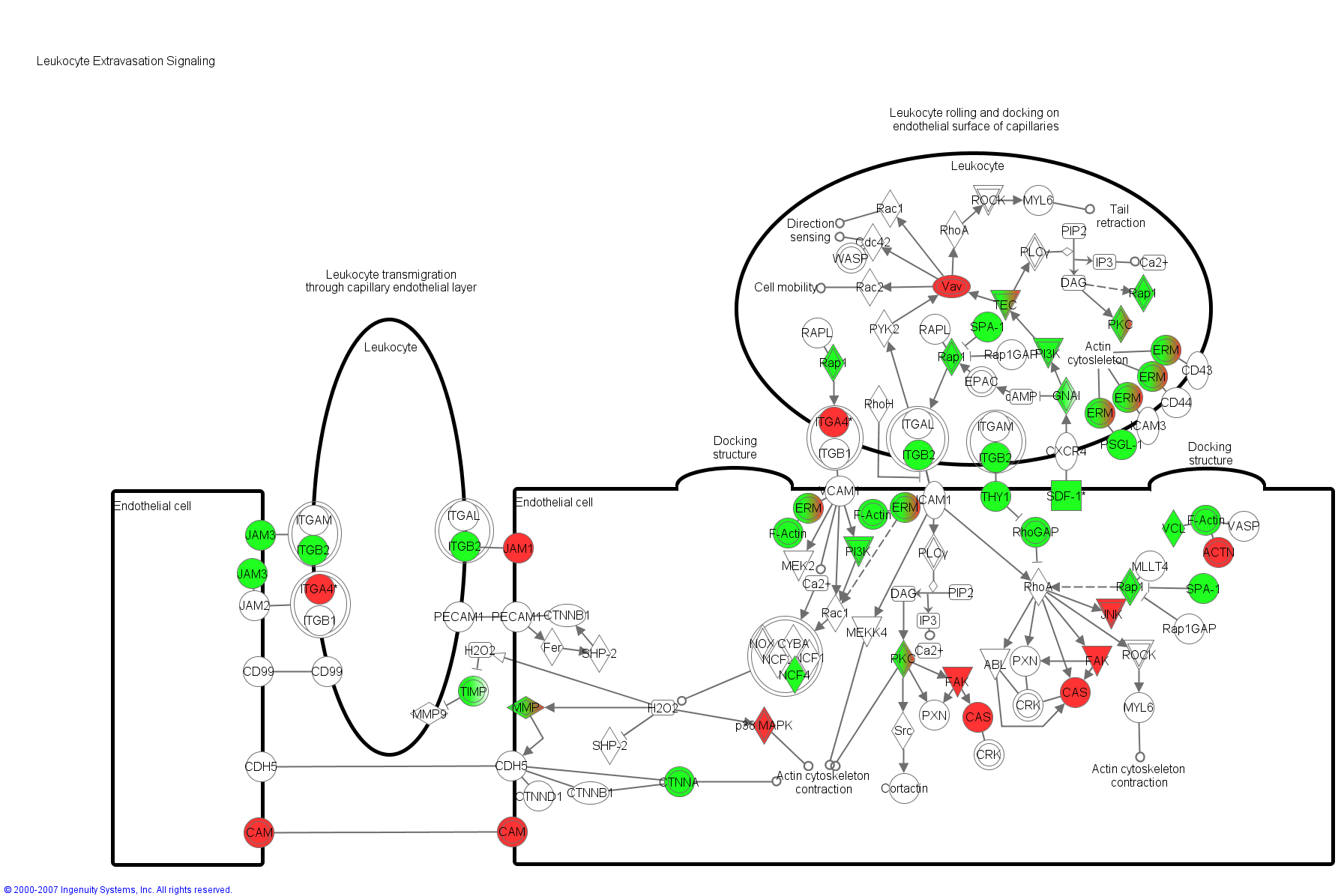


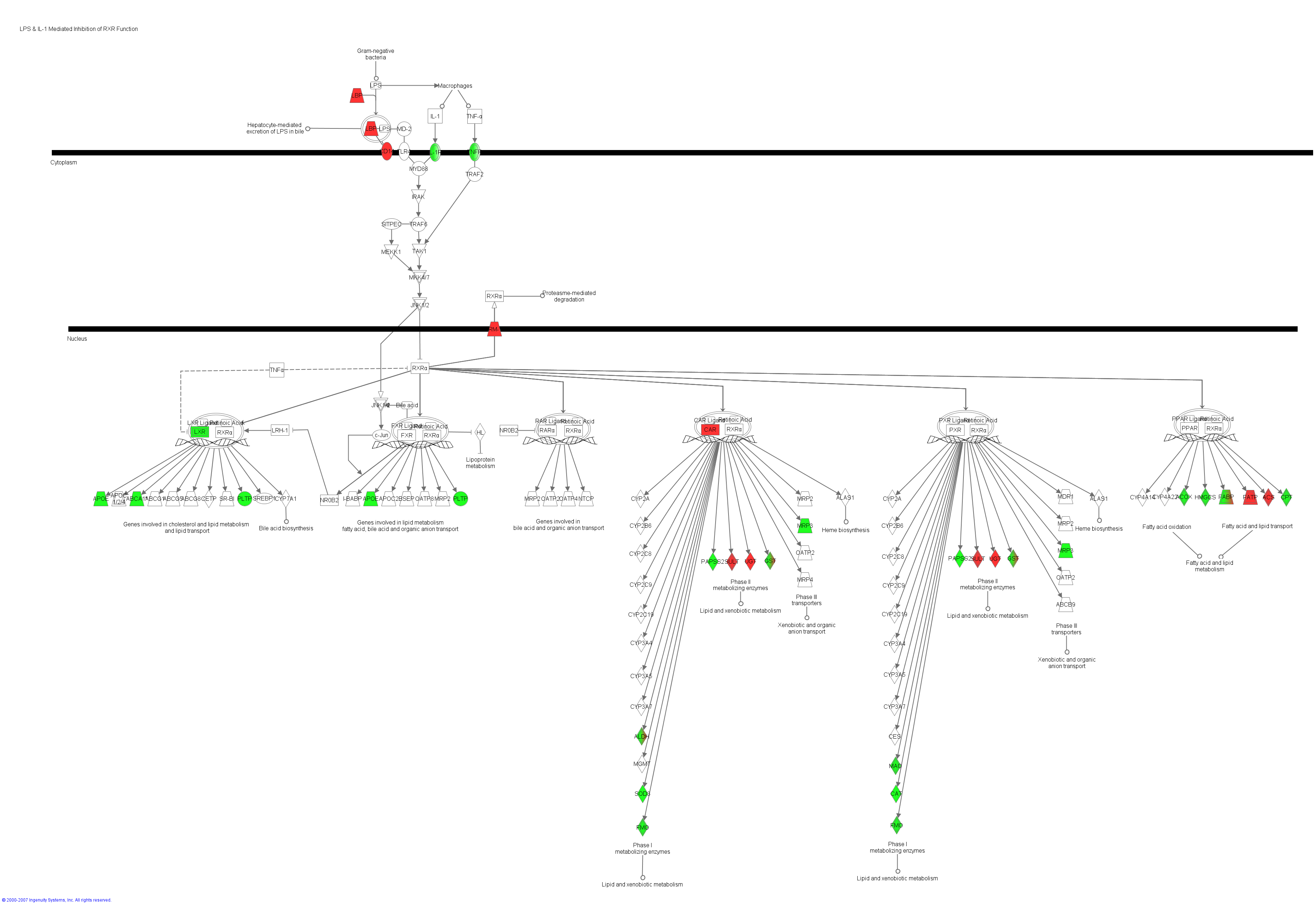


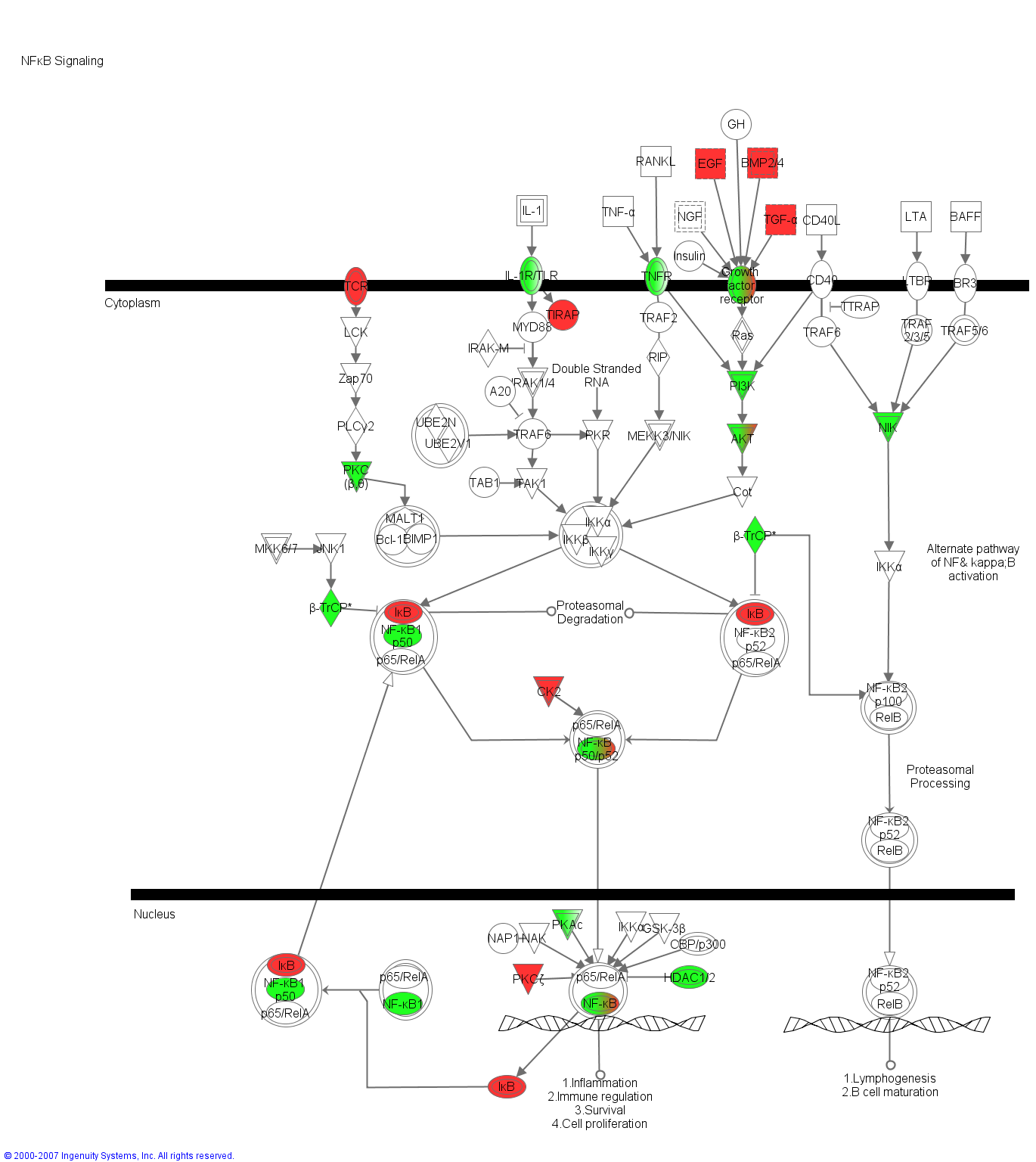


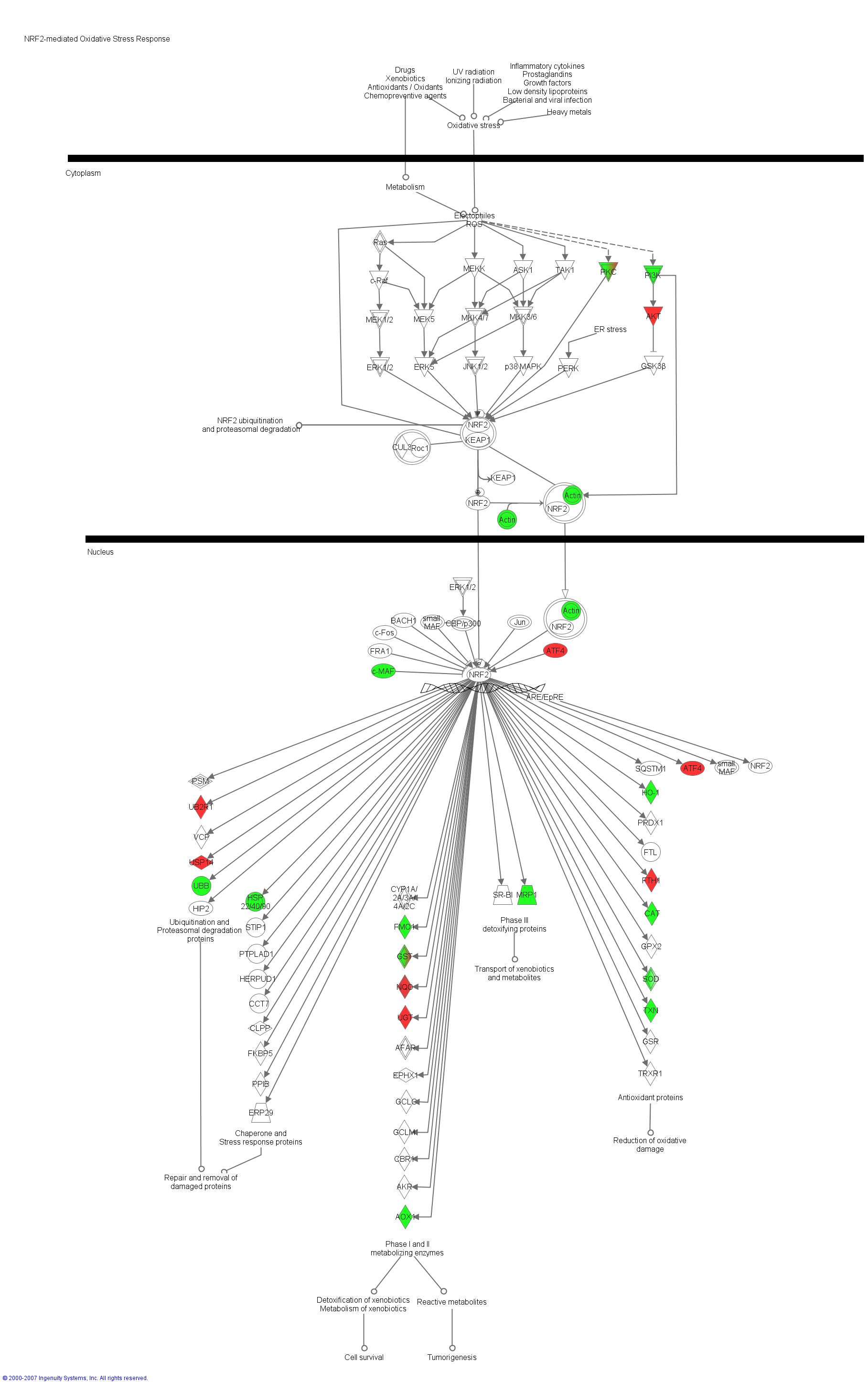


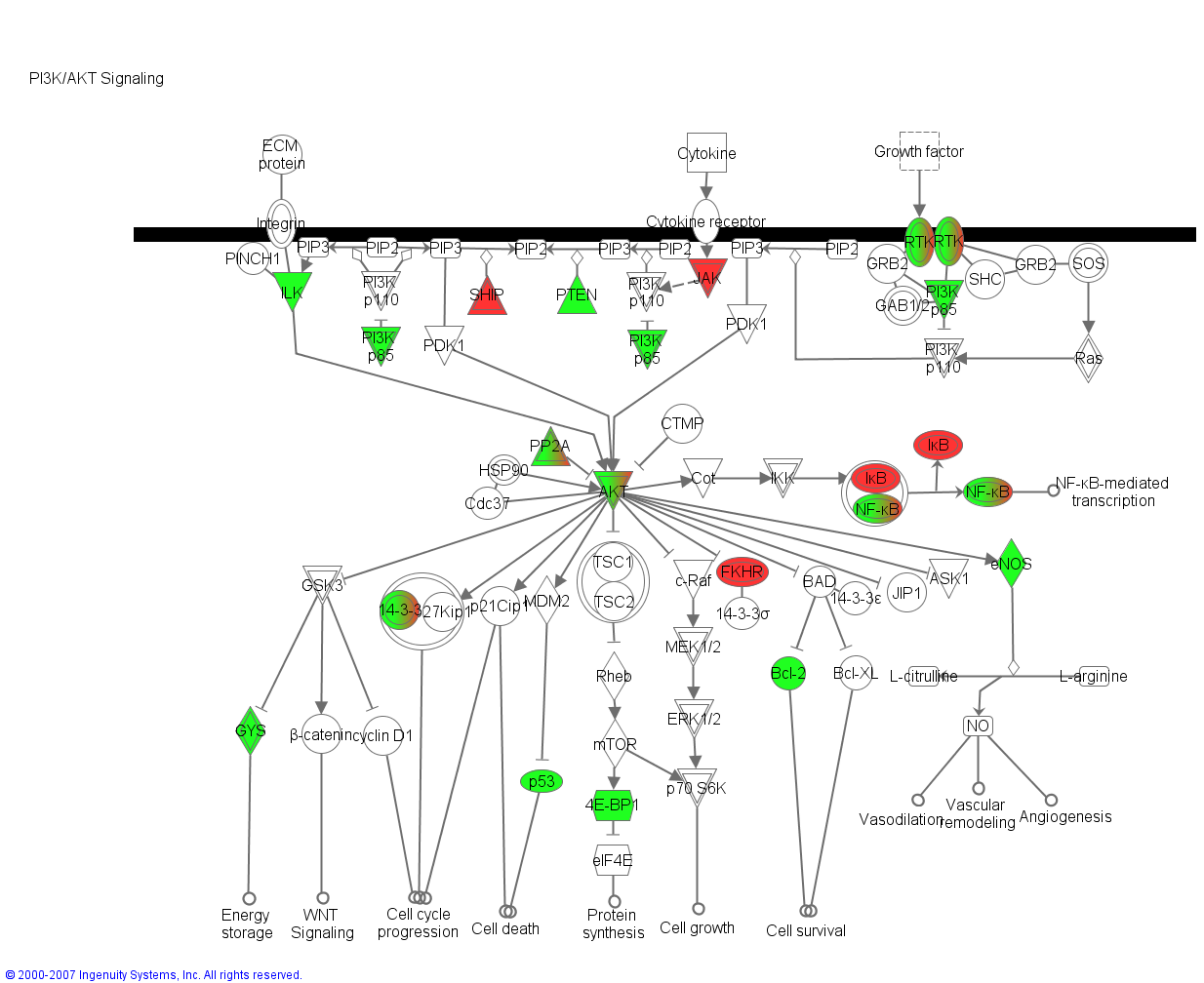


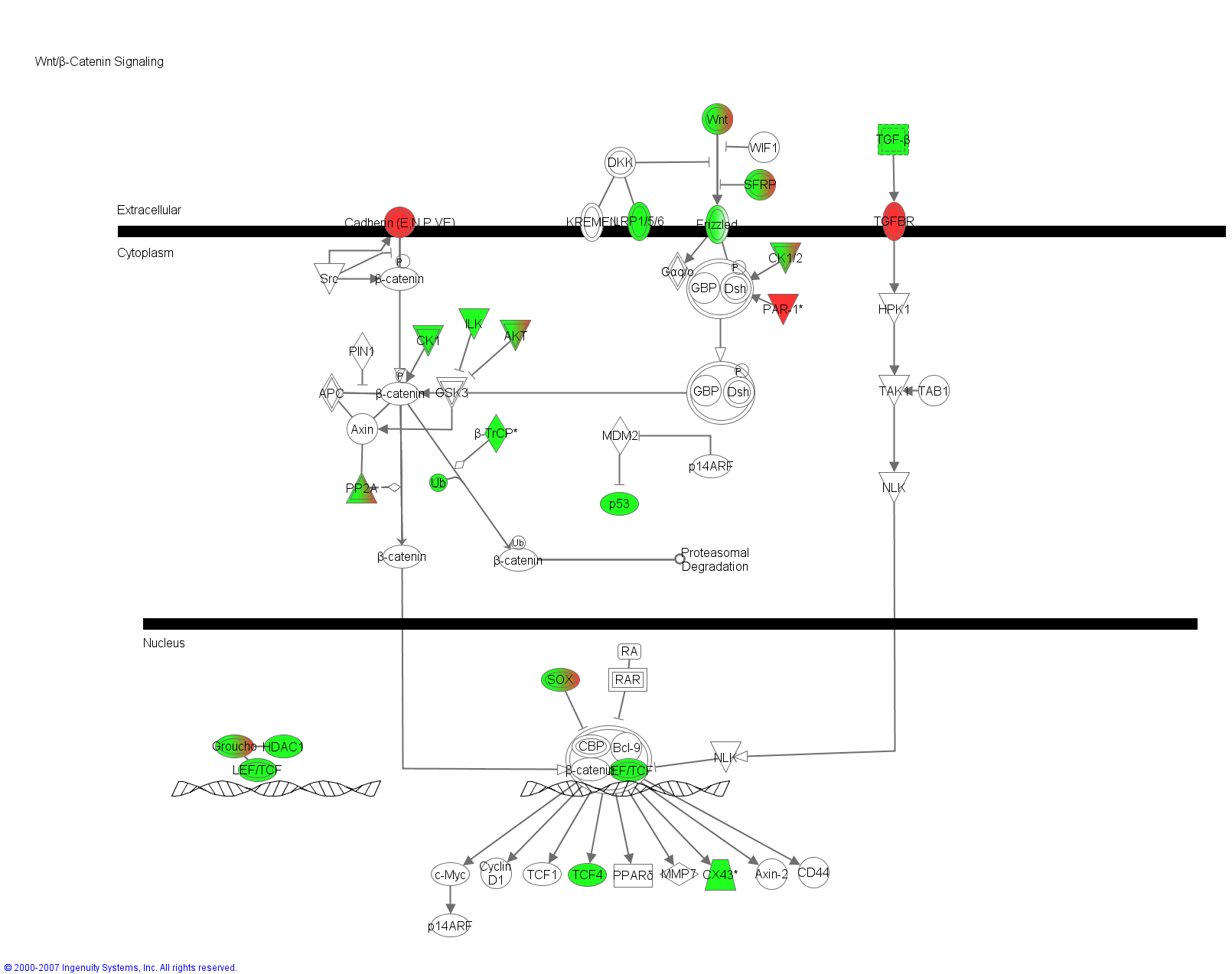


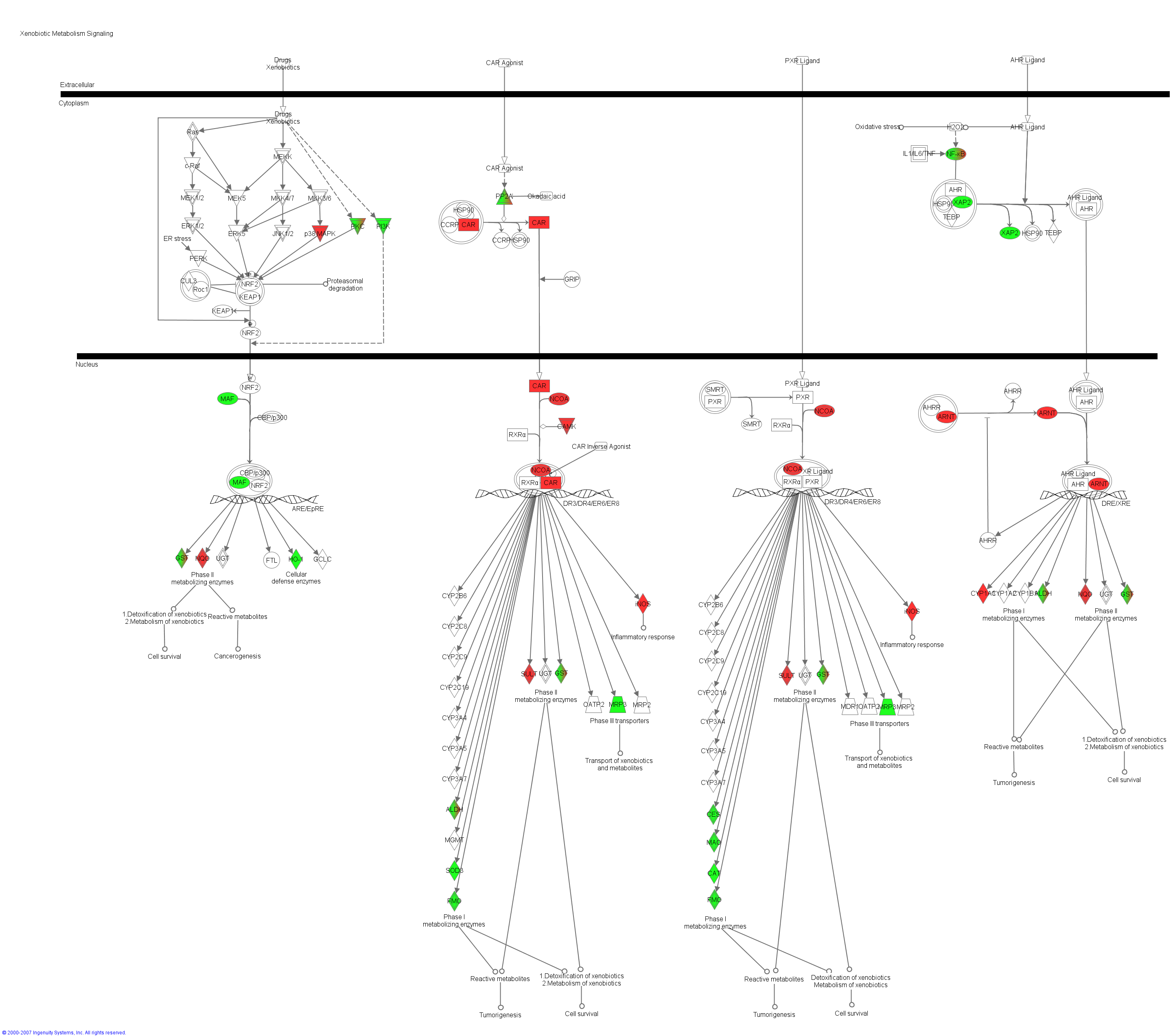

Supplement: Additional file 20 — Additional data file 20 is a Word document that contains pathway diagrams for the significant pathways associated with the Pregnancy Gene Set. [file 1752-0509-1-56-S20.doc]
