# Supplementary material for: Gene regulatory networks in lactation: identification of global principles using bioinformatics
Source: BMC Syst Biol. 2007 Nov 27;1:56. doi: 10.1186/1752-0509-1-56 (PMC2225983; doi:10.1186/1752-0509-1-56)

**Transcriptionally Regulated Pathways During Lactation**

All pathways in this document are marginally significantly (unadjusted p < 0.05) enriched with genes from the Lactation Gene Set. Highlighted pathways are significant after a Benjamini and Hochberg multiple testing correction. See additional data file 19 for the complete list of pathways and associated molecules from the Lactation Gene Set.

| Pathway | B&H adjusted p-value | unadjusted p-value |
| --- | --- | --- |
| Protein Ubiquitination Pathway | 0 | 0.0000 |
| Integrin Signaling | 0 | 0.0000 |
| PI3K/AKT Signaling | 0.010133333 | 0.0002 |
| Oxidative Phosphorylation | 0.0266 | 0.0007 |
| Actin Cytoskeleton Signaling | 0.028228571 | 0.0010 |
| Ephrin Receptor Signaling | 0.028228571 | 0.0013 |
| PPARα/RXRα Activation | 0.028228571 | 0.0013 |
| NRF2-mediated Oxidative Stress Response | 0.0437 | 0.0023 |
| Antigen Presentation Pathway | 0.064177778 | 0.0038 |
| Hypoxia Signaling in the Cardiovascular System | 0.07296 | 0.0048 |
| Amyloid Processing | 0.169963636 | 0.0123 |
| VEGF Signaling | 0.173533333 | 0.0137 |
| PPAR Signaling | 0.22727619 | 0.0213 |
| Xenobiotic Metabolism Signaling | 0.22727619 | 0.0224 |
| ERK/MAPK Signaling | 0.22727619 | 0.0226 |
| Leukocyte Extravasation Signaling | 0.22727619 | 0.0257 |
| Chemokine Signaling | 0.22727619 | 0.0296 |
| PTEN Signaling | 0.22727619 | 0.0305 |
| Huntington's Disease Signaling | 0.22727619 | 0.0311 |
| B Cell Receptor Signaling | 0.22727619 | 0.0311 |
| IL-6 Signaling | 0.22727619 | 0.0314 |
| Apoptosis Signaling | 0.250109091 | 0.0362 |


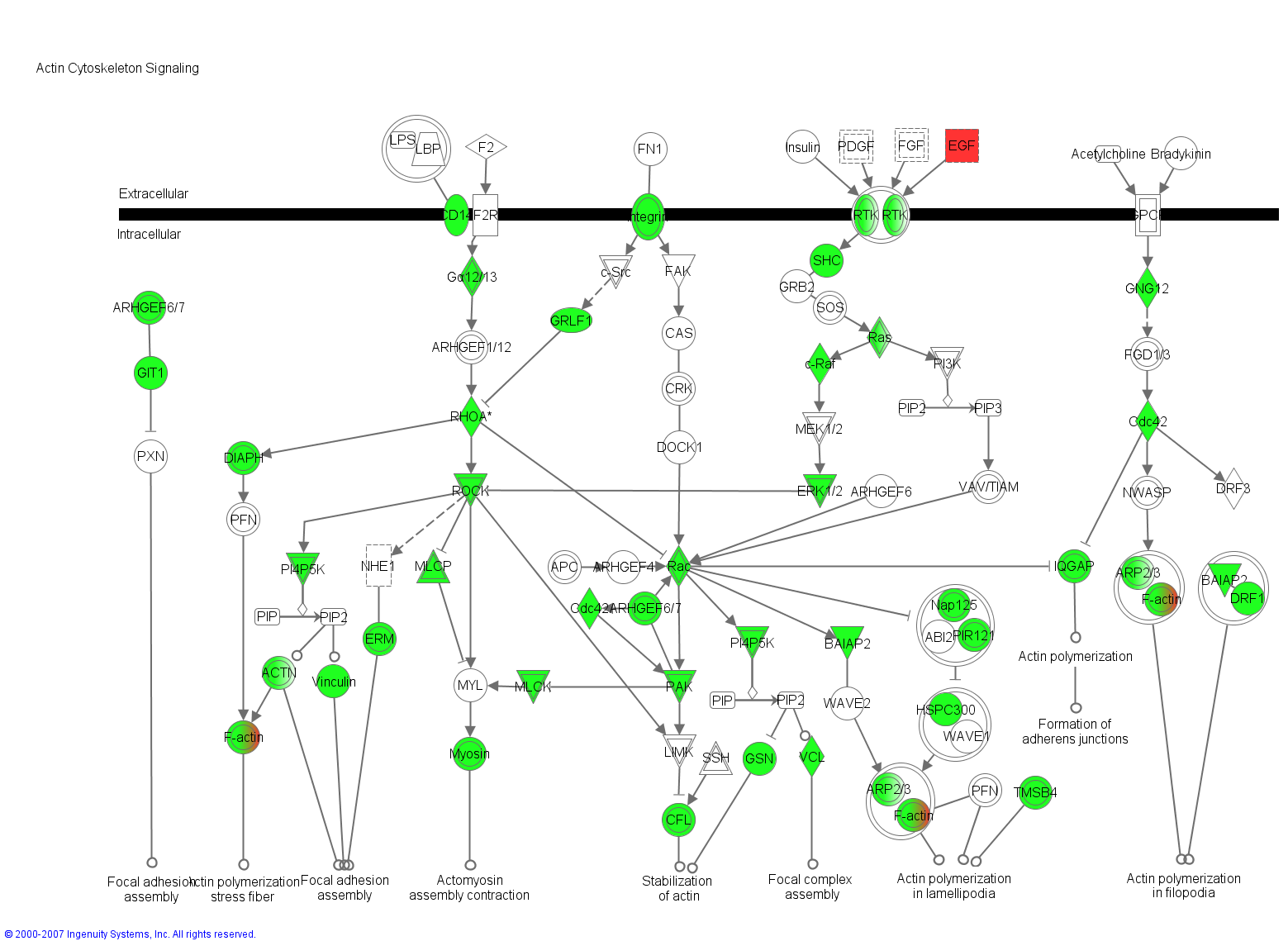


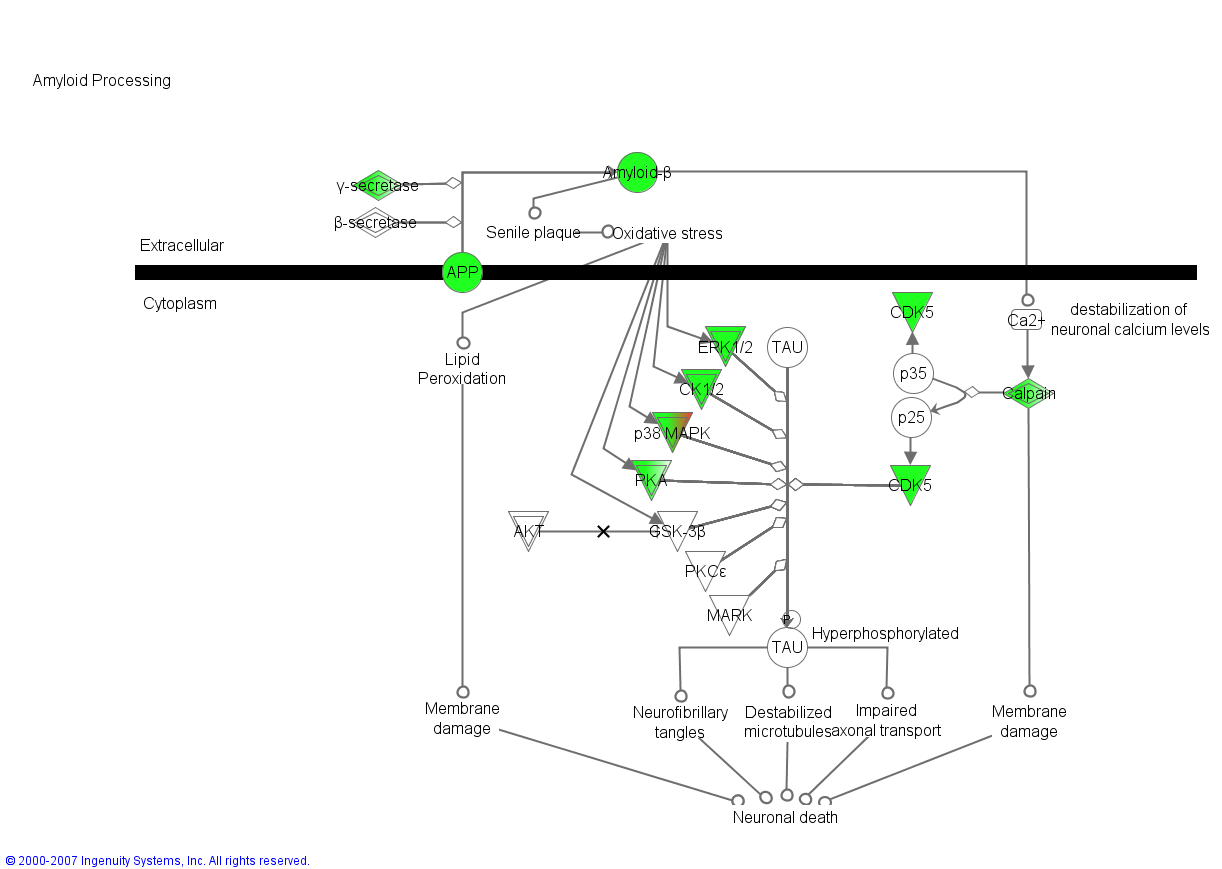


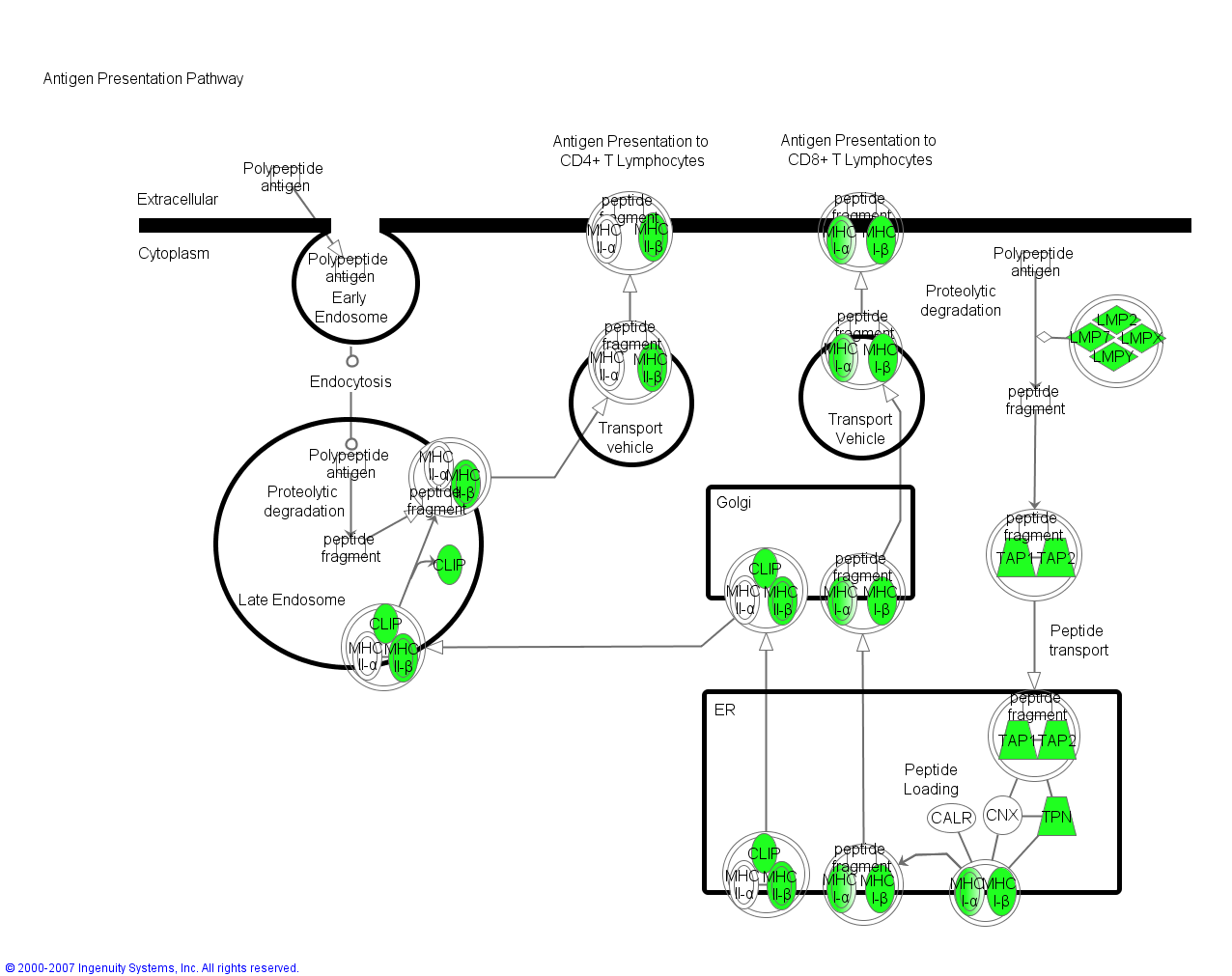


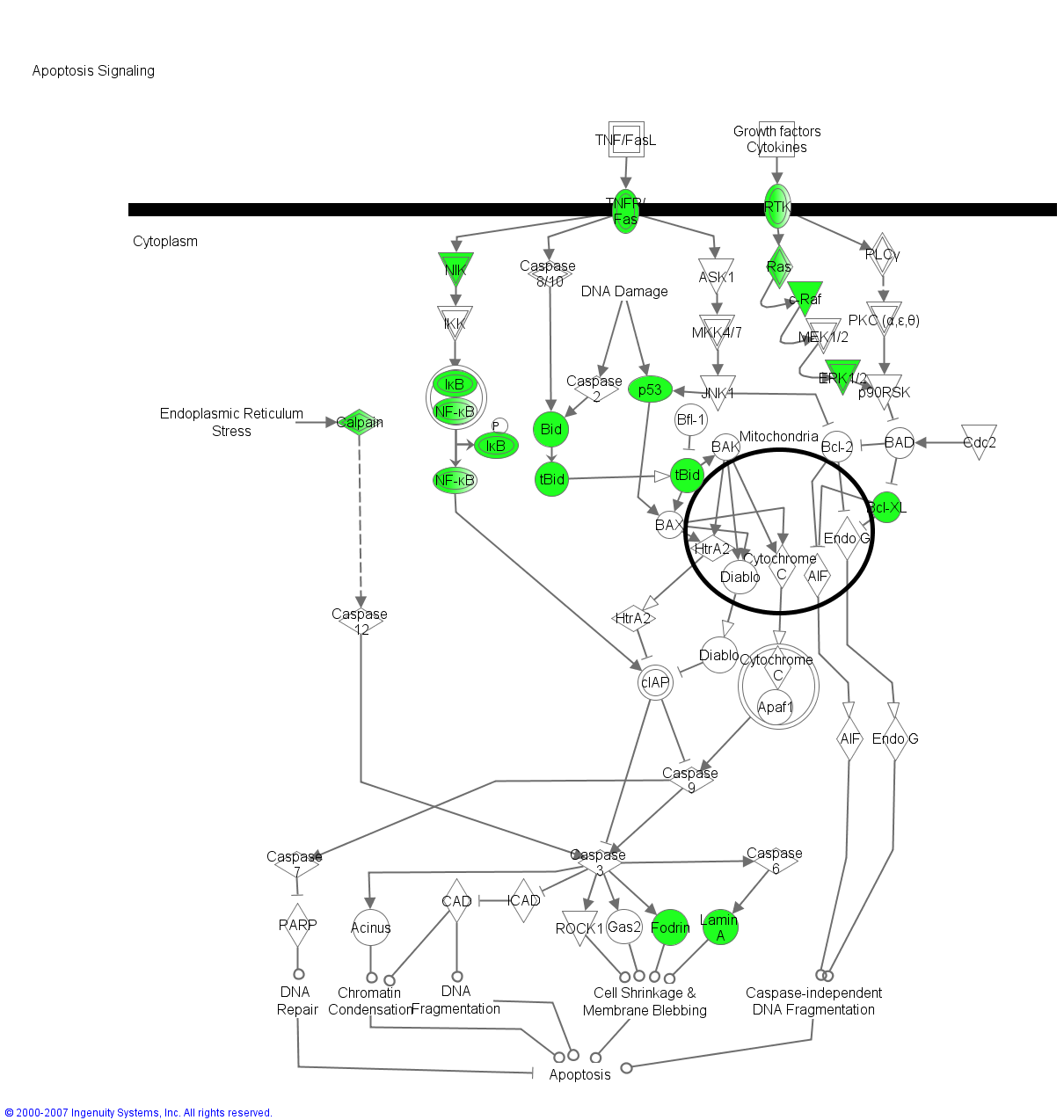


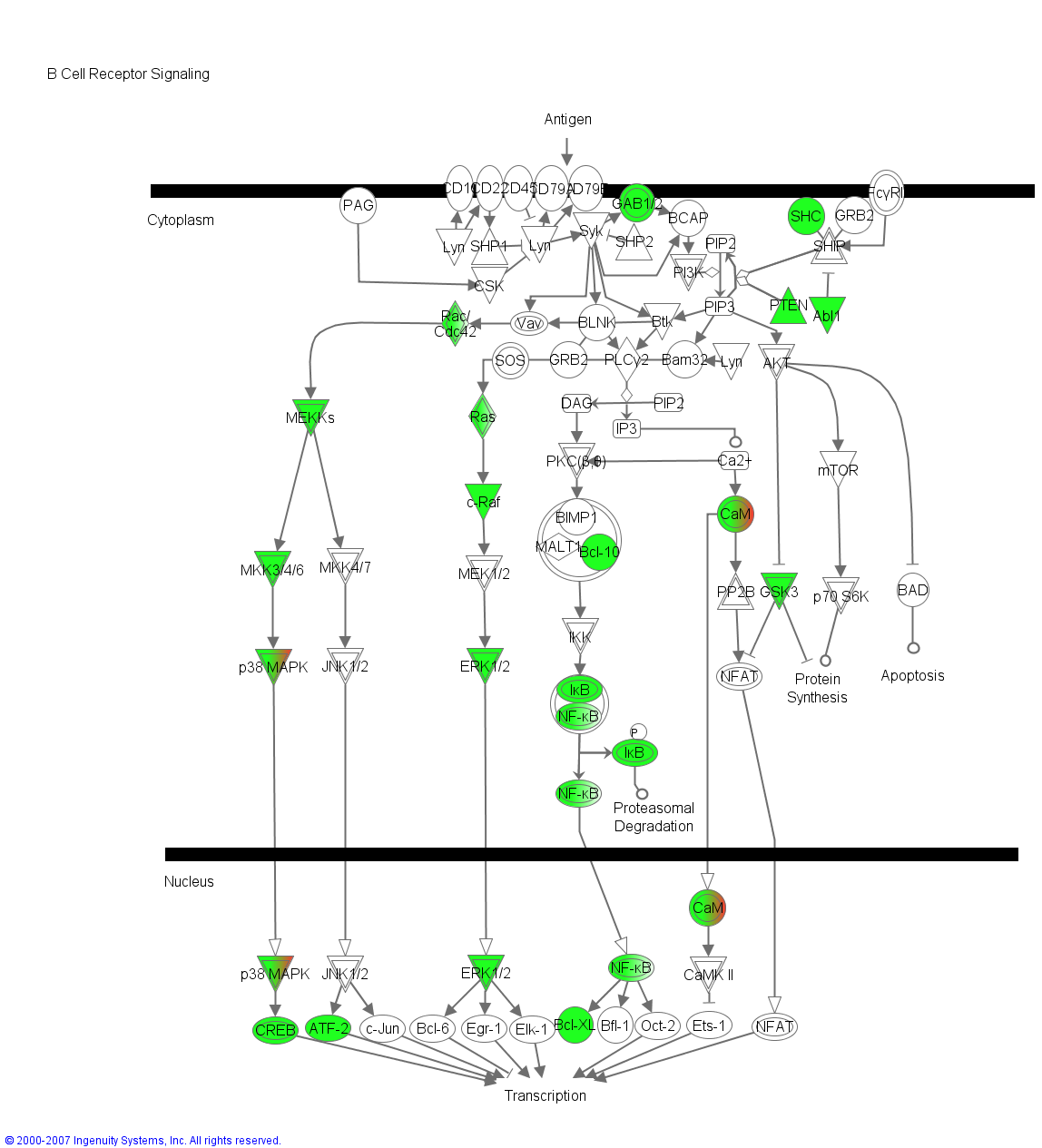


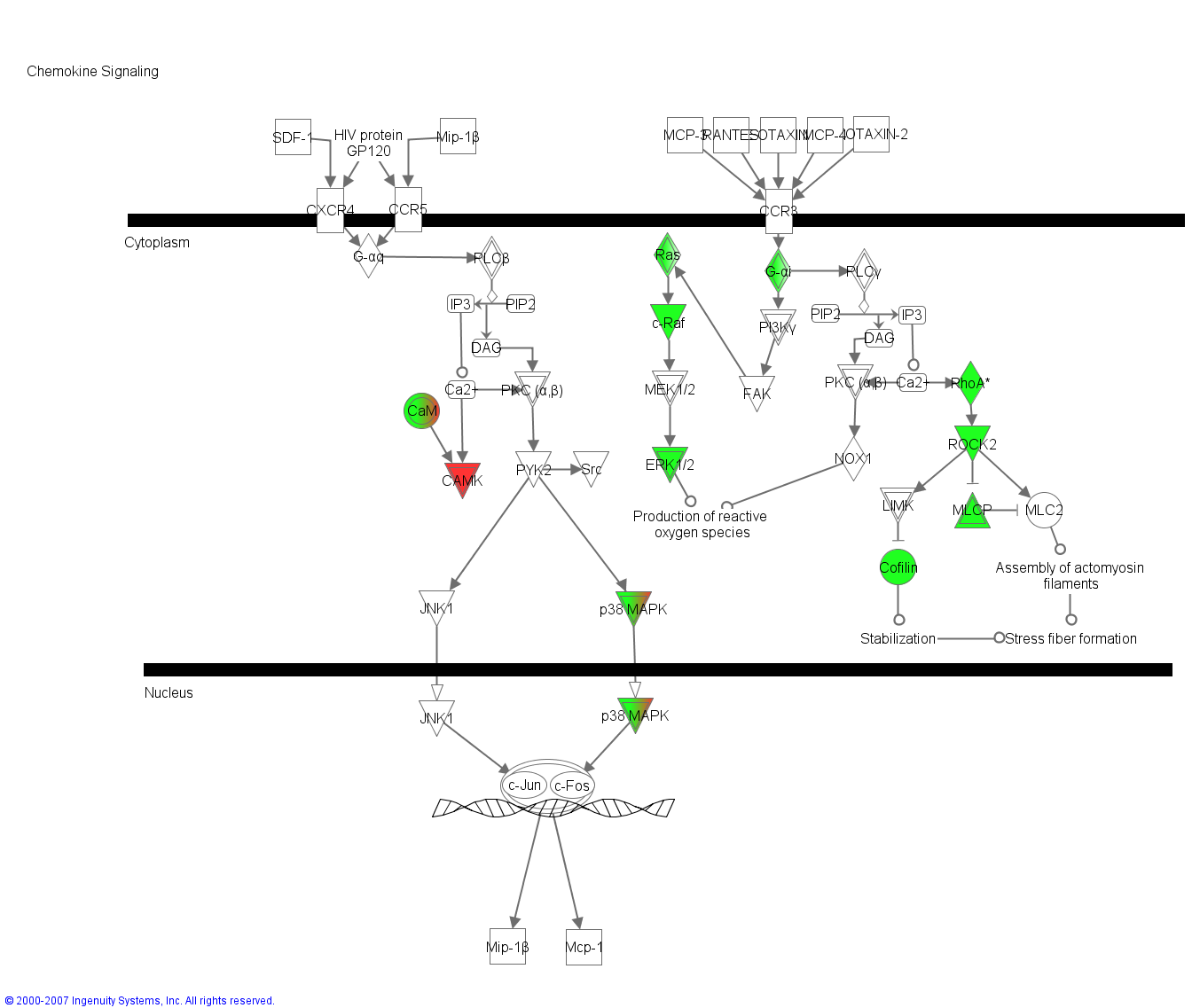


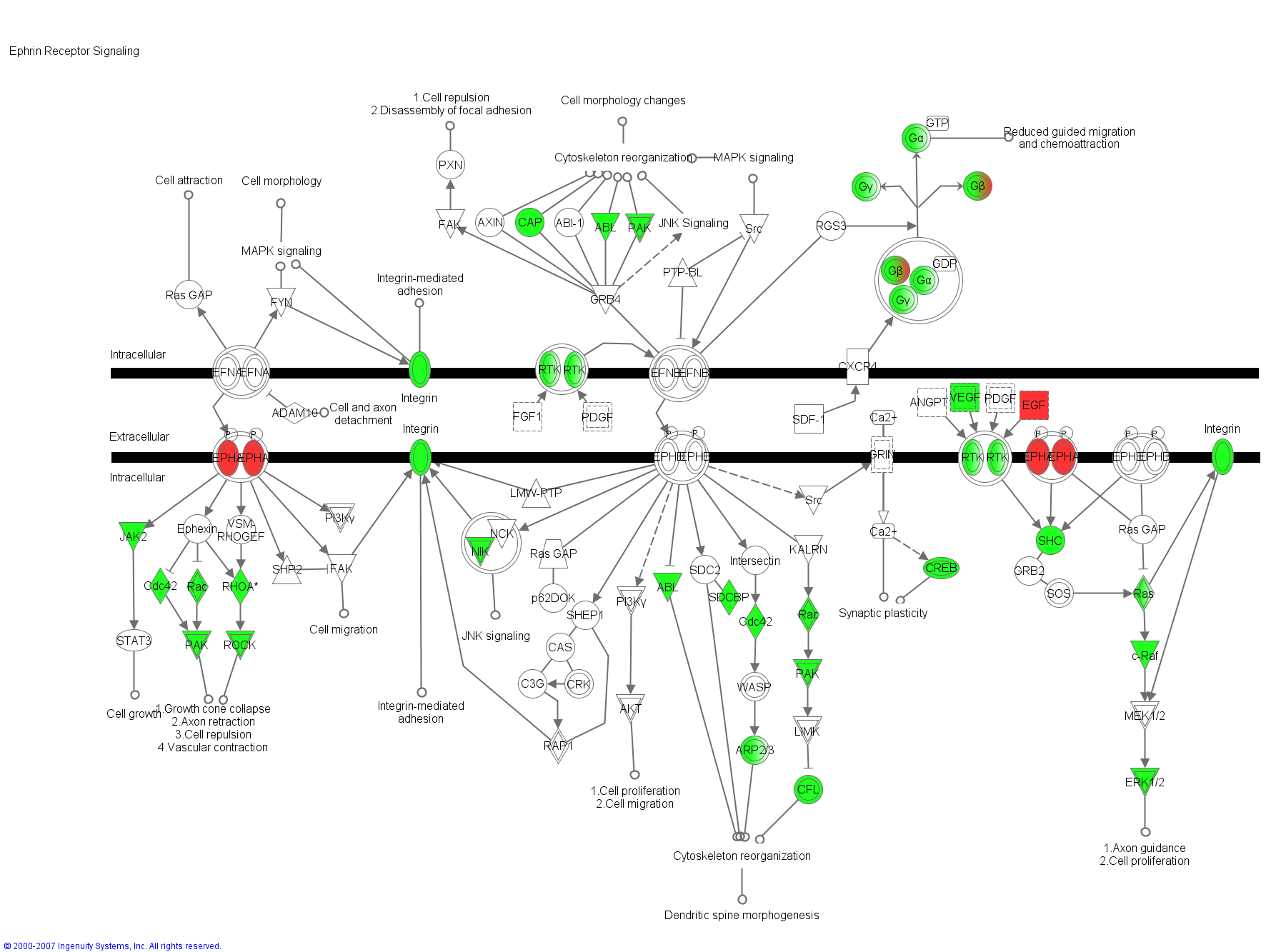


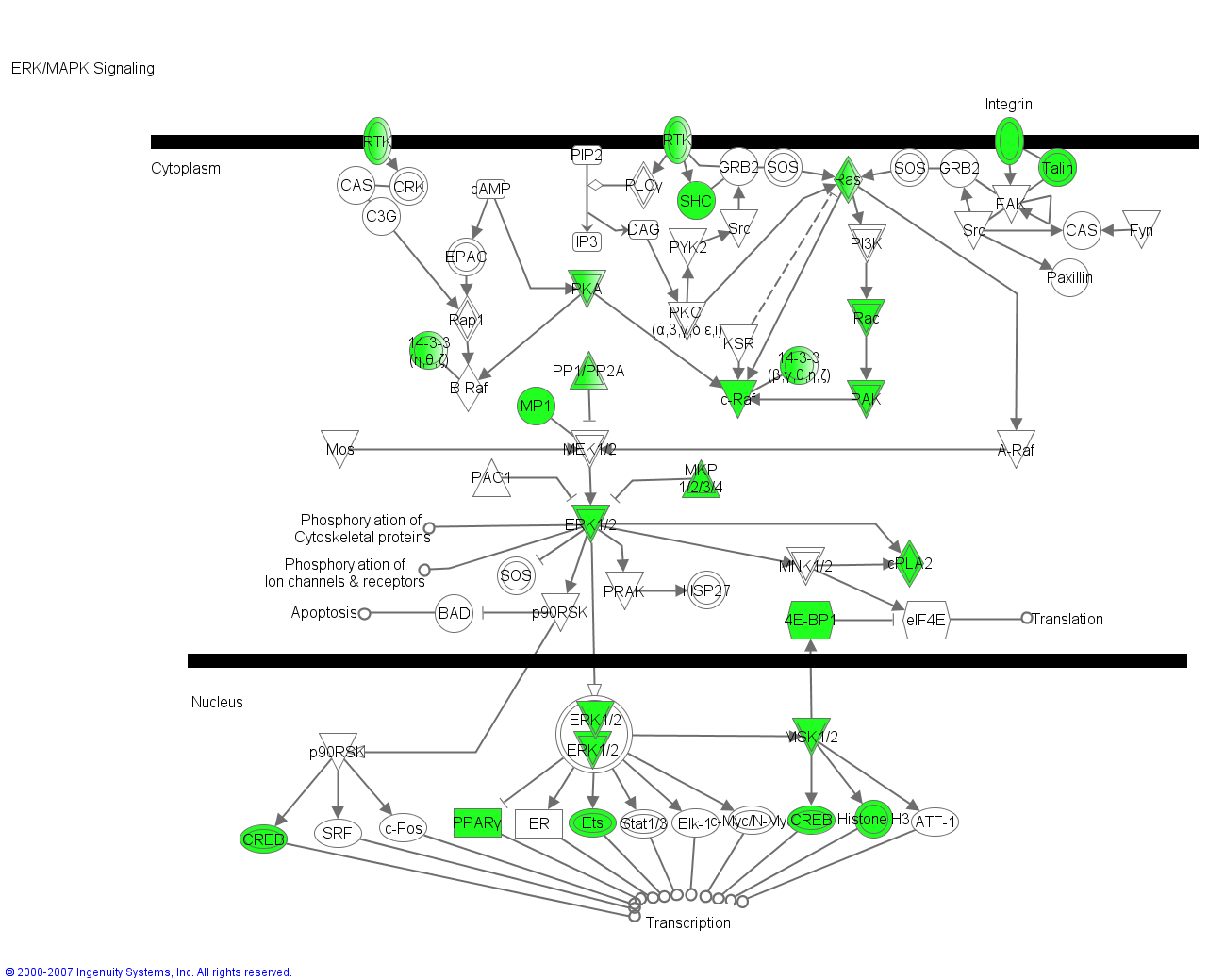


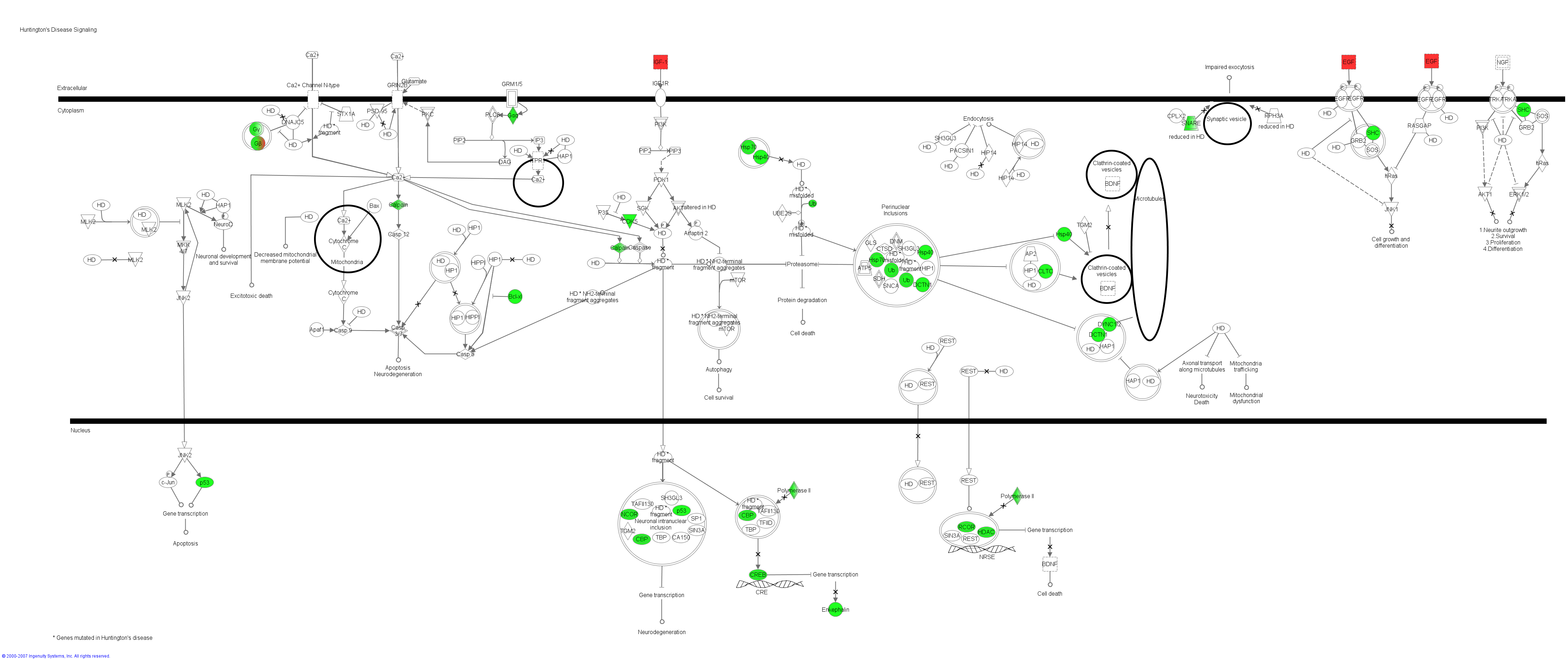


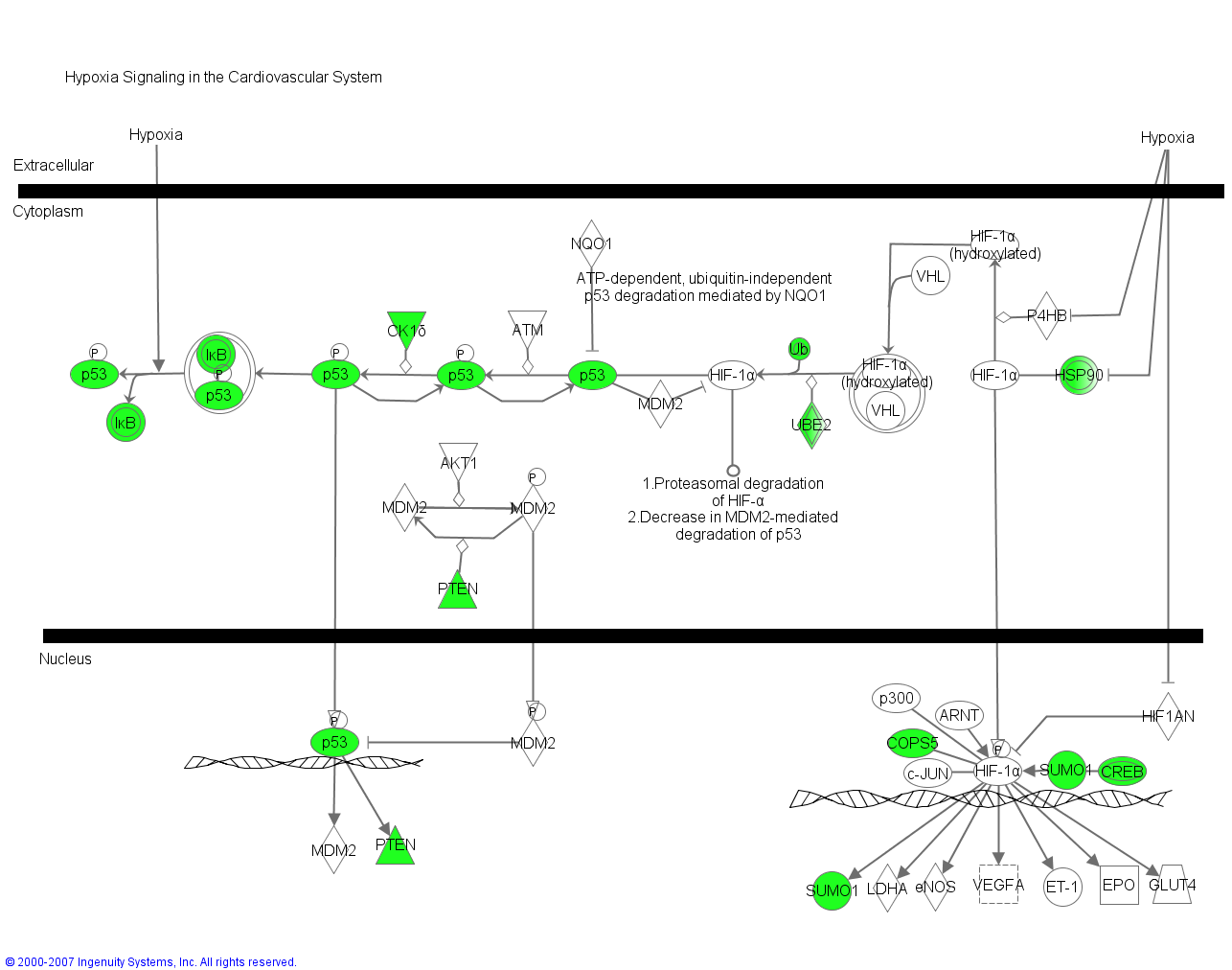


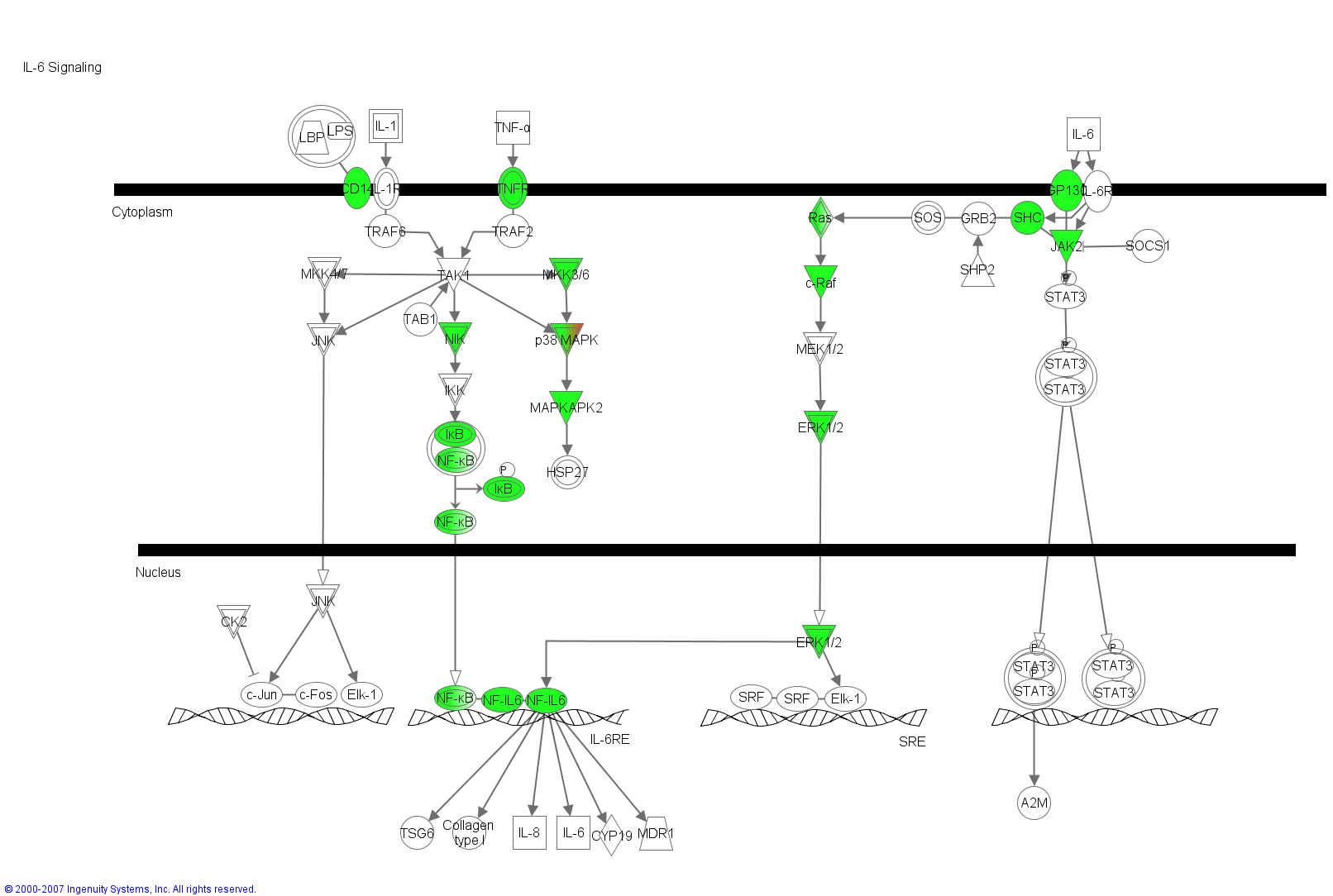


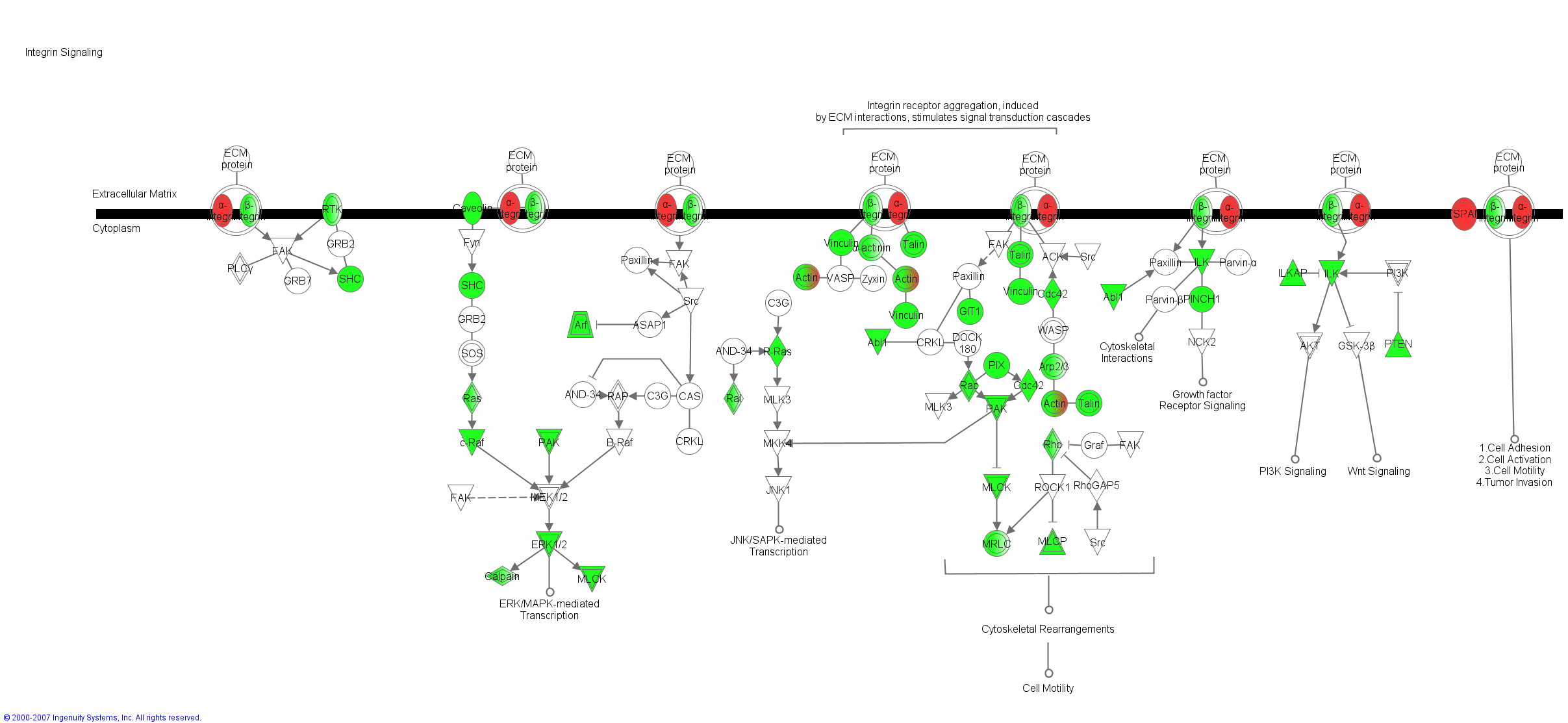


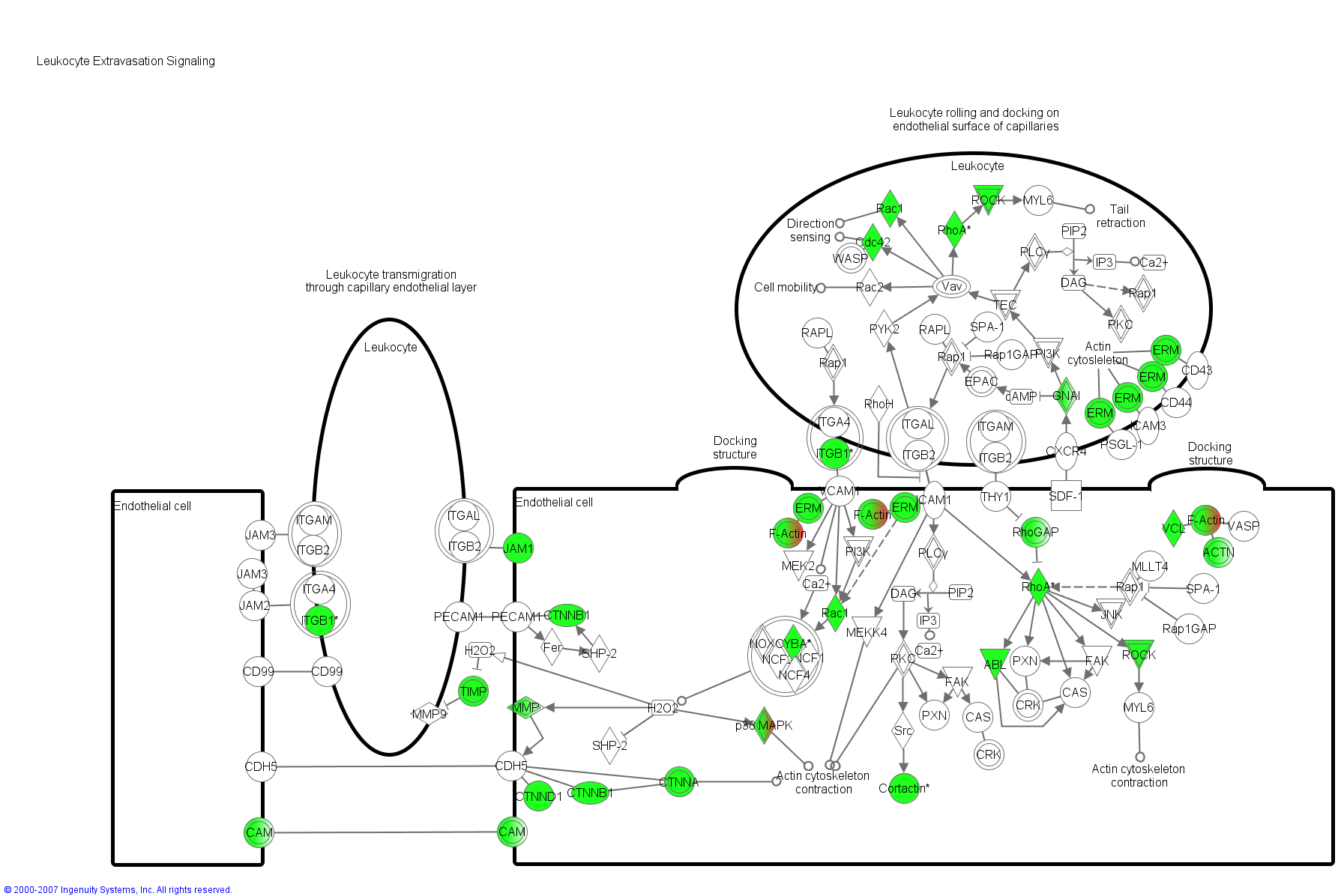


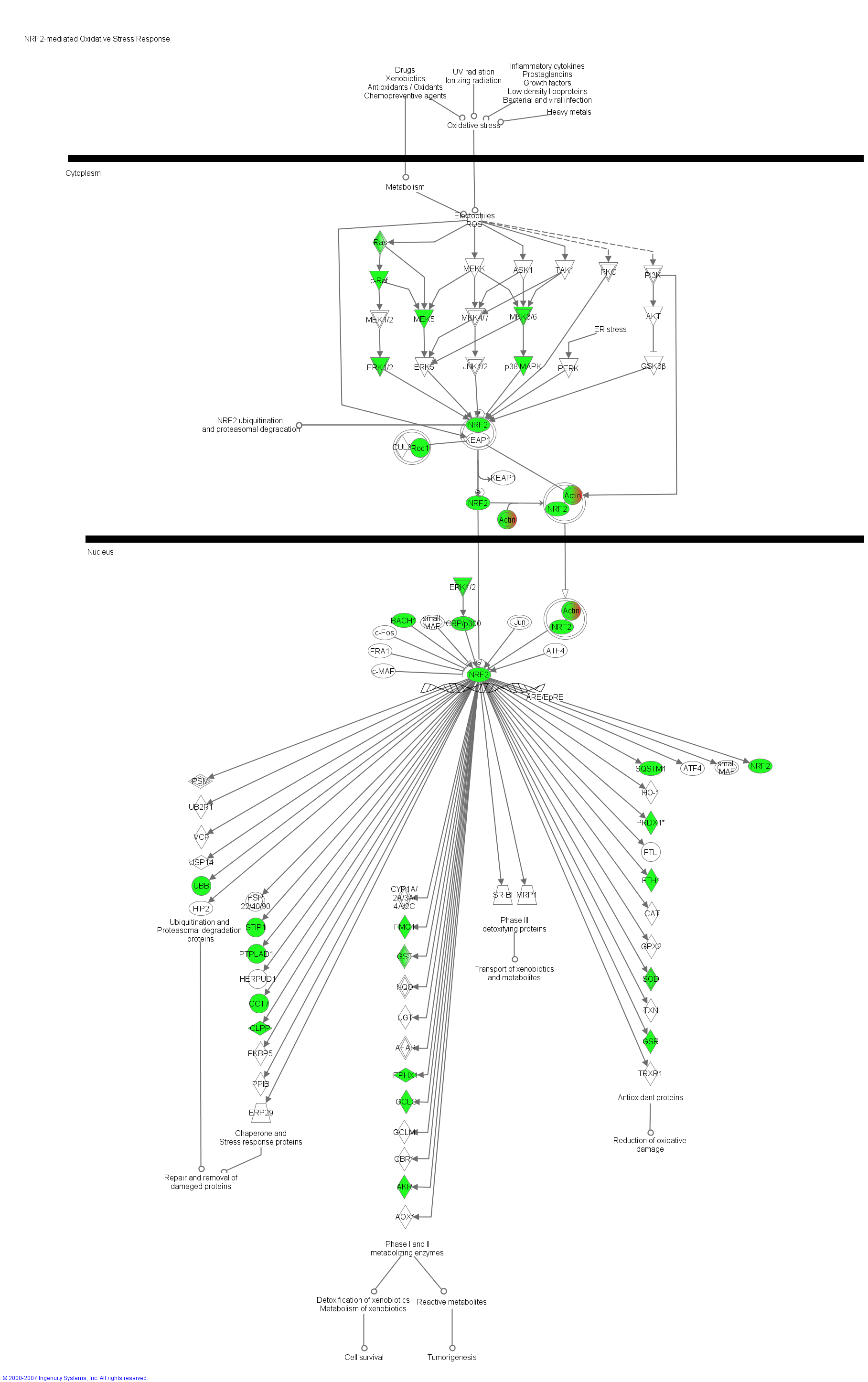


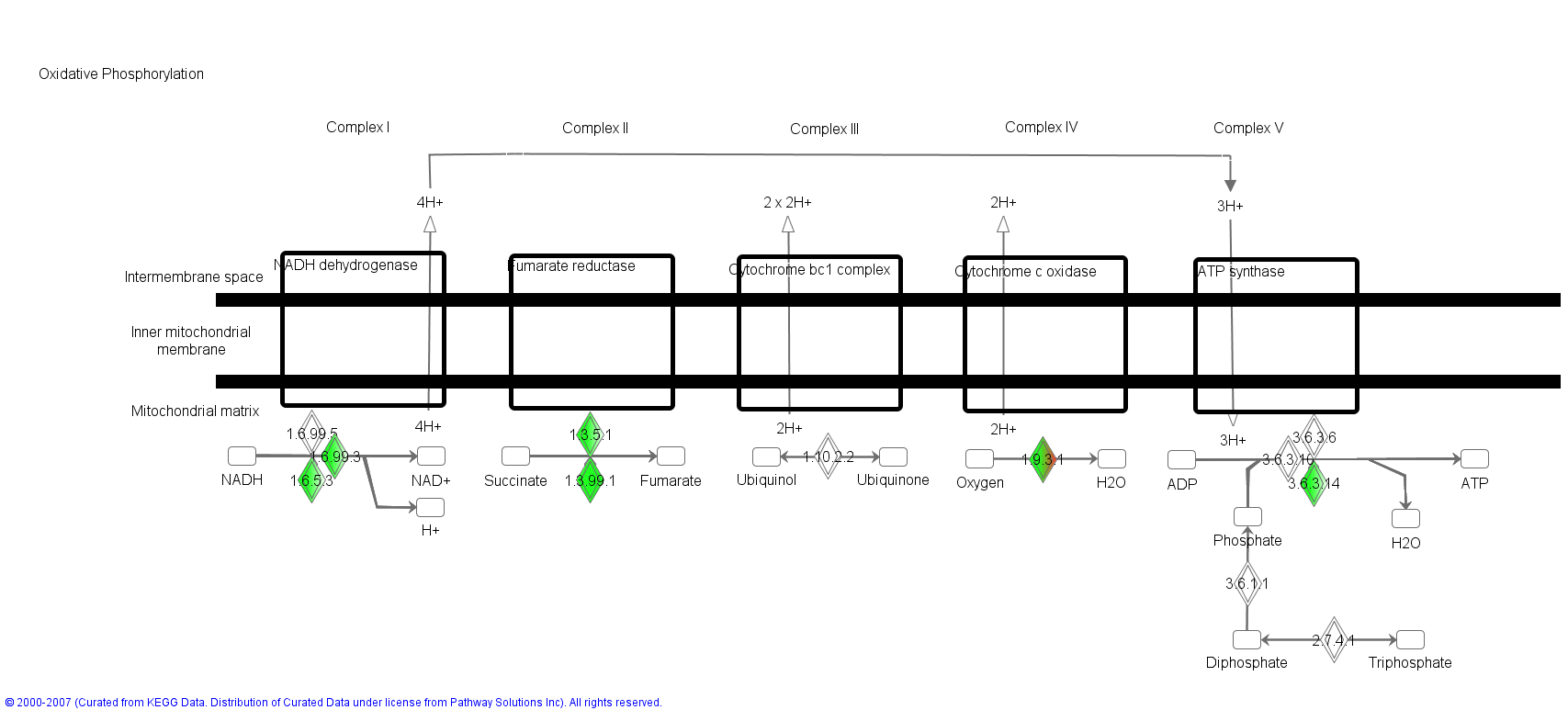


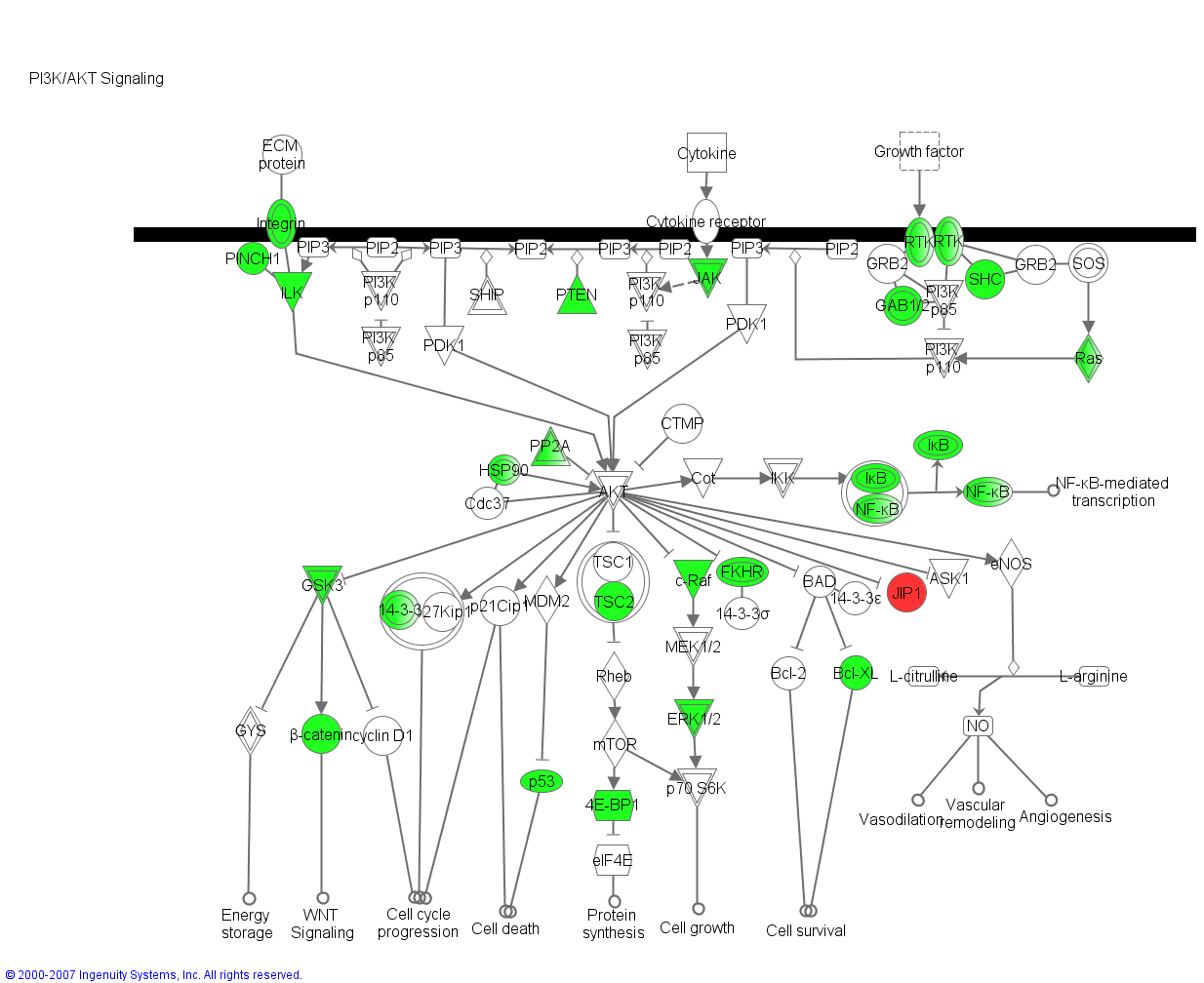


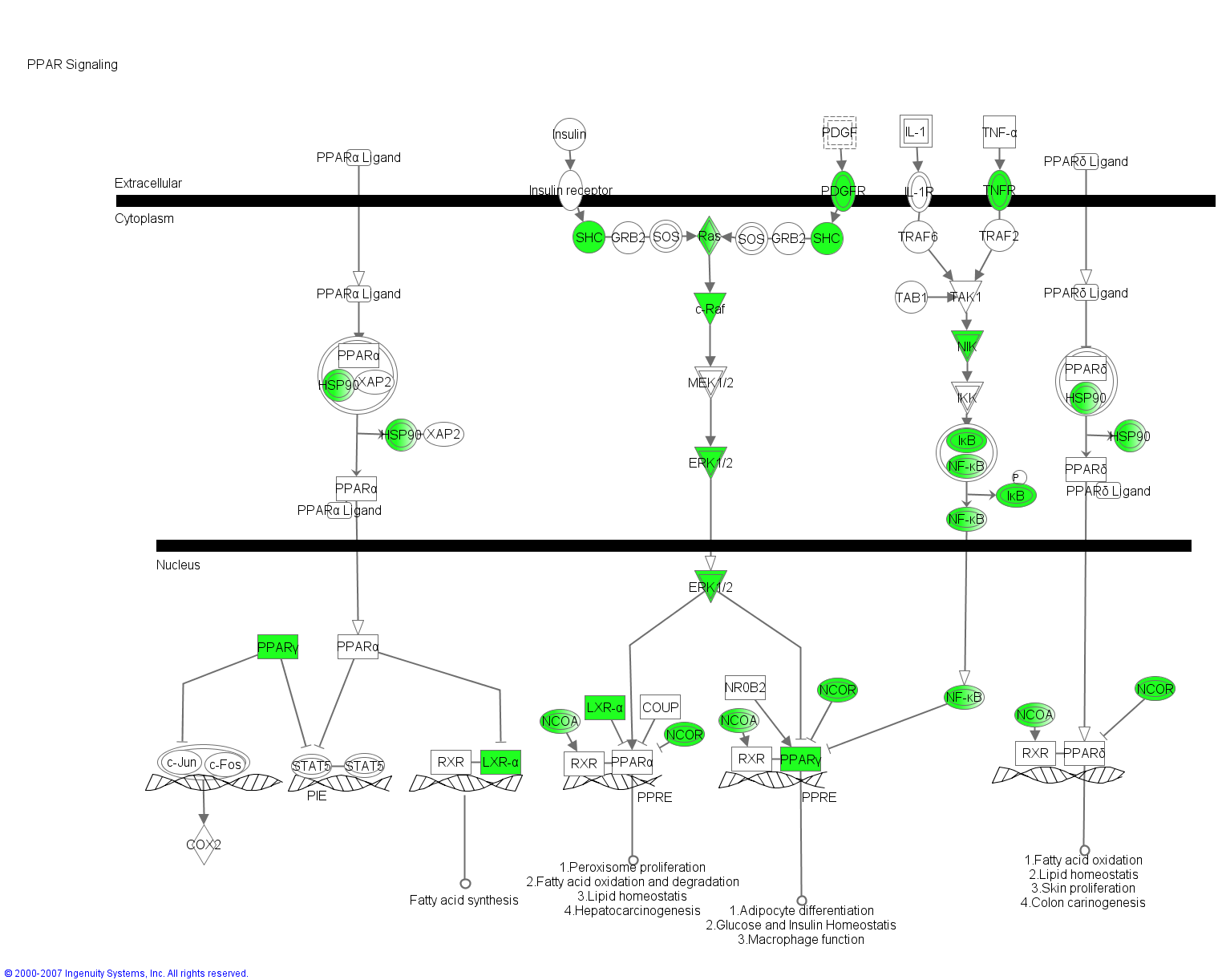


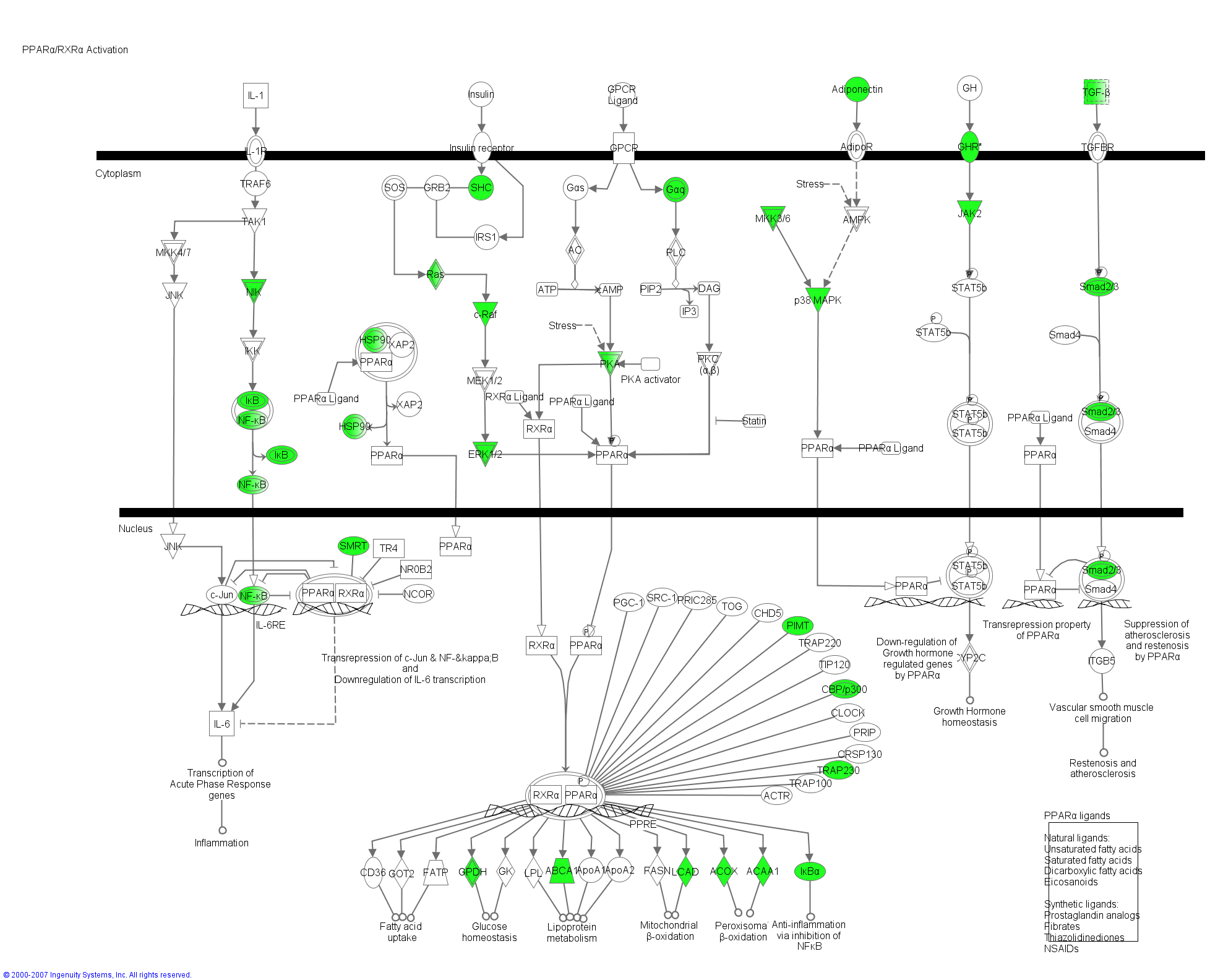


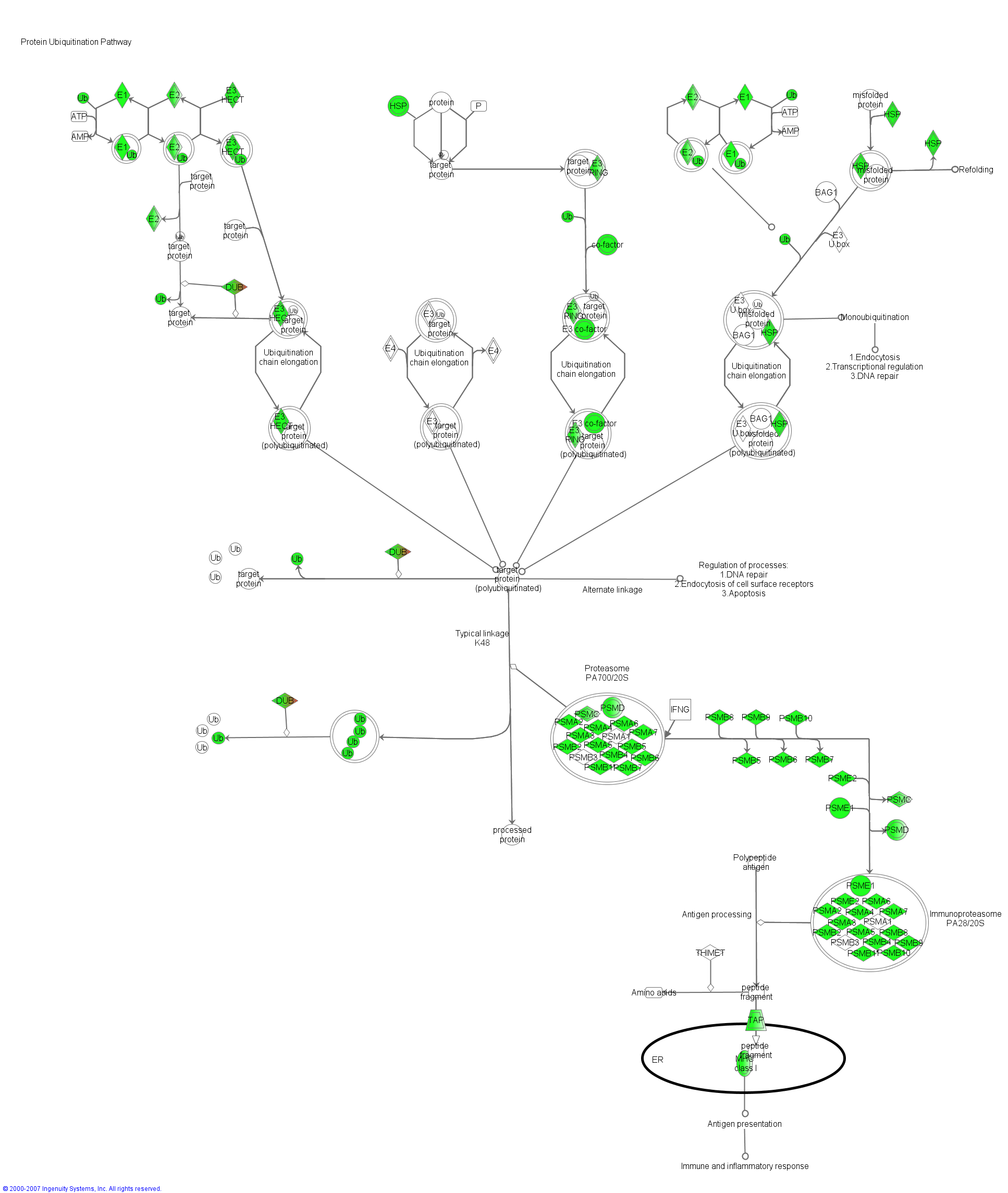


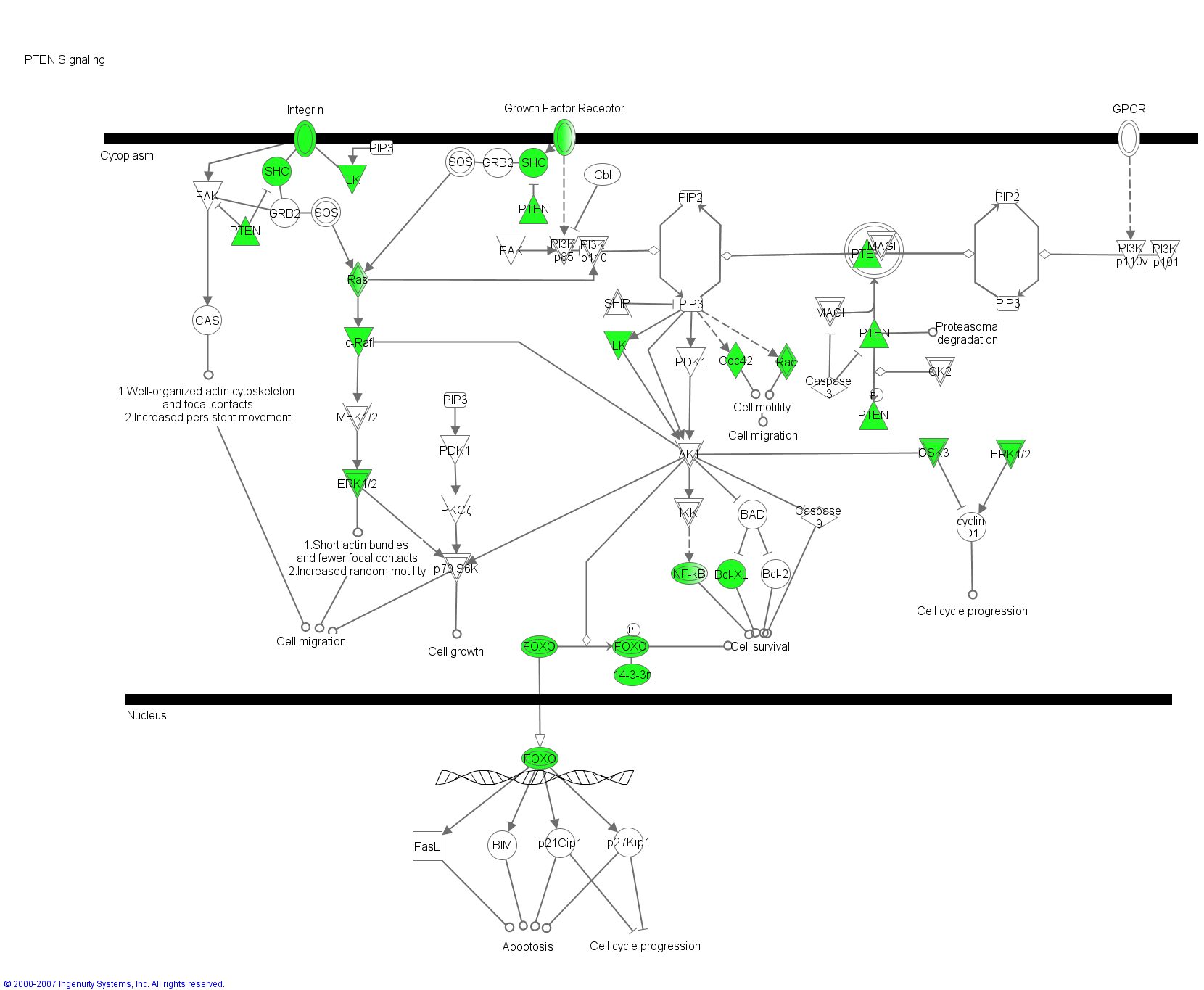


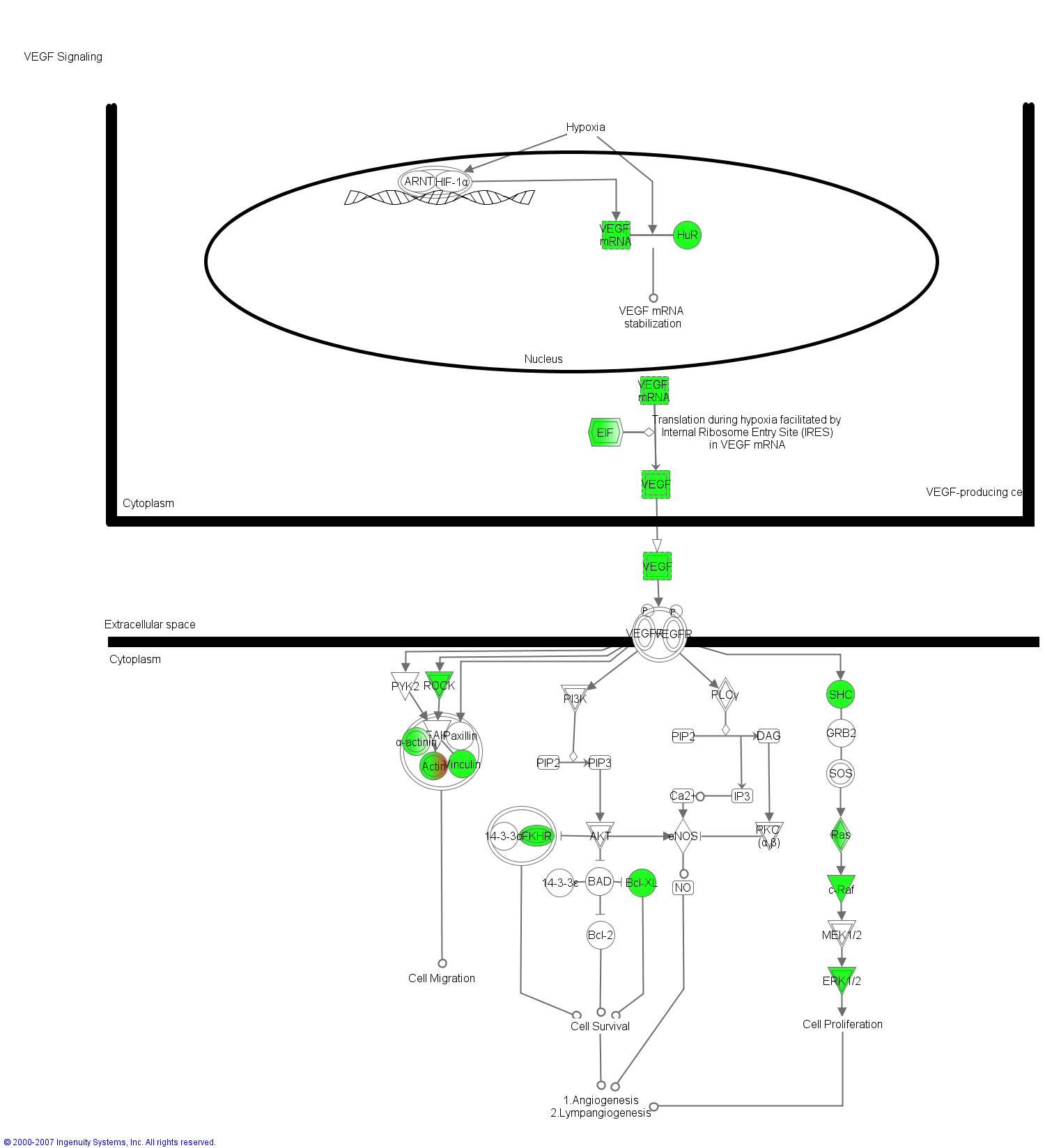


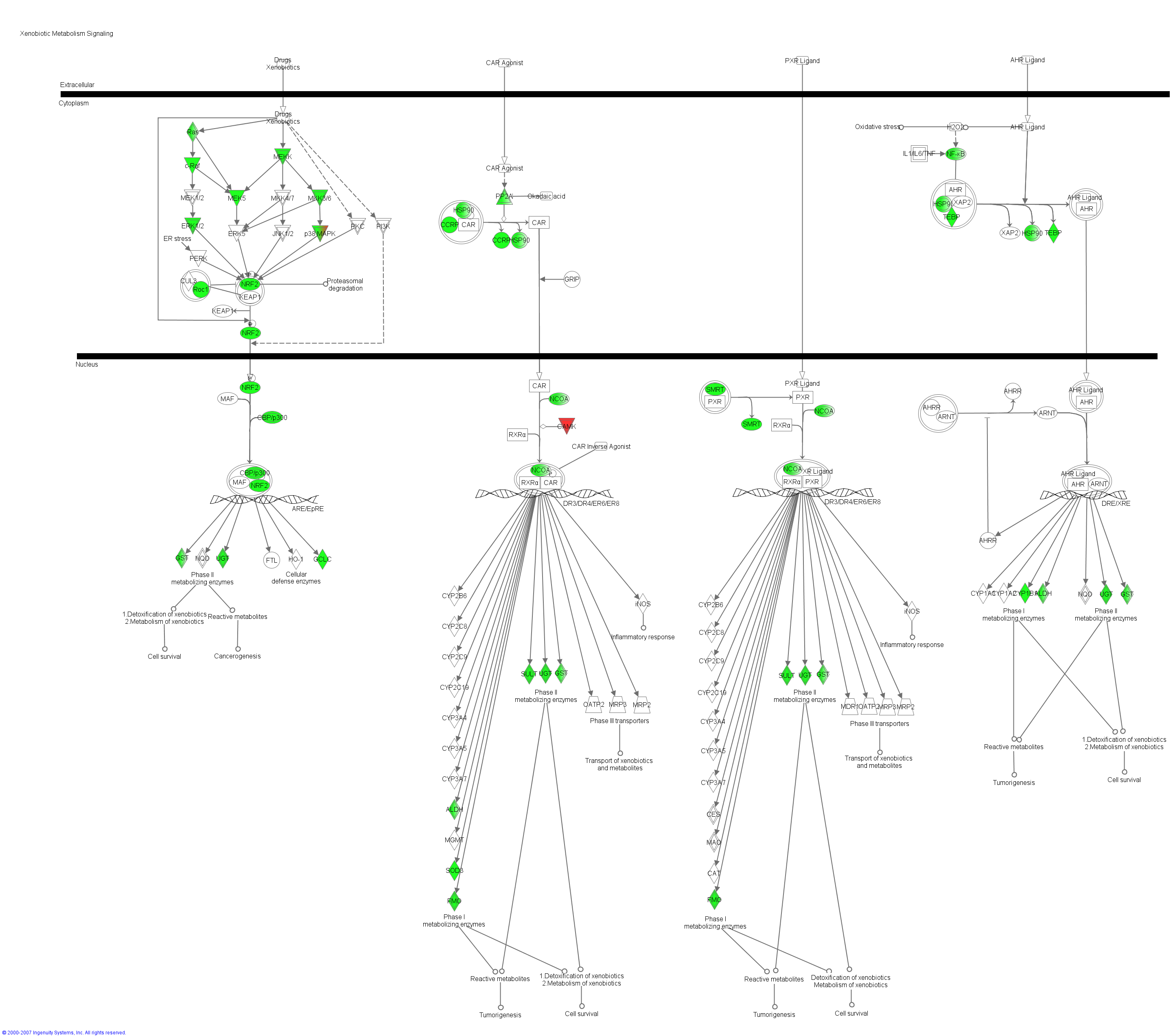

Supplement: Additional file 21 — Additional data file 21 is a Word document that contains pathway diagrams for the significant pathways associated with the Lactation Gene Set. [file 1752-0509-1-56-S21.doc]
