# Supplementary material for: Gene regulatory networks in lactation: identification of global principles using bioinformatics
Source: BMC Syst Biol. 2007 Nov 27;1:56. doi: 10.1186/1752-0509-1-56 (PMC2225983; doi:10.1186/1752-0509-1-56)

**Transcriptionally Regulated Pathways During Involution**

All pathways in this document are marginally significantly (unadjusted p < 0.05) enriched with genes from the Involution Gene Set. Highlighted pathways are significant after a Benjamini and Hochberg multiple testing correction. See additional data file 19 for the complete list of pathways and associated molecules from the Involution Gene Set.

| Pathway | B&H adjusted p-value | unadjusted p-value |
| --- | --- | --- |
| Protein Ubiquitination Pathway | 0 | 0 |
| PI3K/AKT Signaling | 0 | 0 |
| Integrin Signaling | 0 | 0 |
| NRF2-mediated Oxidative Stress Response | 0 | 0 |
| PPARα/RXRα Activation | 0.004342857 | 0.0002 |
| Apoptosis Signaling | 0.004342857 | 0.0002 |
| Ephrin Receptor Signaling | 0.004342857 | 0.0002 |
| ERK/MAPK Signaling | 0.0057 | 0.0003 |
| Estrogen Receptor Signaling | 0.008290909 | 0.0005 |
| Huntington's Disease Signaling | 0.008290909 | 0.0006 |
| Actin Cytoskeleton Signaling | 0.008290909 | 0.0006 |
| B Cell Receptor Signaling | 0.010133333 | 0.0008 |
| IGF-1 Signaling | 0.012861538 | 0.0011 |
| Insulin Receptor Signaling | 0.016285714 | 0.0015 |
| PTEN Signaling | 0.022293333 | 0.0022 |
| IL-6 Signaling | 0.024141176 | 0.0026 |
| VEGF Signaling | 0.024141176 | 0.0027 |
| Aryl Hydrocarbon Receptor Signaling | 0.024488889 | 0.0029 |
| Amyloid Processing | 0.0256 | 0.0032 |
| JAK/Stat Signaling | 0.03112381 | 0.0042 |
| PPAR Signaling | 0.03112381 | 0.0043 |
| GM-CSF Signaling | 0.037309091 | 0.0054 |
| Hypoxia Signaling in the Cardiovascular System | 0.042956522 | 0.0065 |
| Leukocyte Extravasation Signaling | 0.0475 | 0.0075 |
| Neuregulin Signaling | 0.053504 | 0.0088 |
| Oxidative Phosphorylation | 0.070153846 | 0.012 |
| Chemokine Signaling | 0.079257143 | 0.0143 |
| NF-κB Signaling | 0.079257143 | 0.0146 |
| Wnt/β-catenin Signaling | 0.125793103 | 0.024 |
| Xenobiotic Metabolism Signaling | 0.13832 | 0.0273 |
| Cell Cycle: G1/S Checkpoint Regulation | 0.142025 | 0.029 |
| T Cell Receptor Signaling | 0.142025 | 0.0299 |
| Synaptic Long Term Potentiation | 0.150618182 | 0.0327 |
| PDGF Signaling | 0.153737143 | 0.0354 |
| IL-10 Signaling | 0.153737143 | 0.0354 |
| Axonal Guidance Signaling | 0.160444444 | 0.038 |
| Nucleotide Excision Repair Pathway | 0.162681081 | 0.0396 |
| Antigen Presentation Pathway | 0.175384615 | 0.0442 |
| Toll-like Receptor Signaling | 0.175384615 | 0.045 |
| Death Receptor Signaling | 0.18734 | 0.0493 |


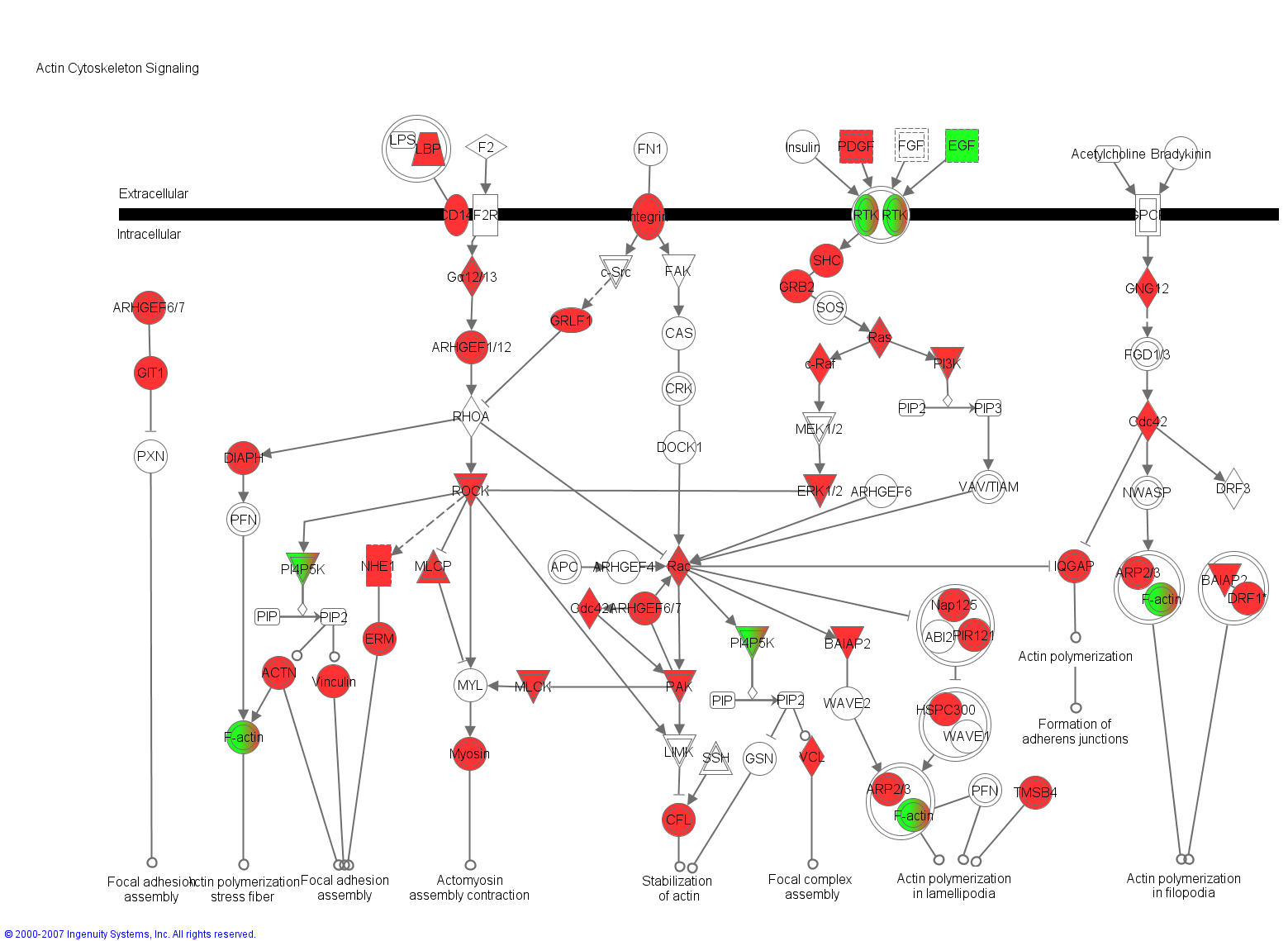


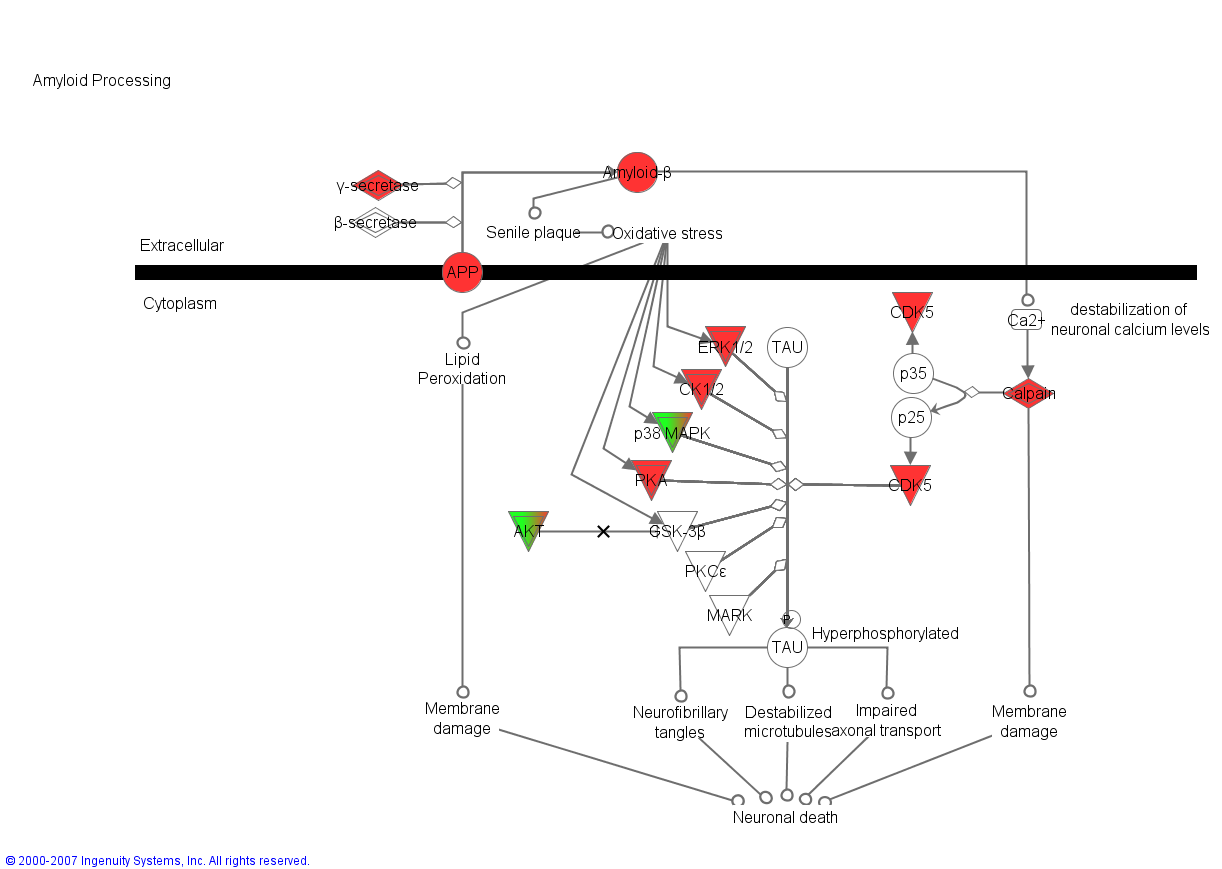


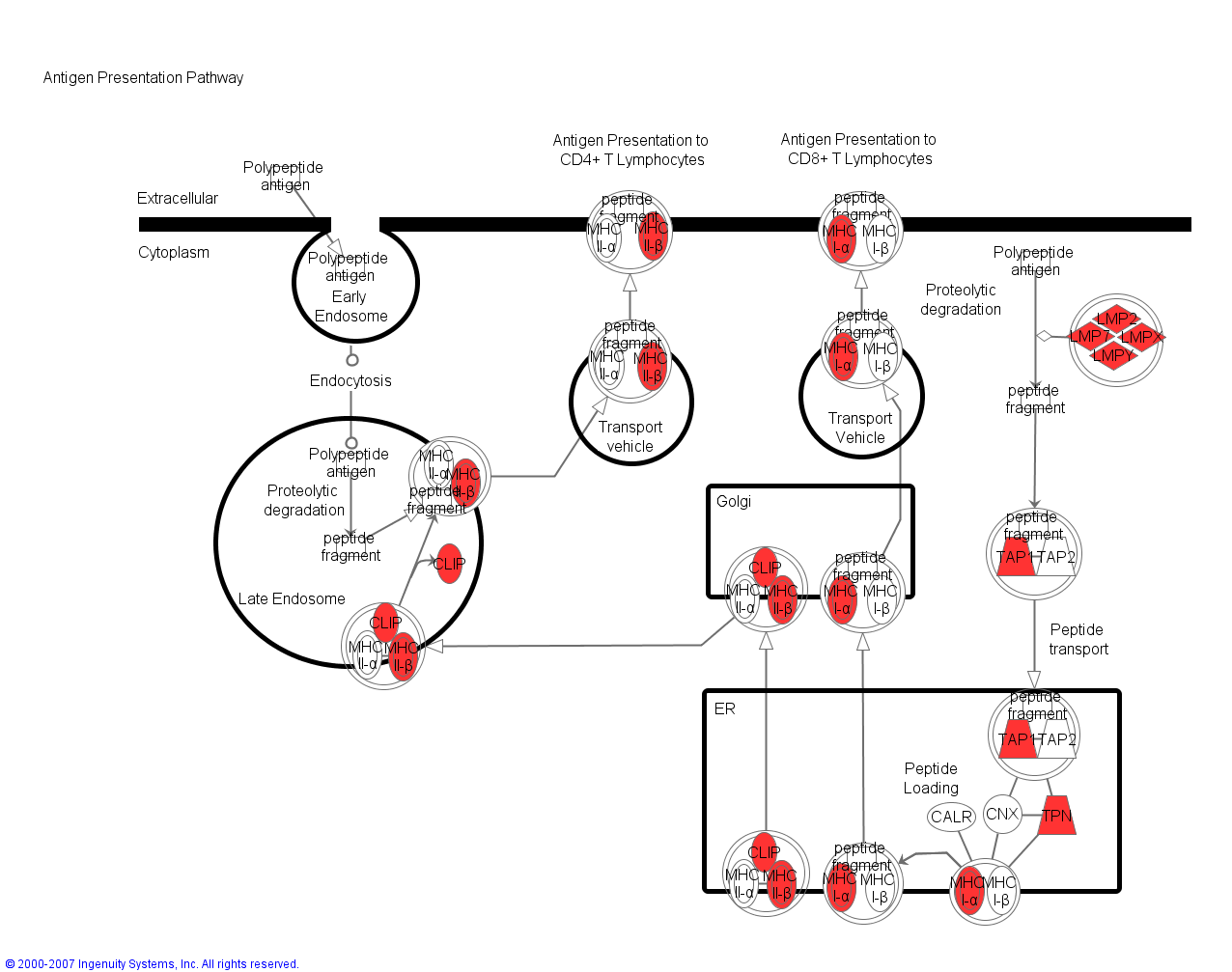


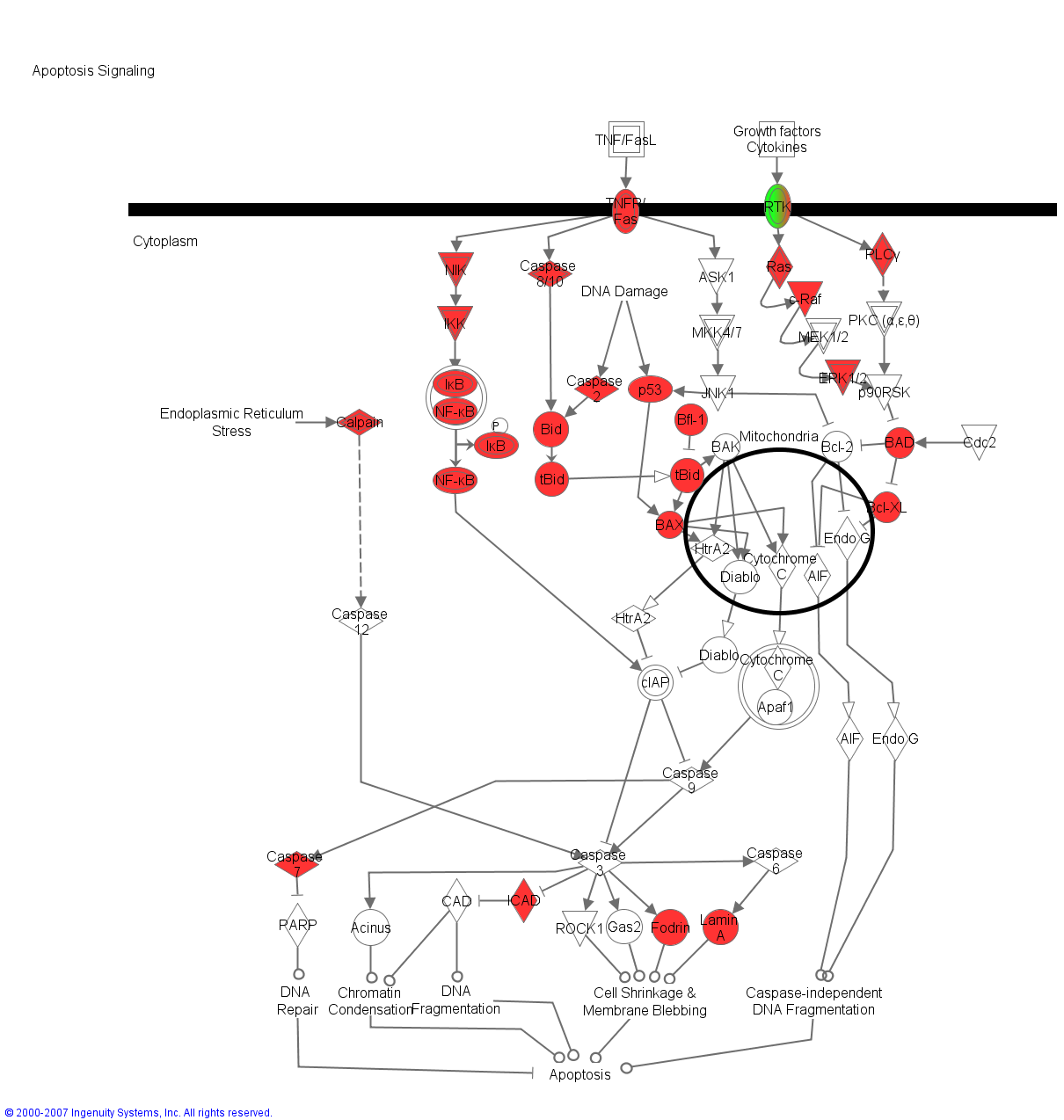


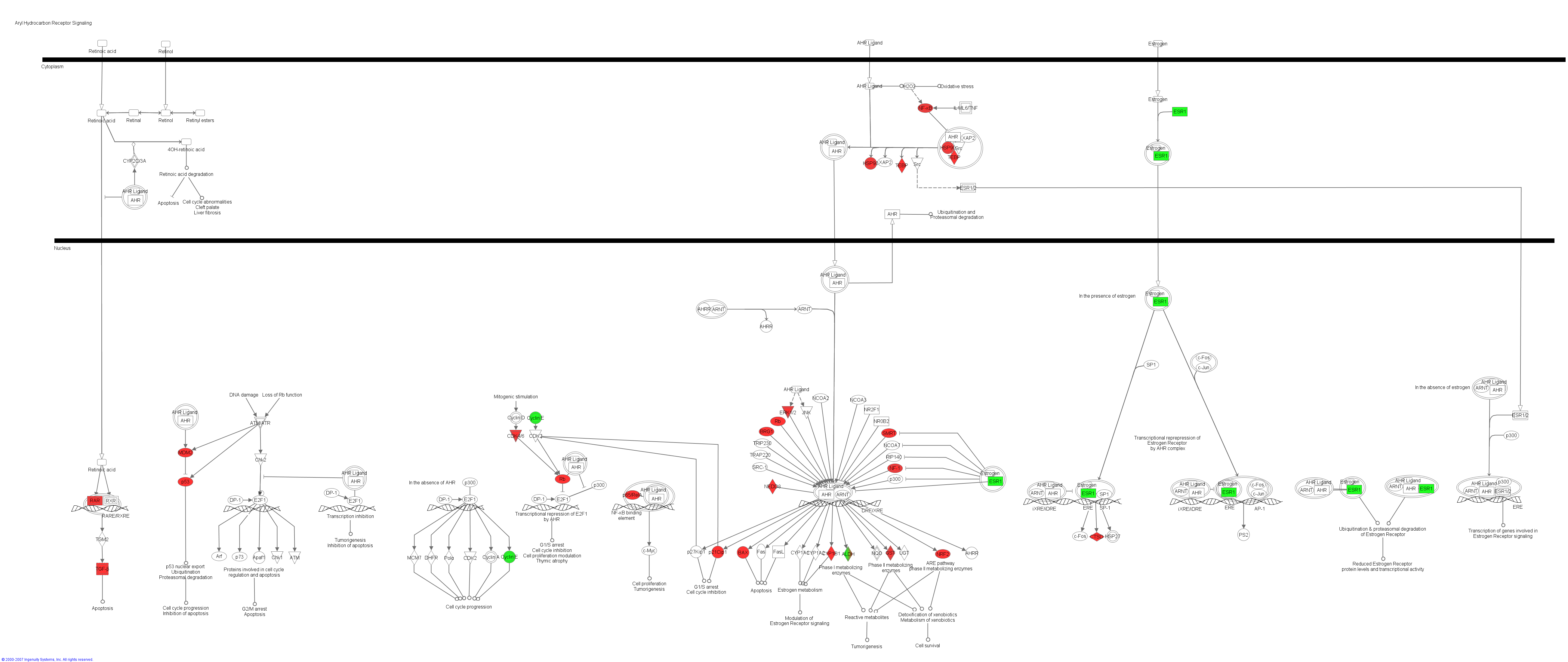


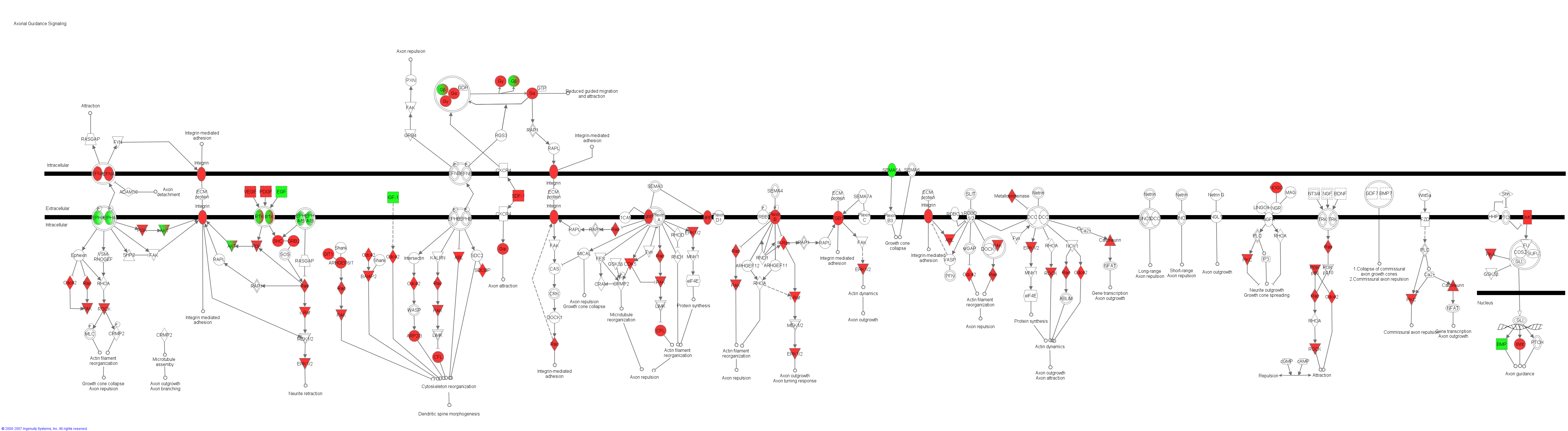


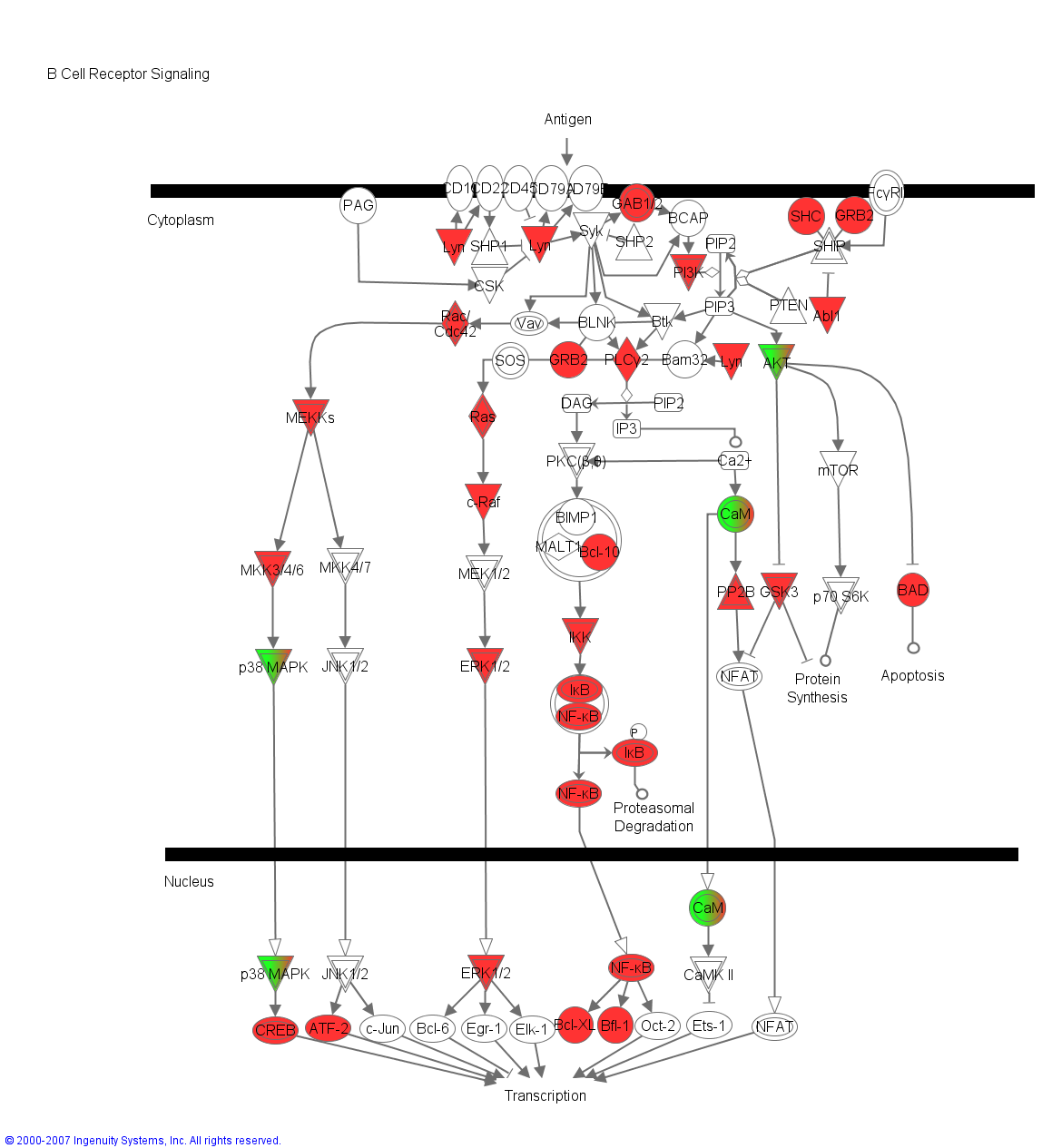


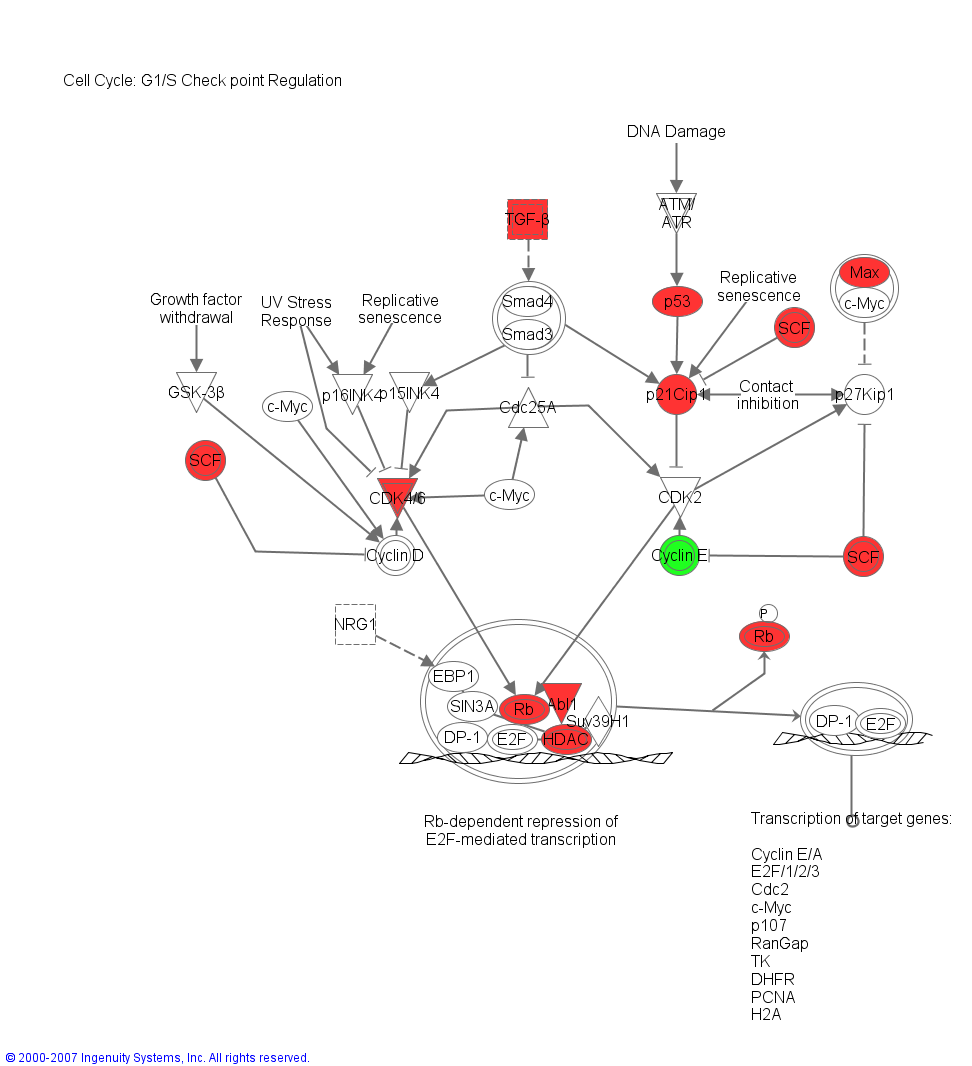


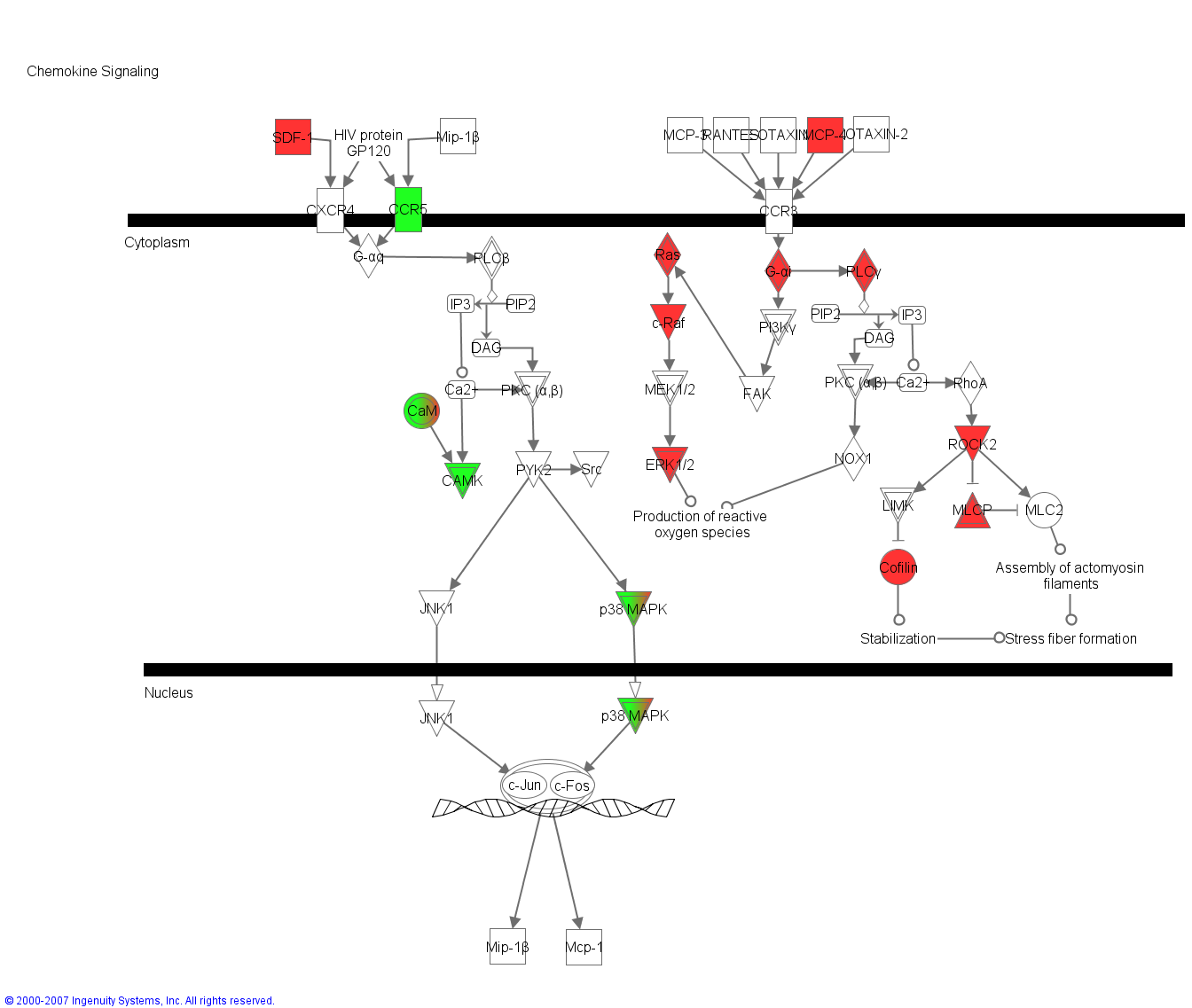


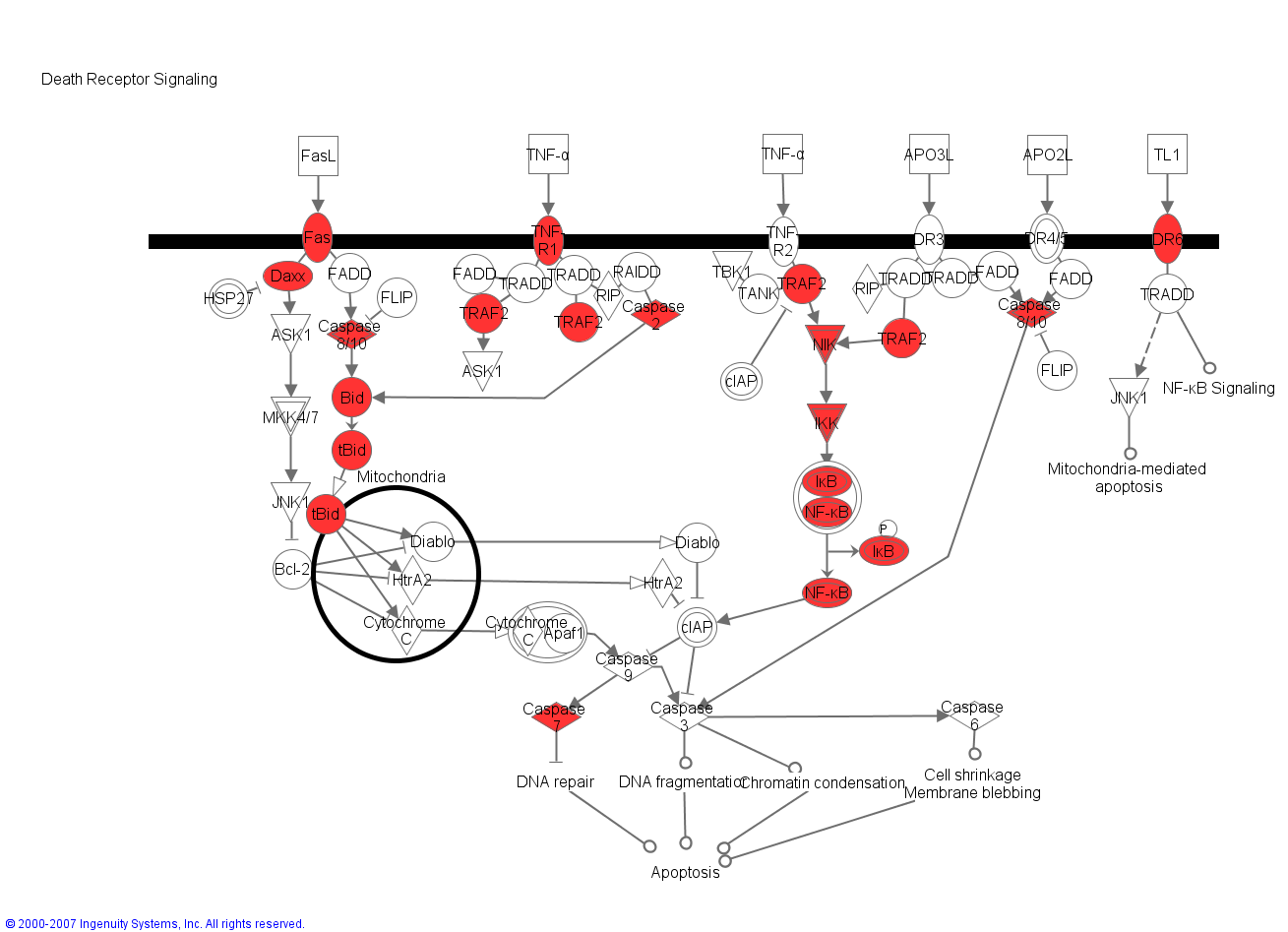


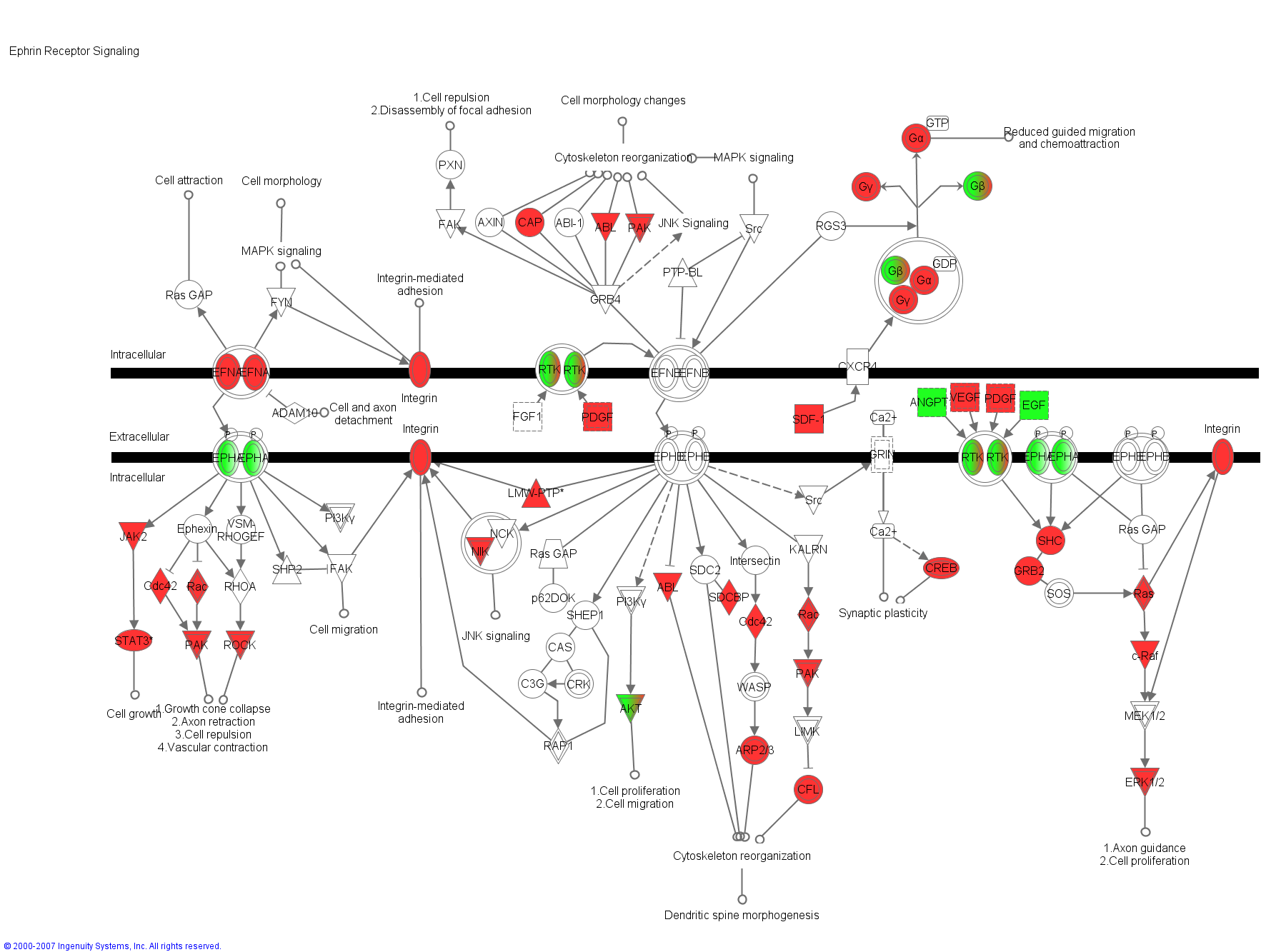


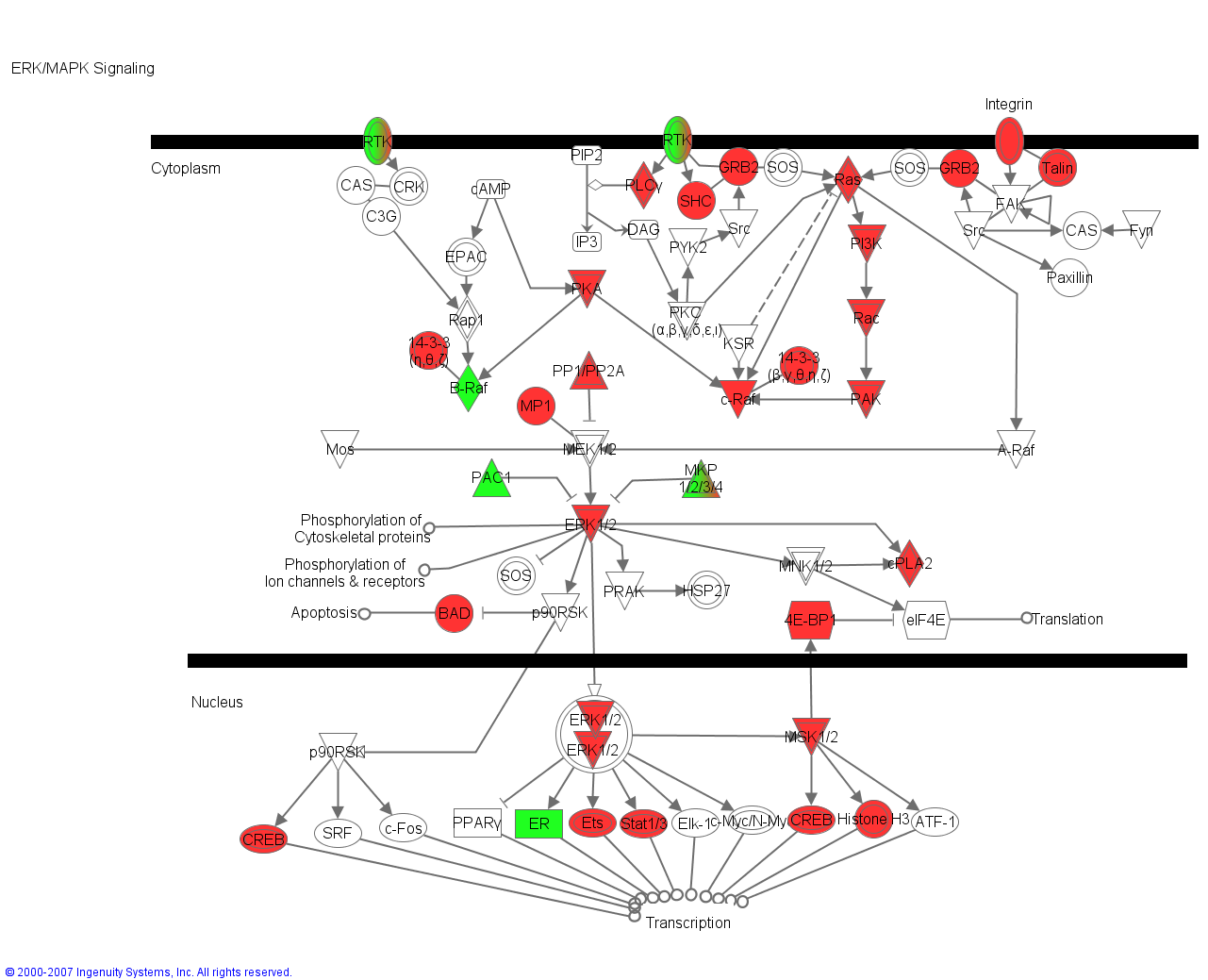


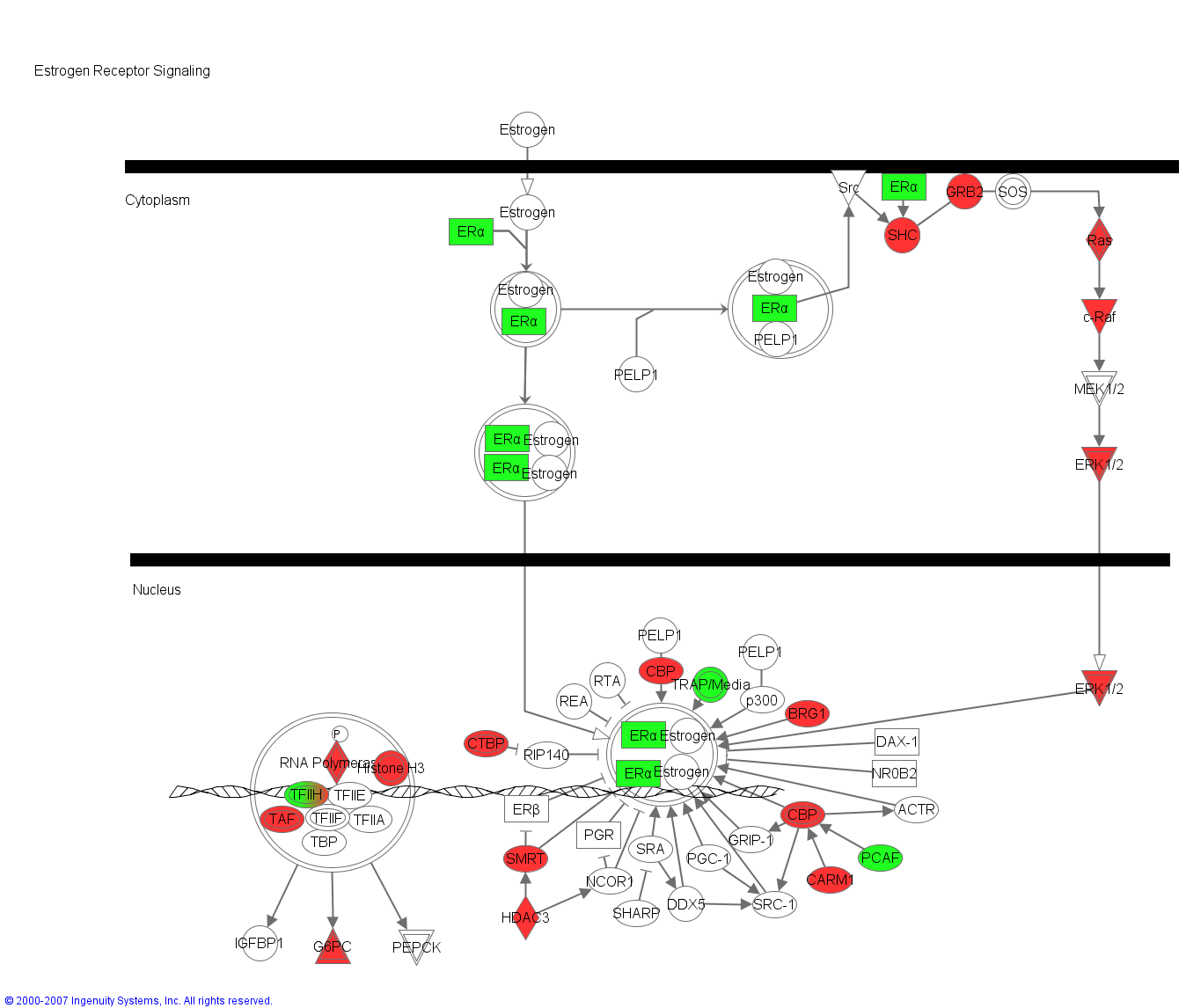


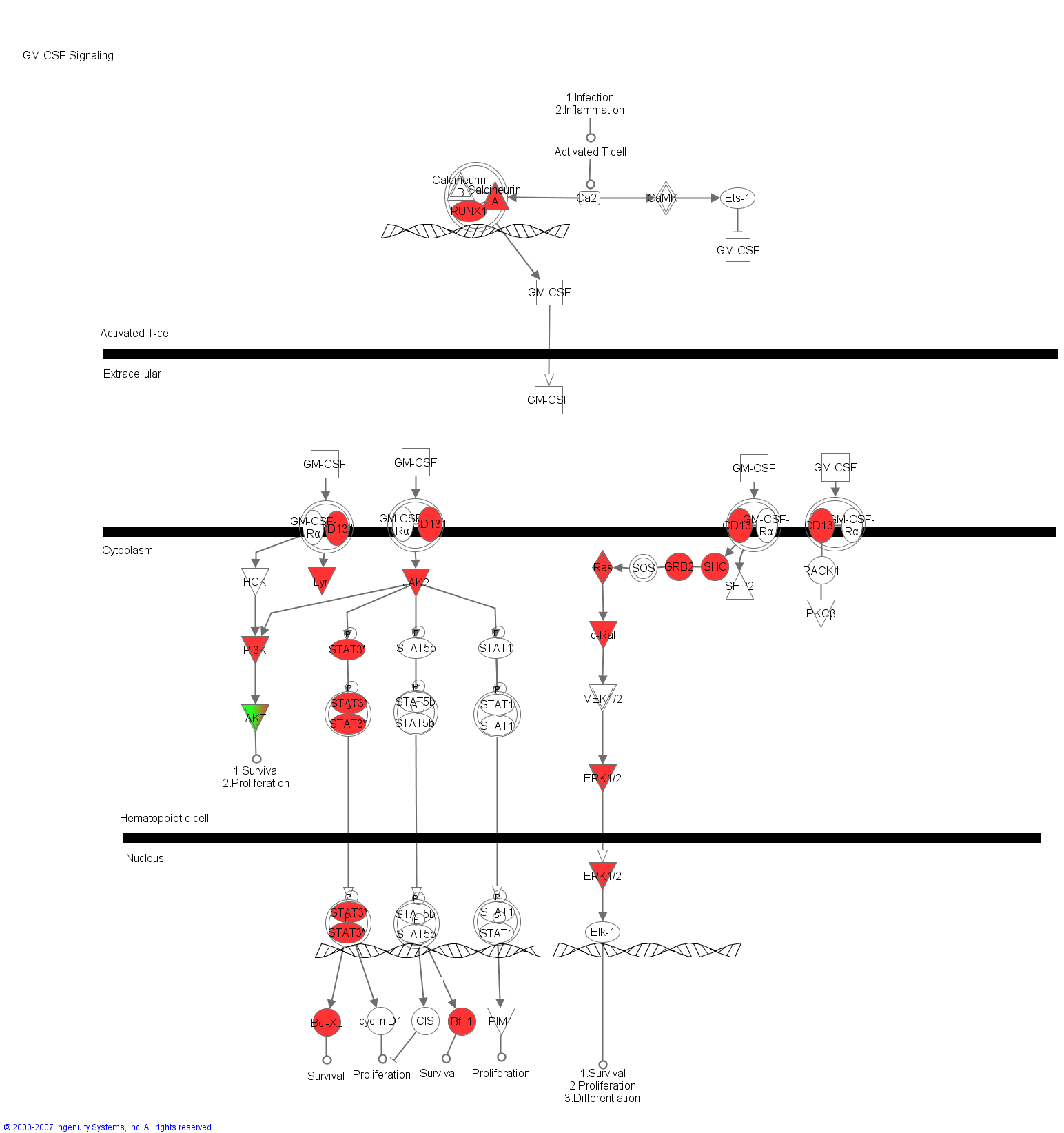


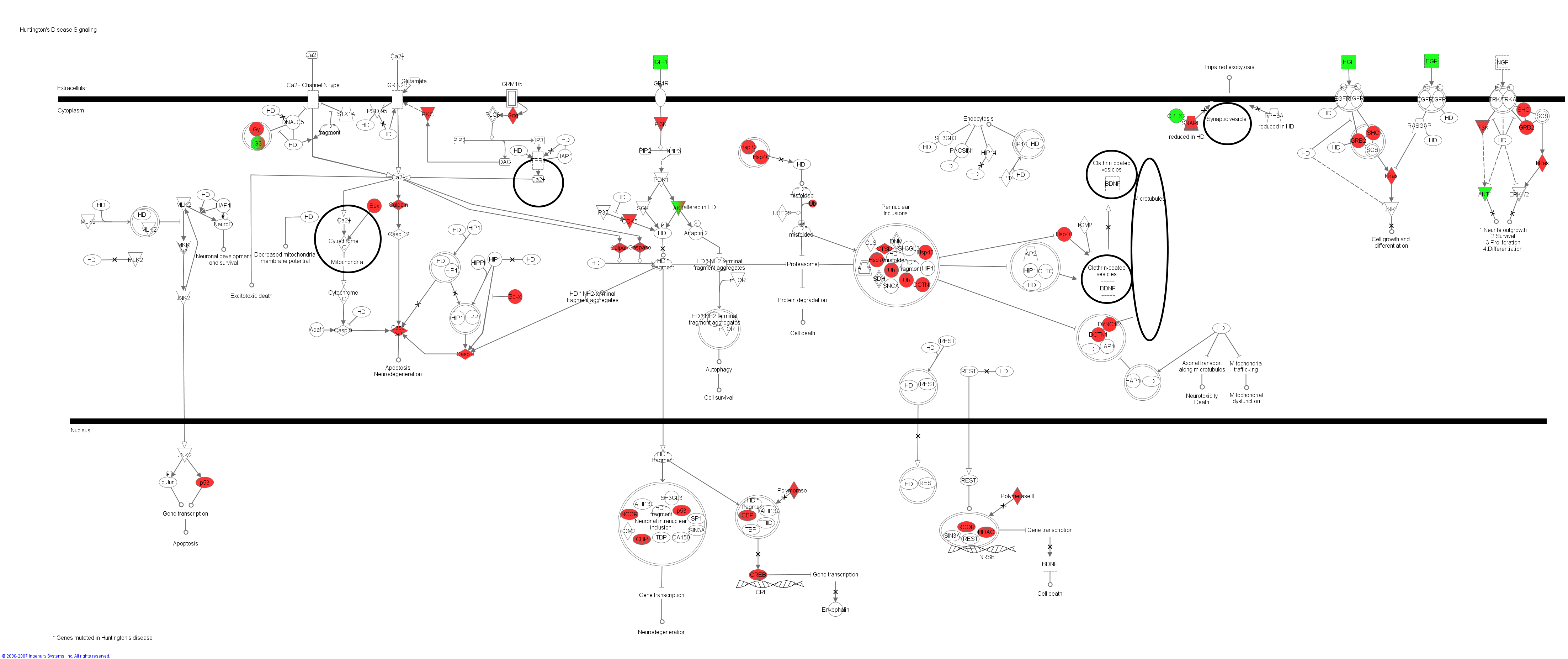


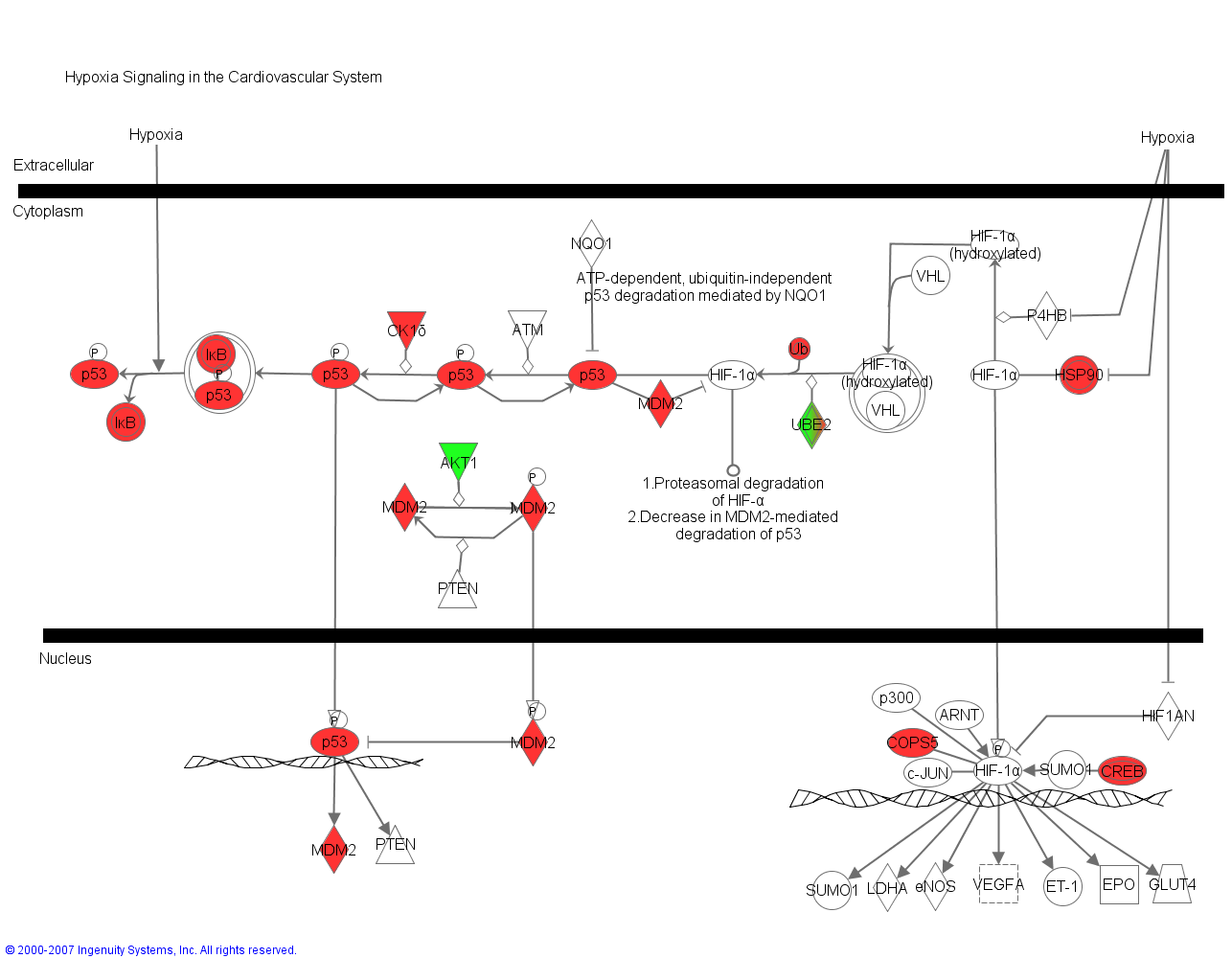


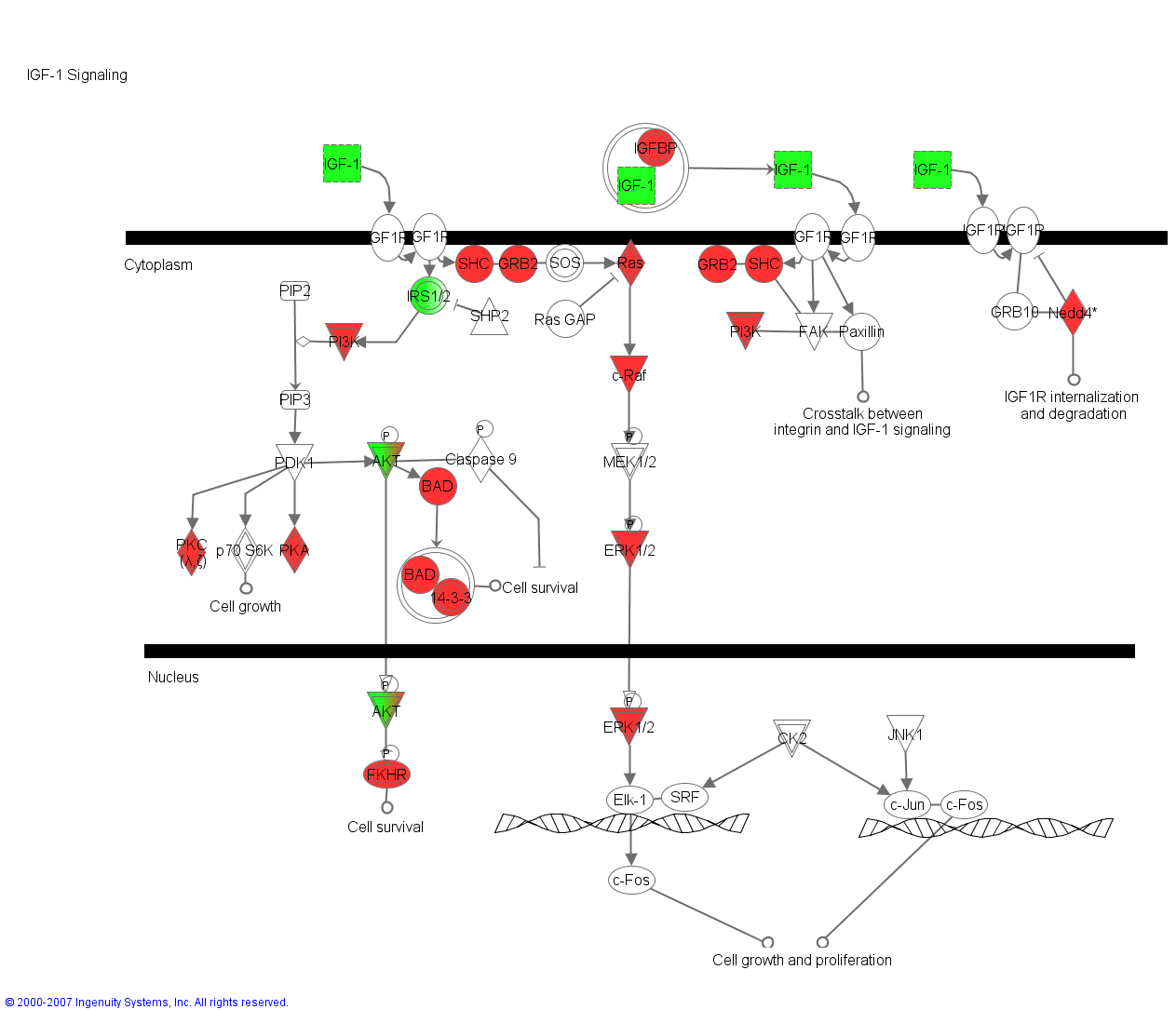


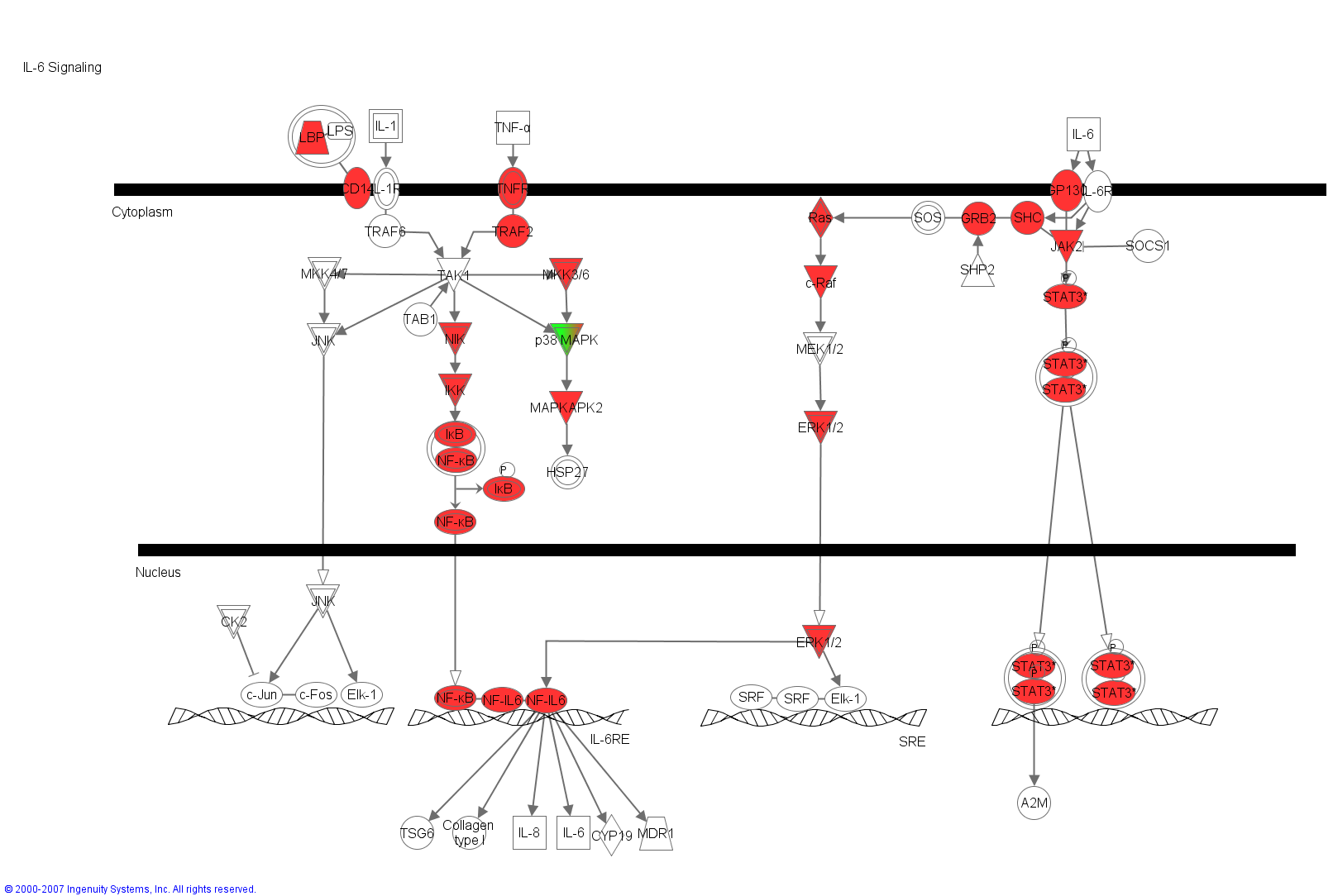


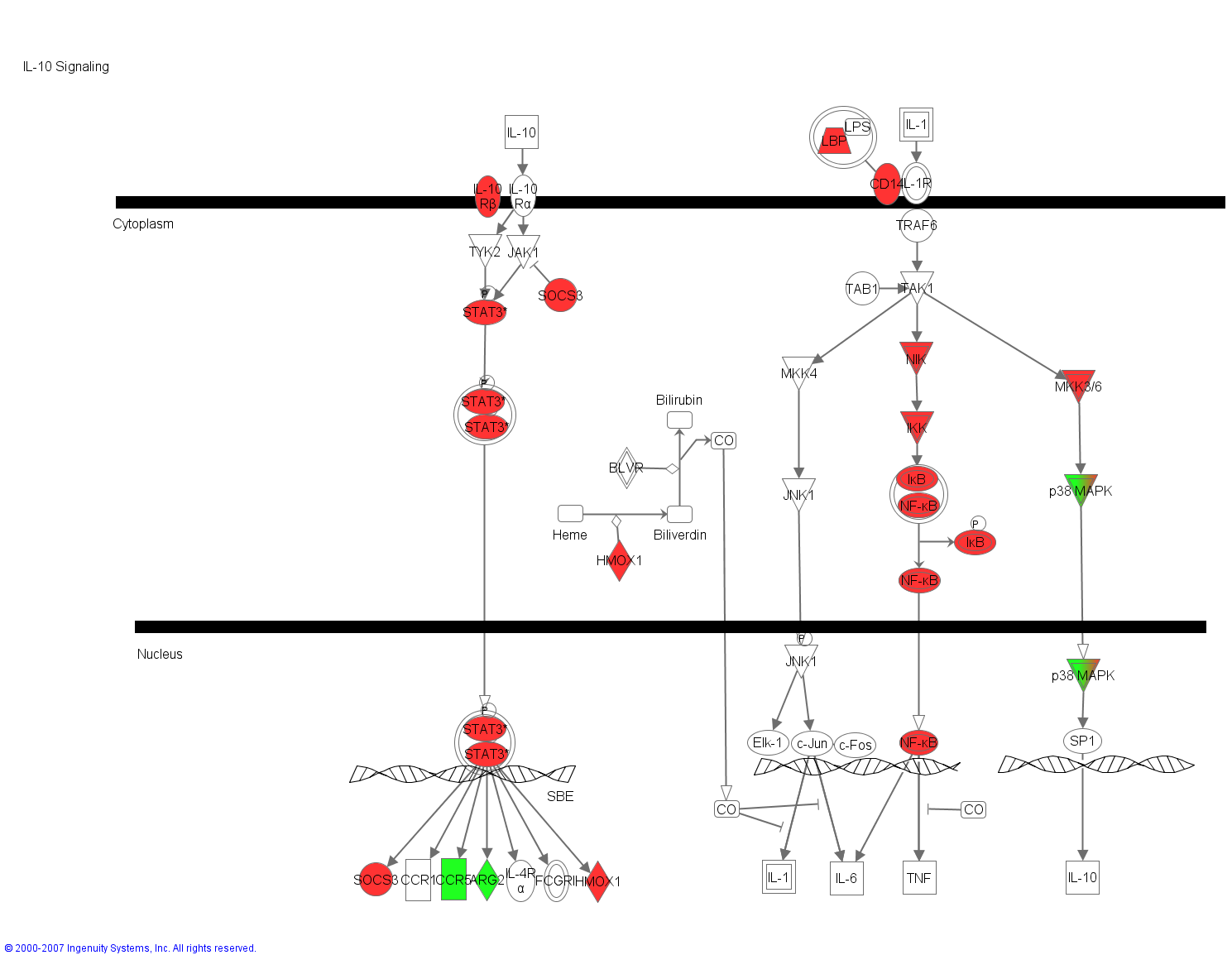


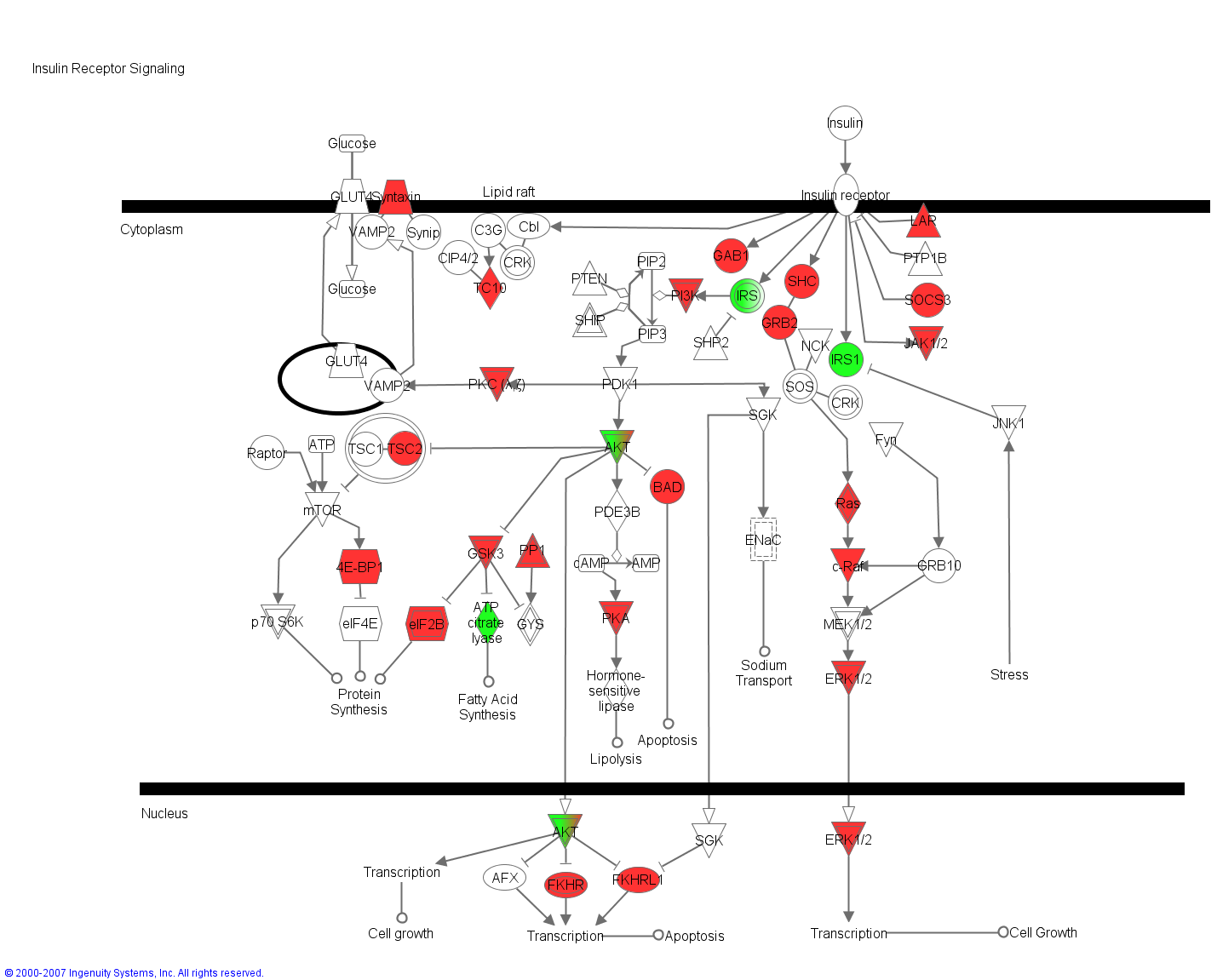


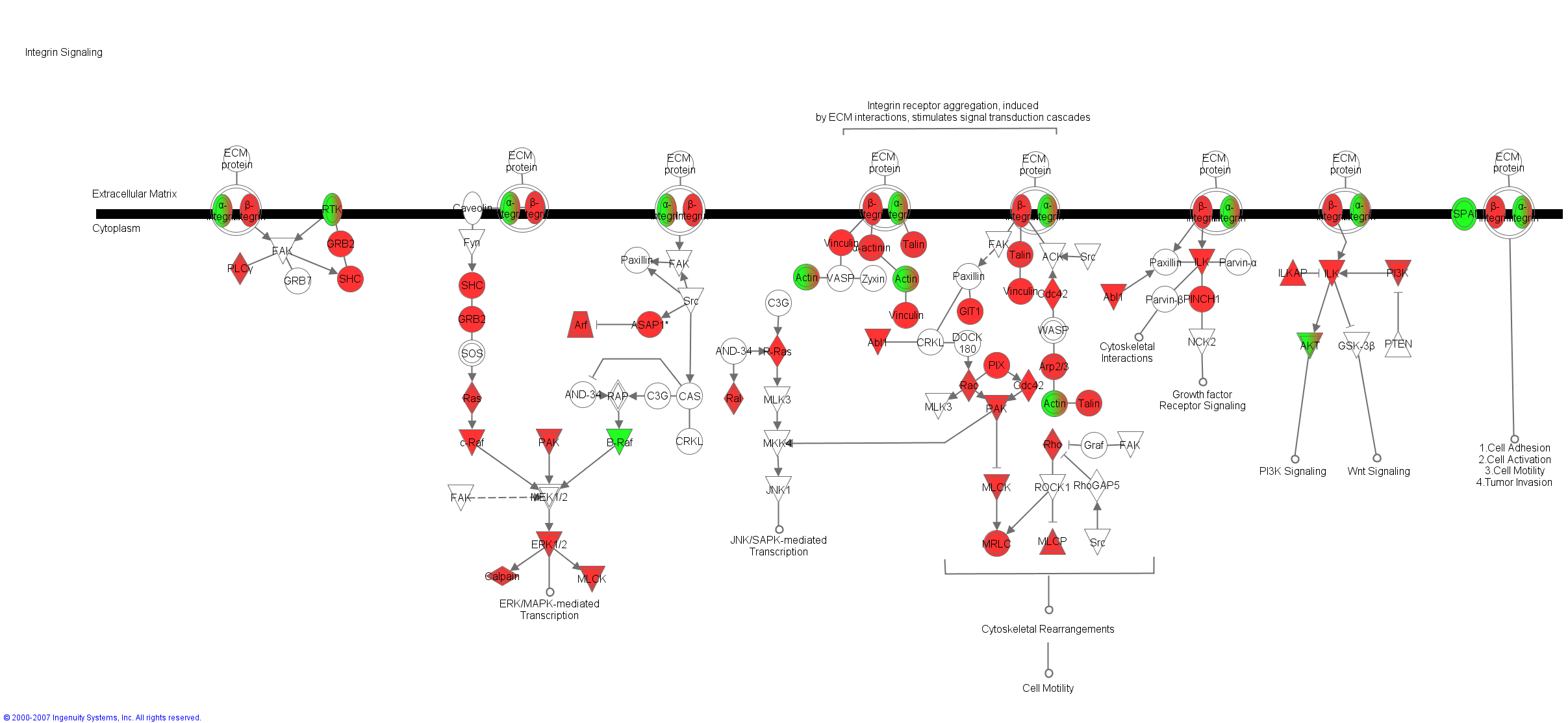


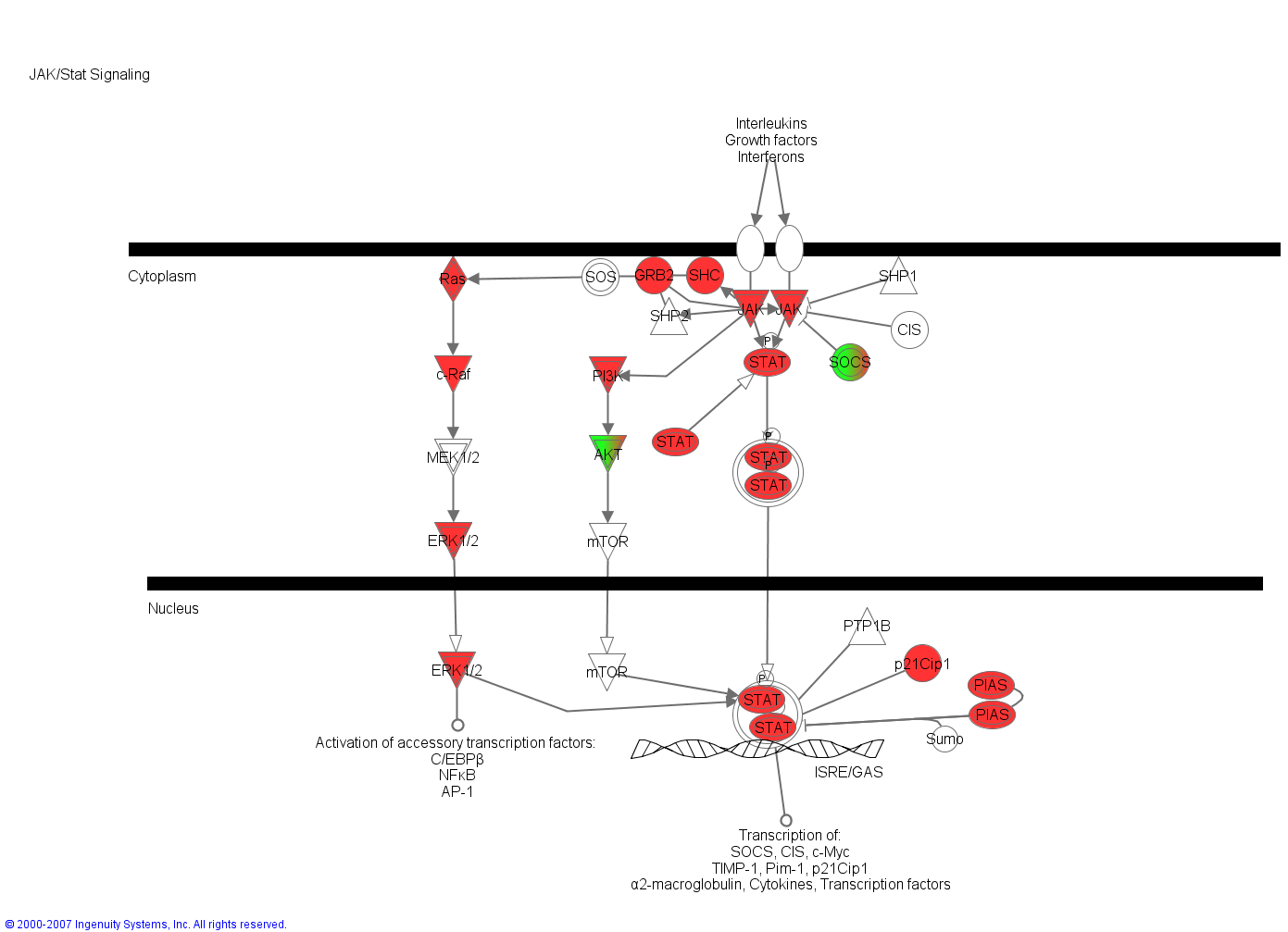


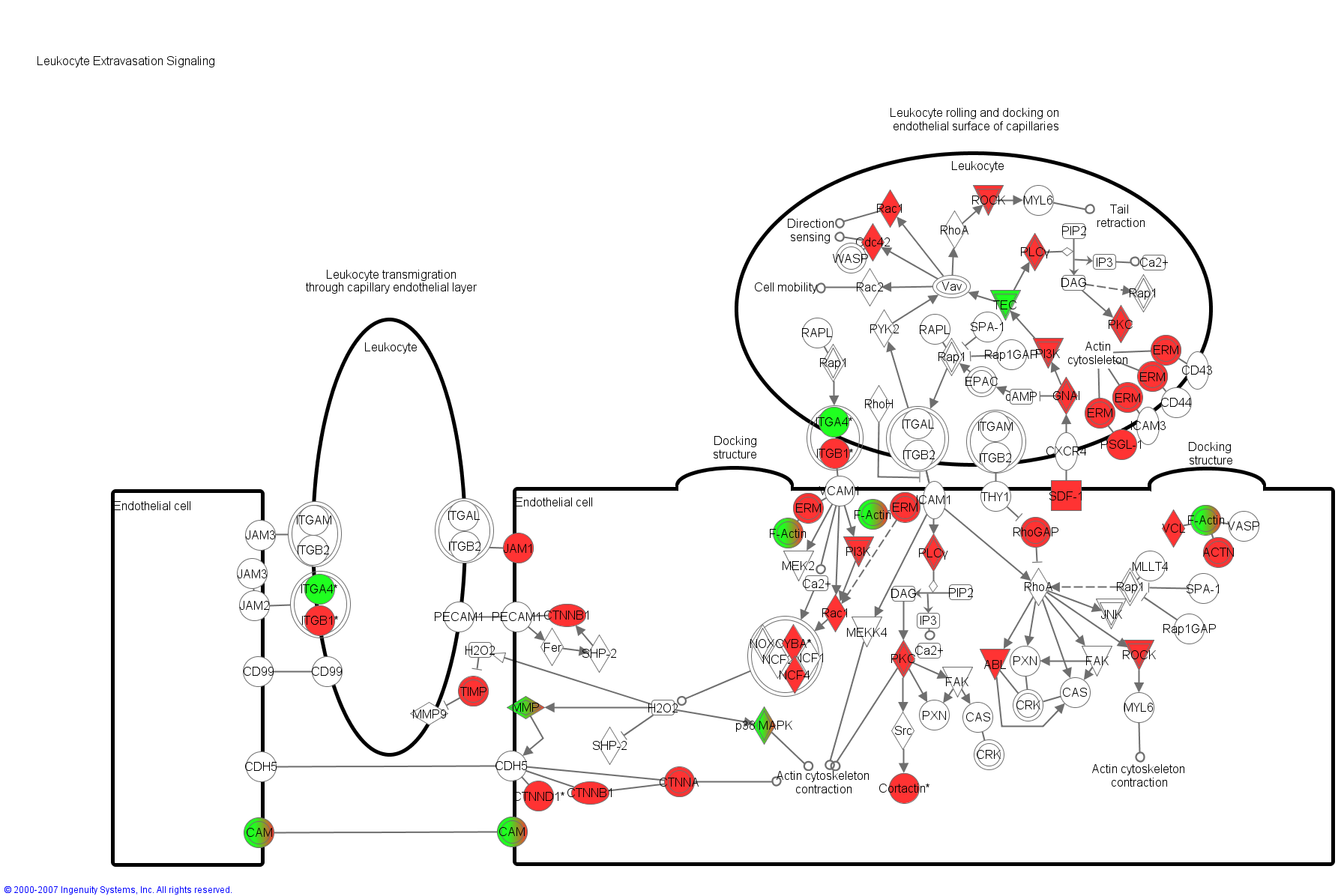


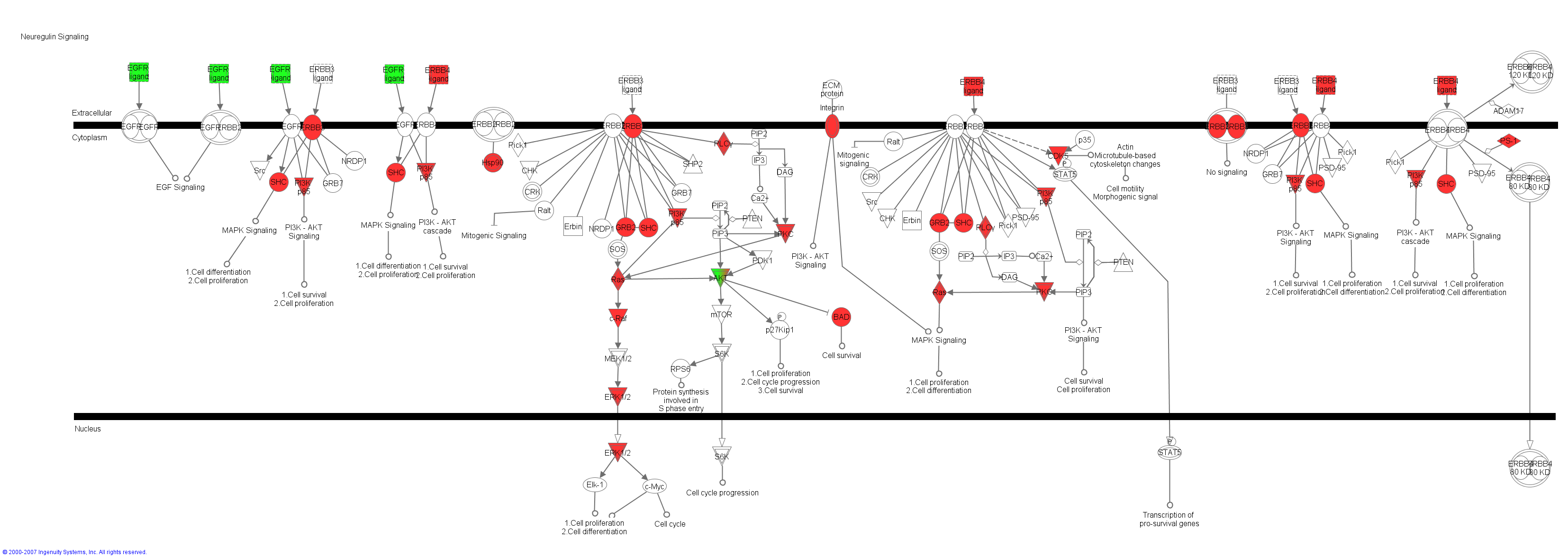


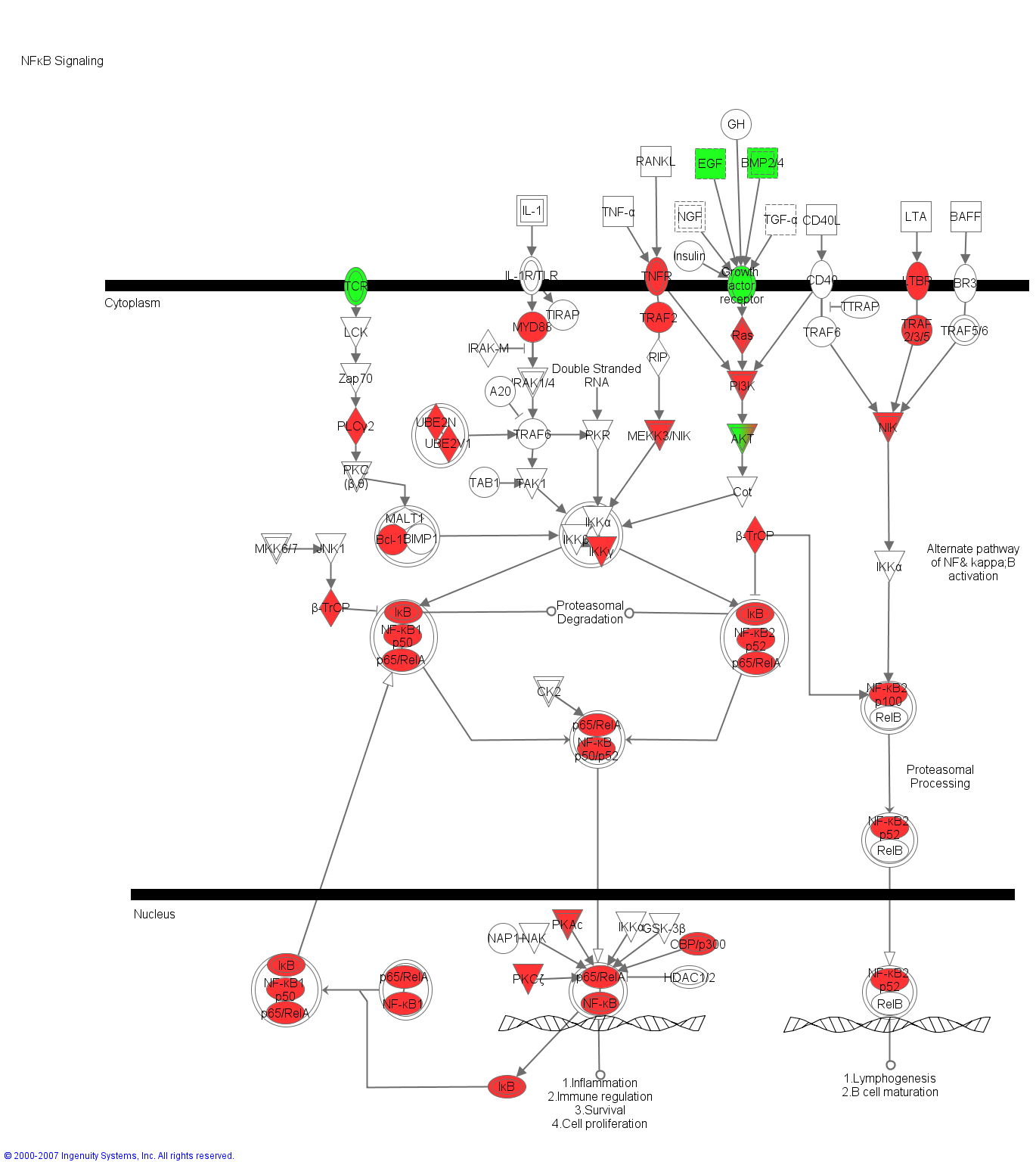


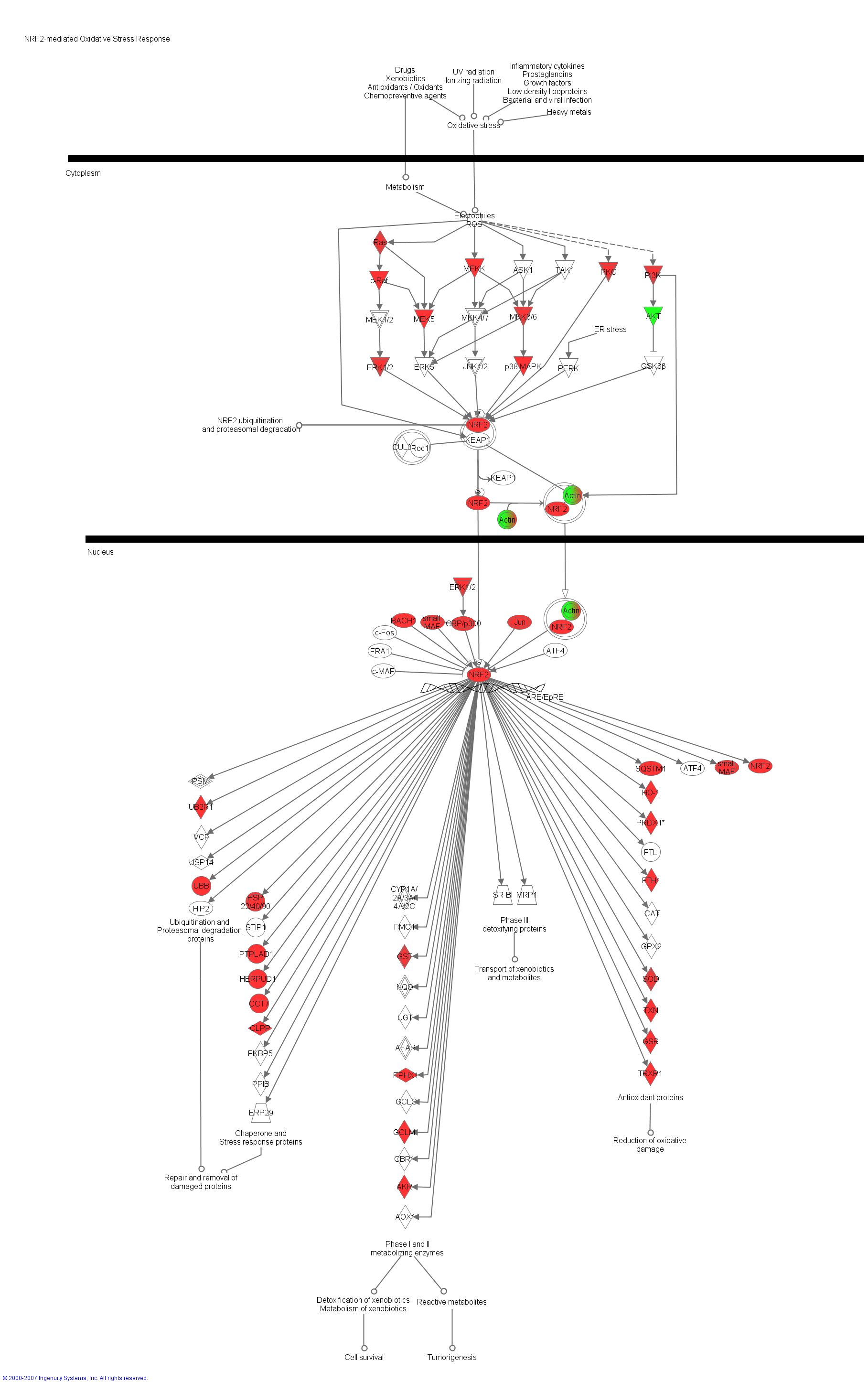


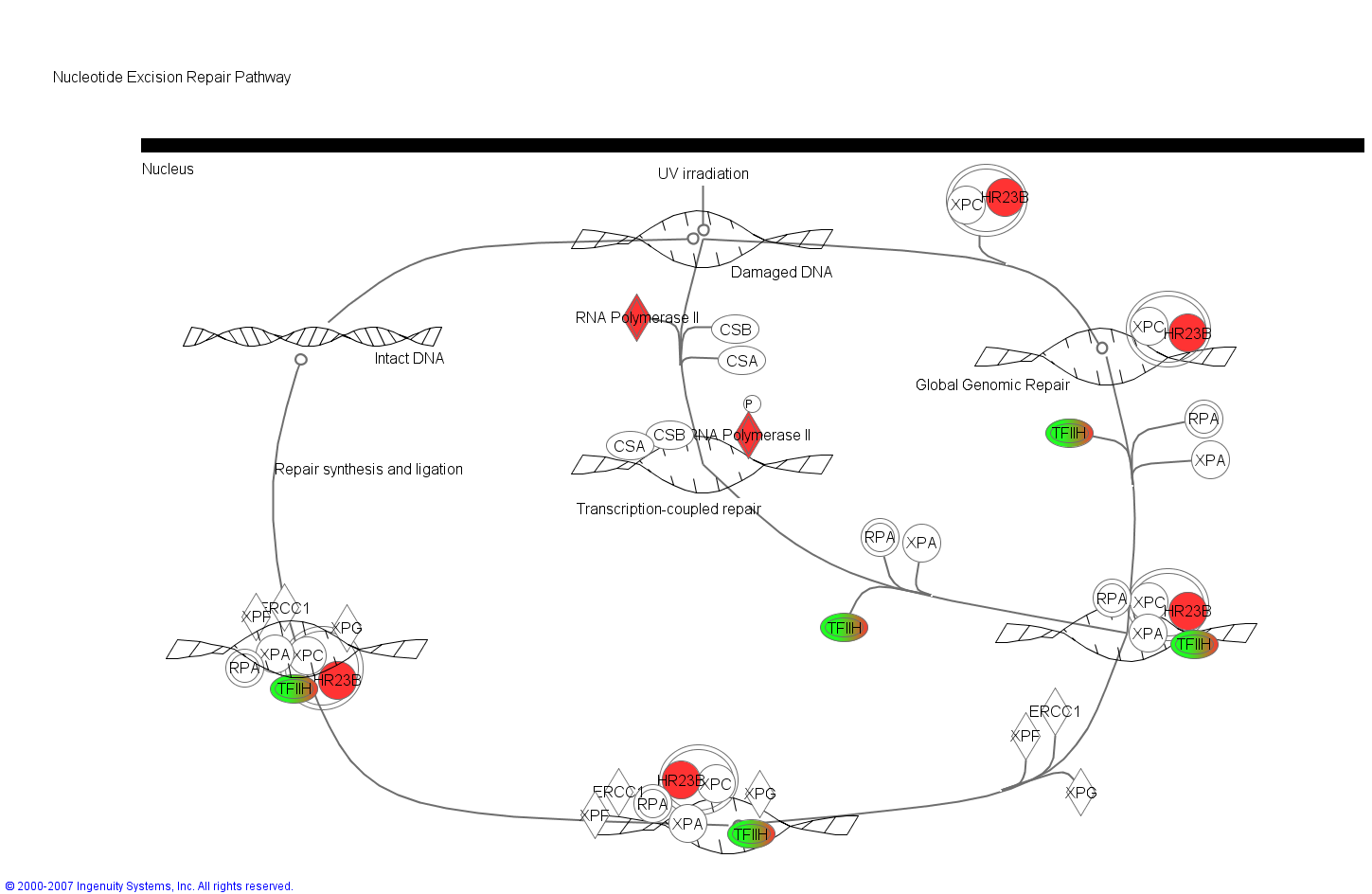


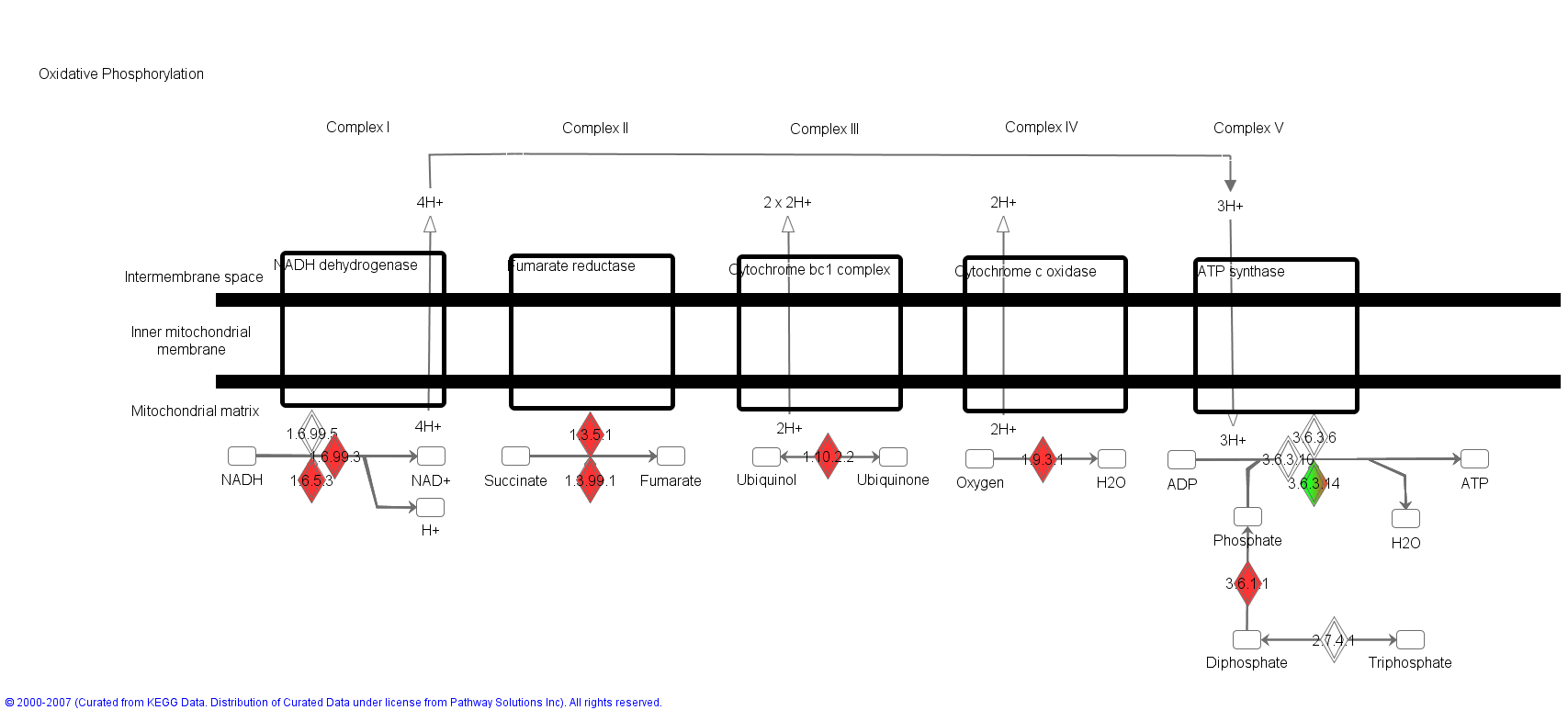


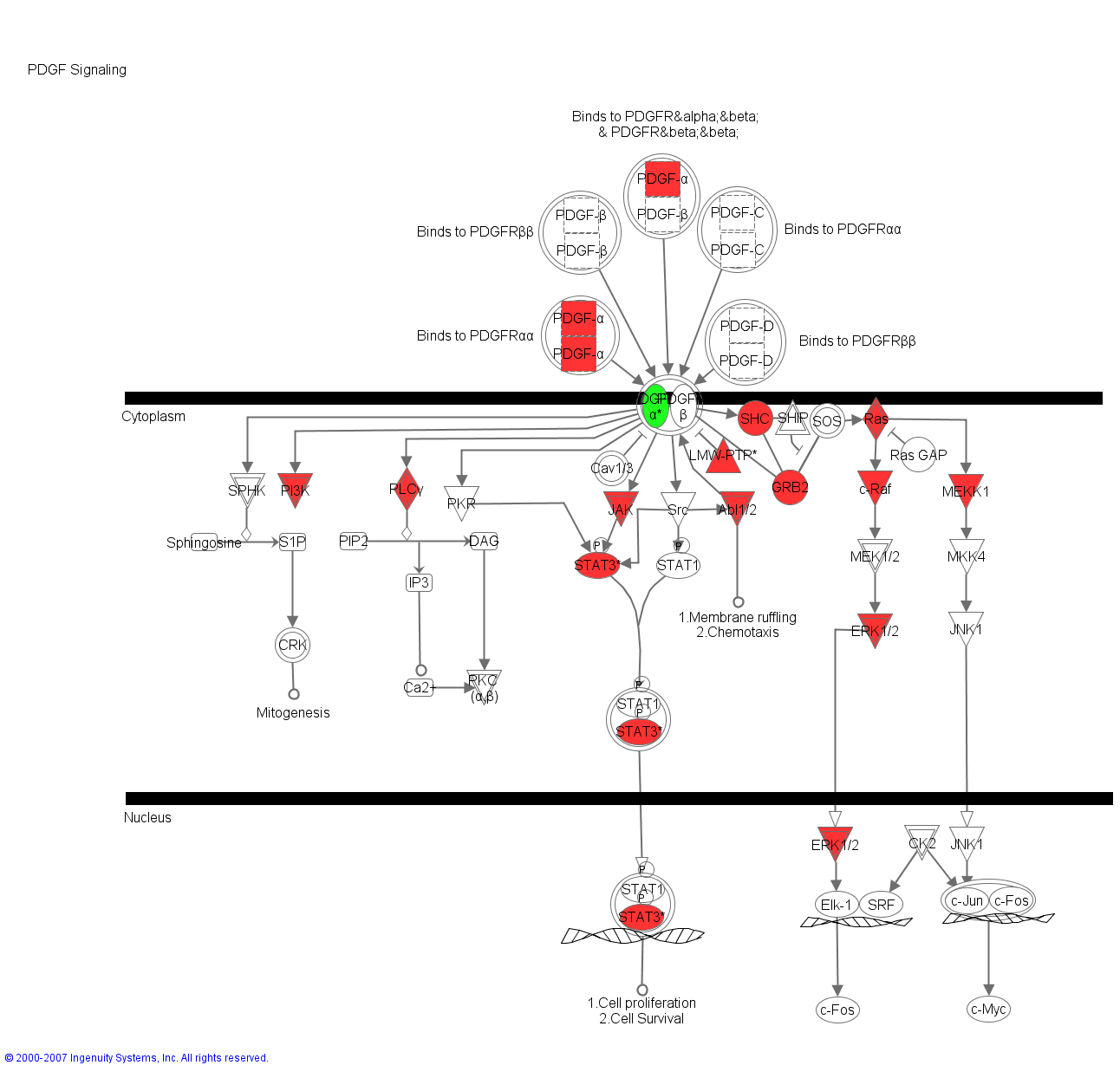


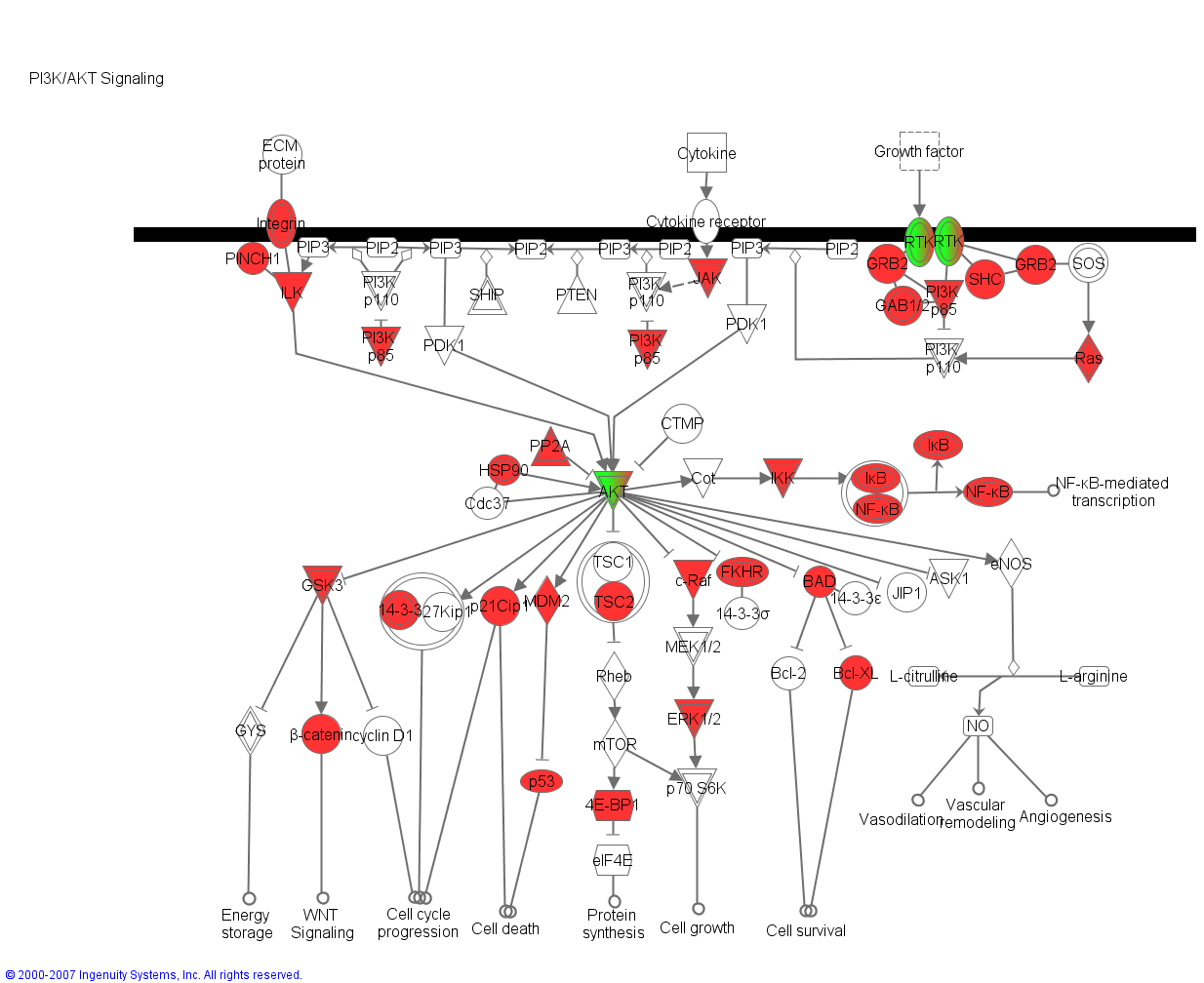


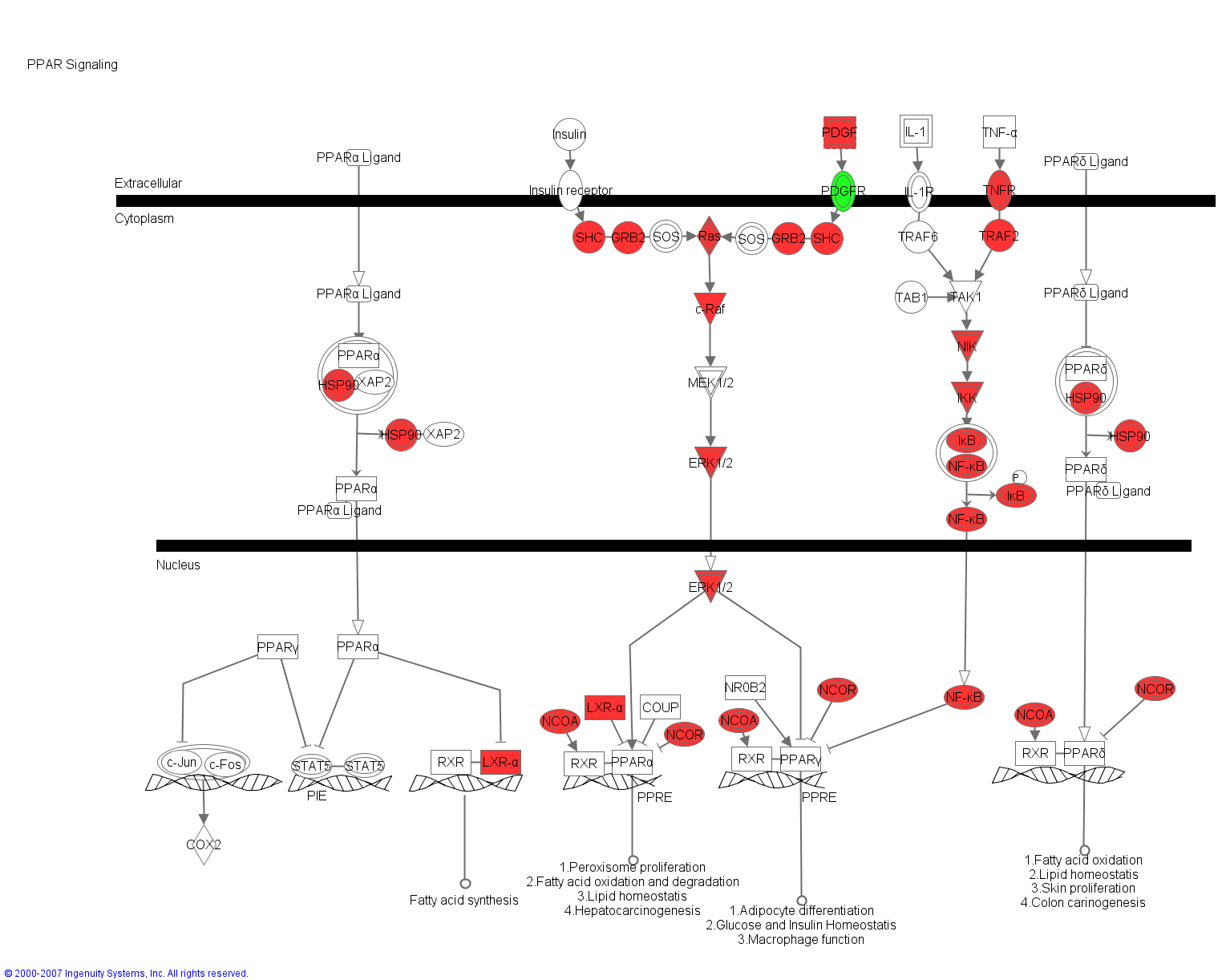


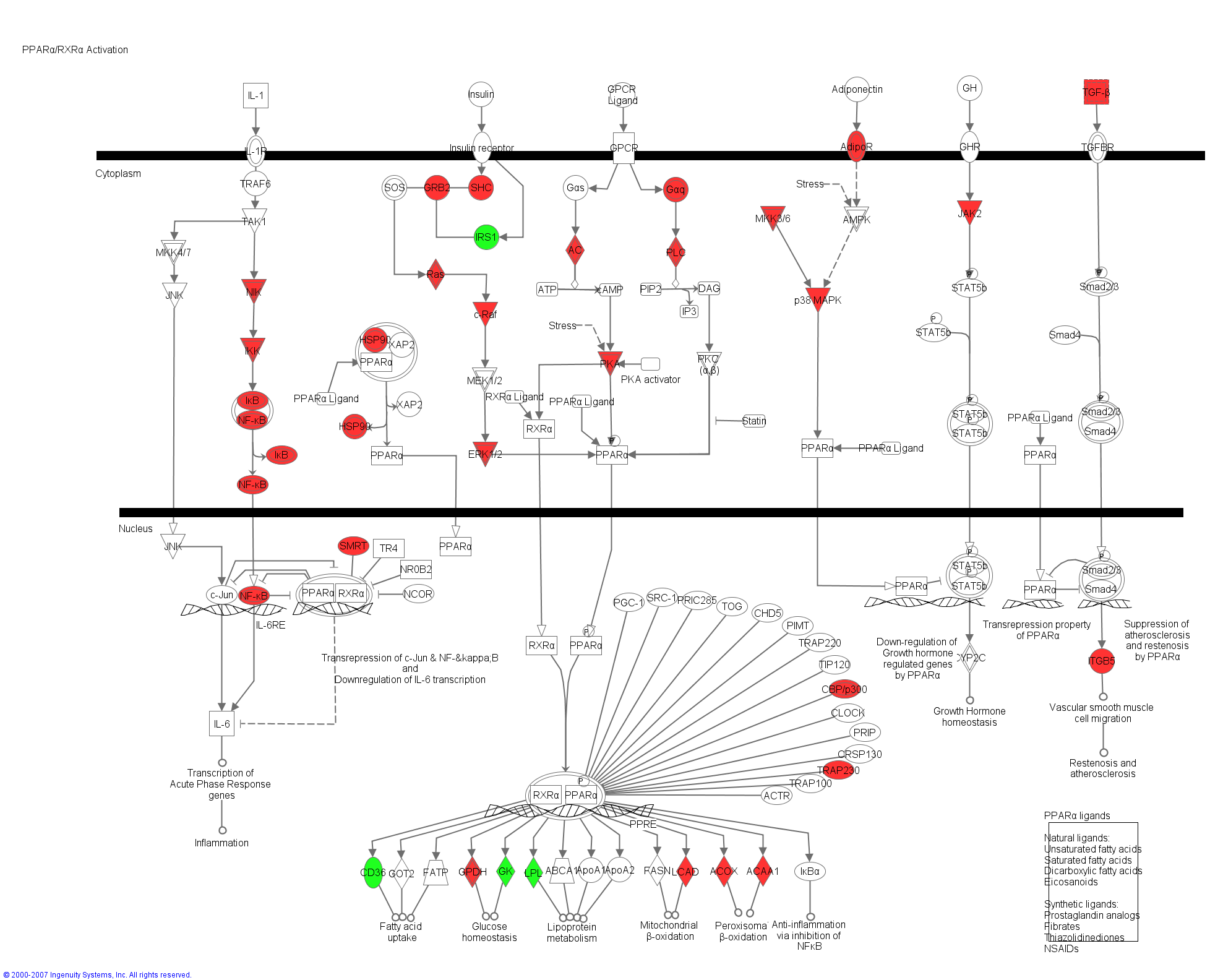


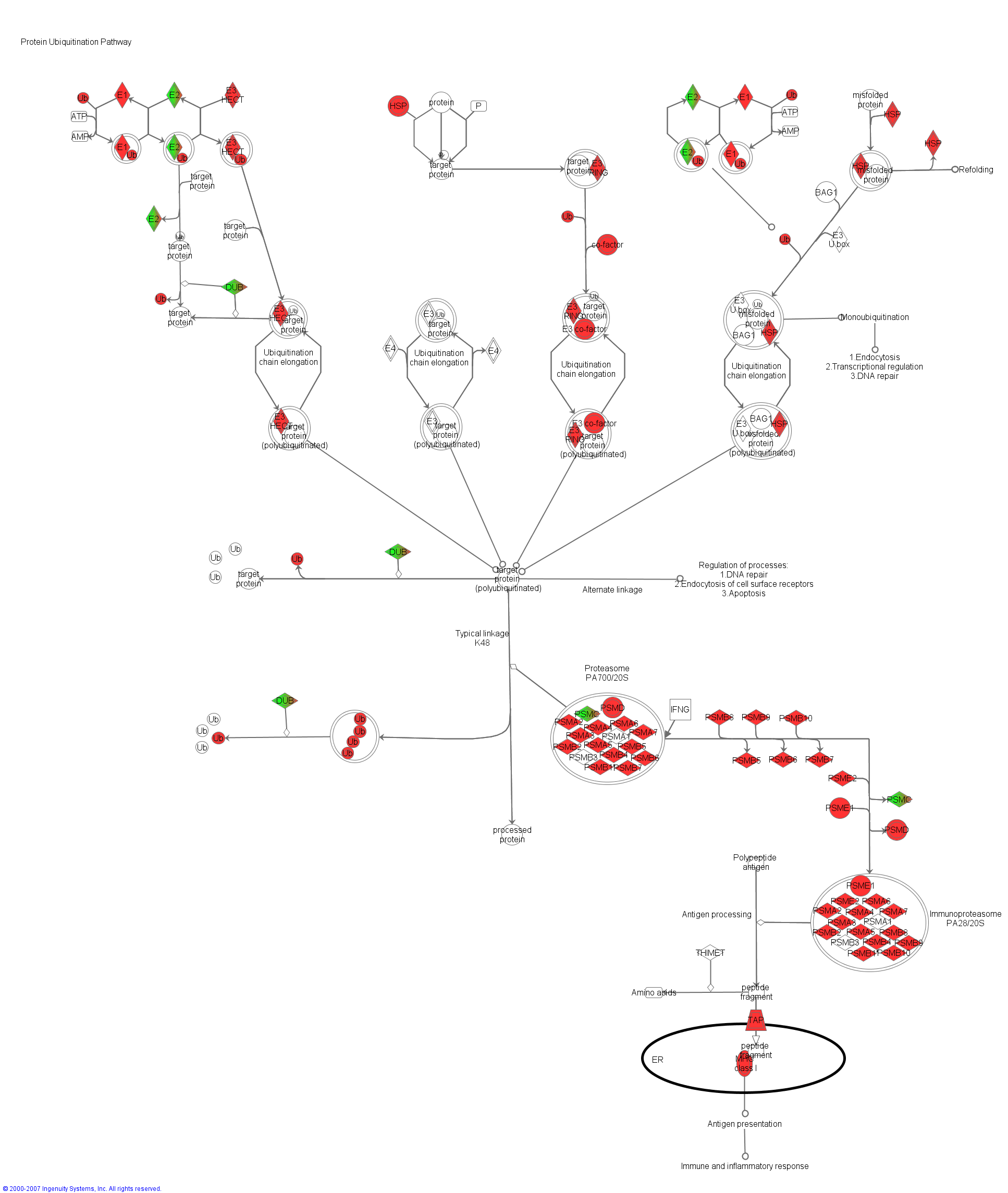


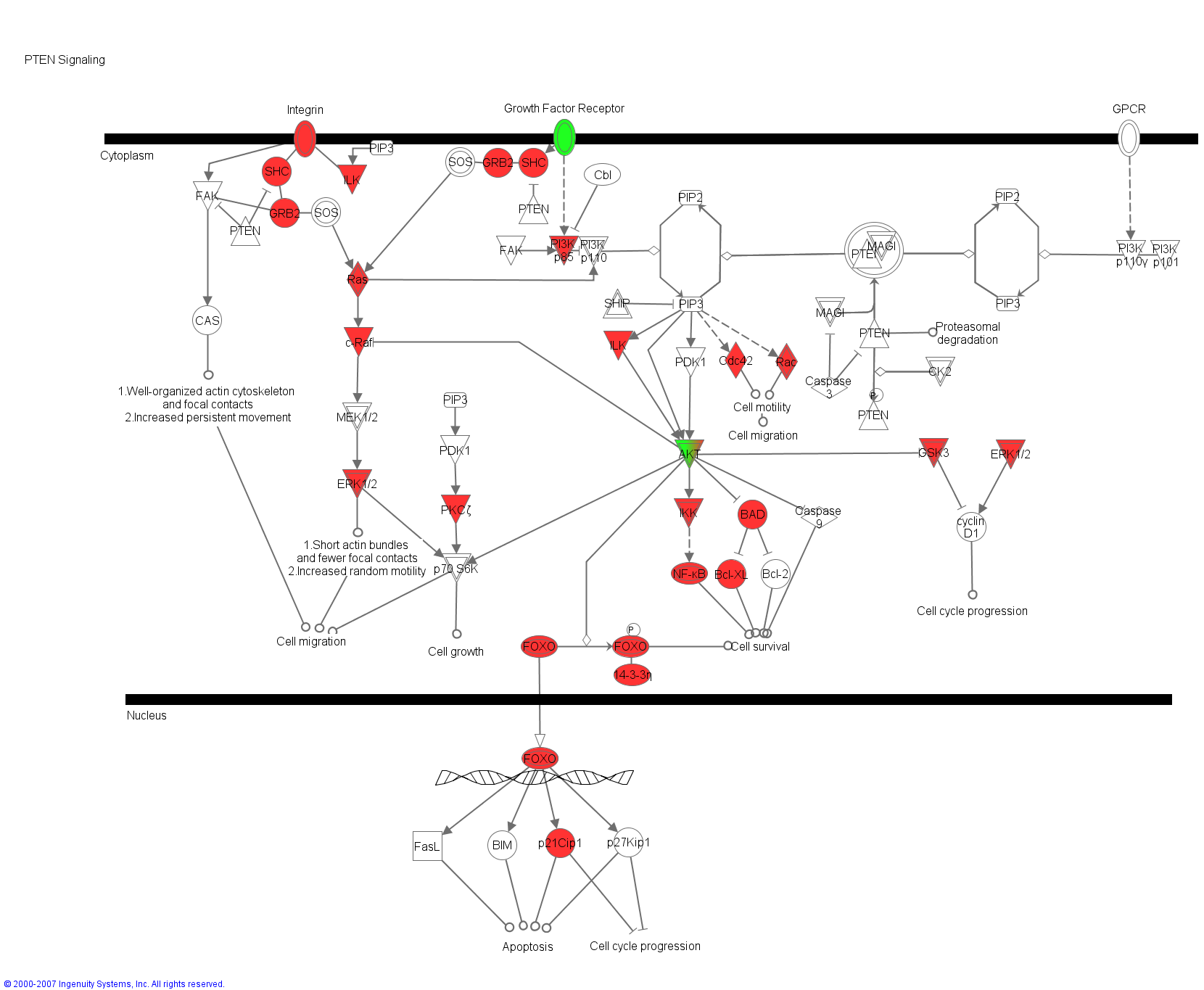


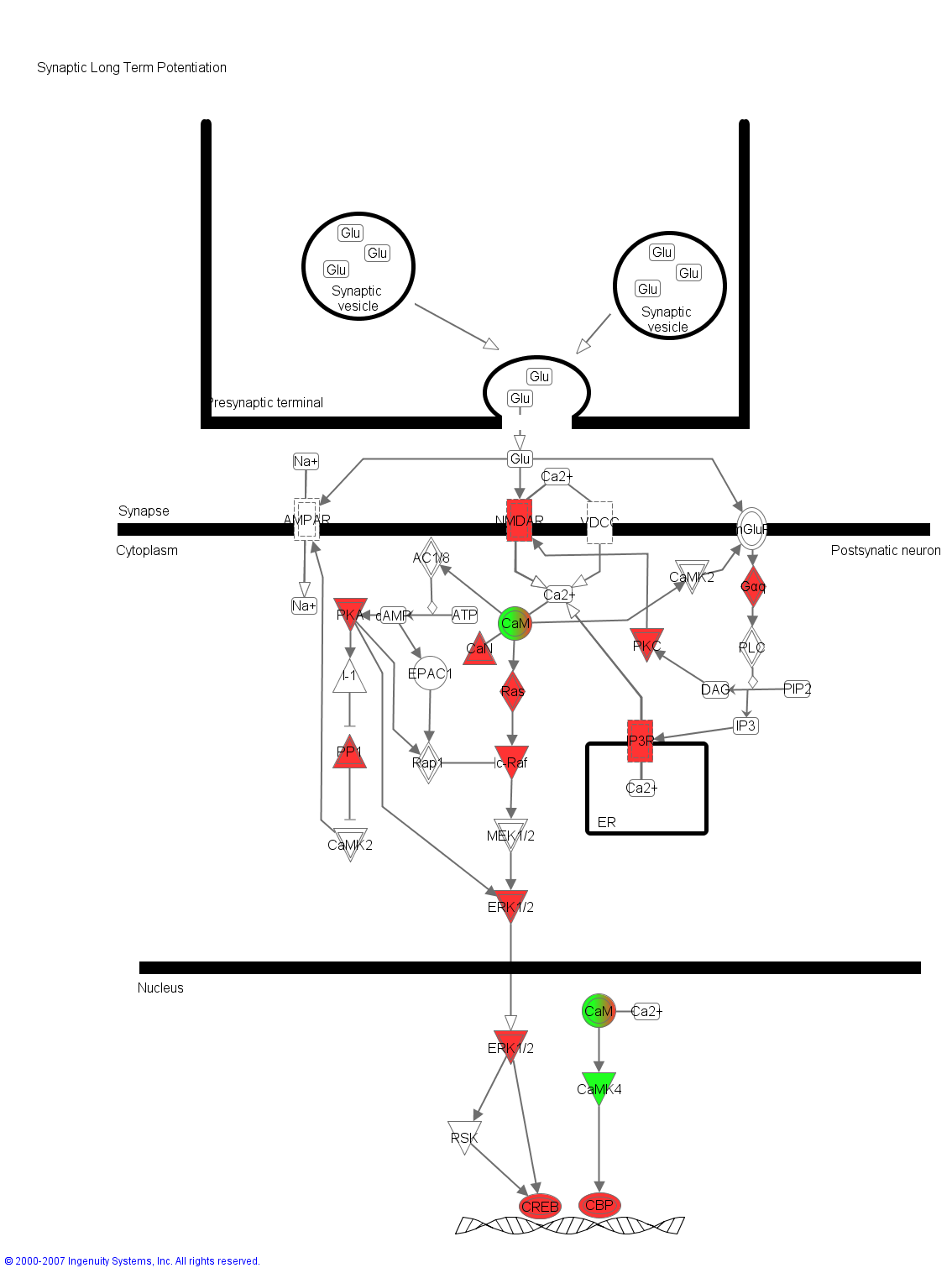


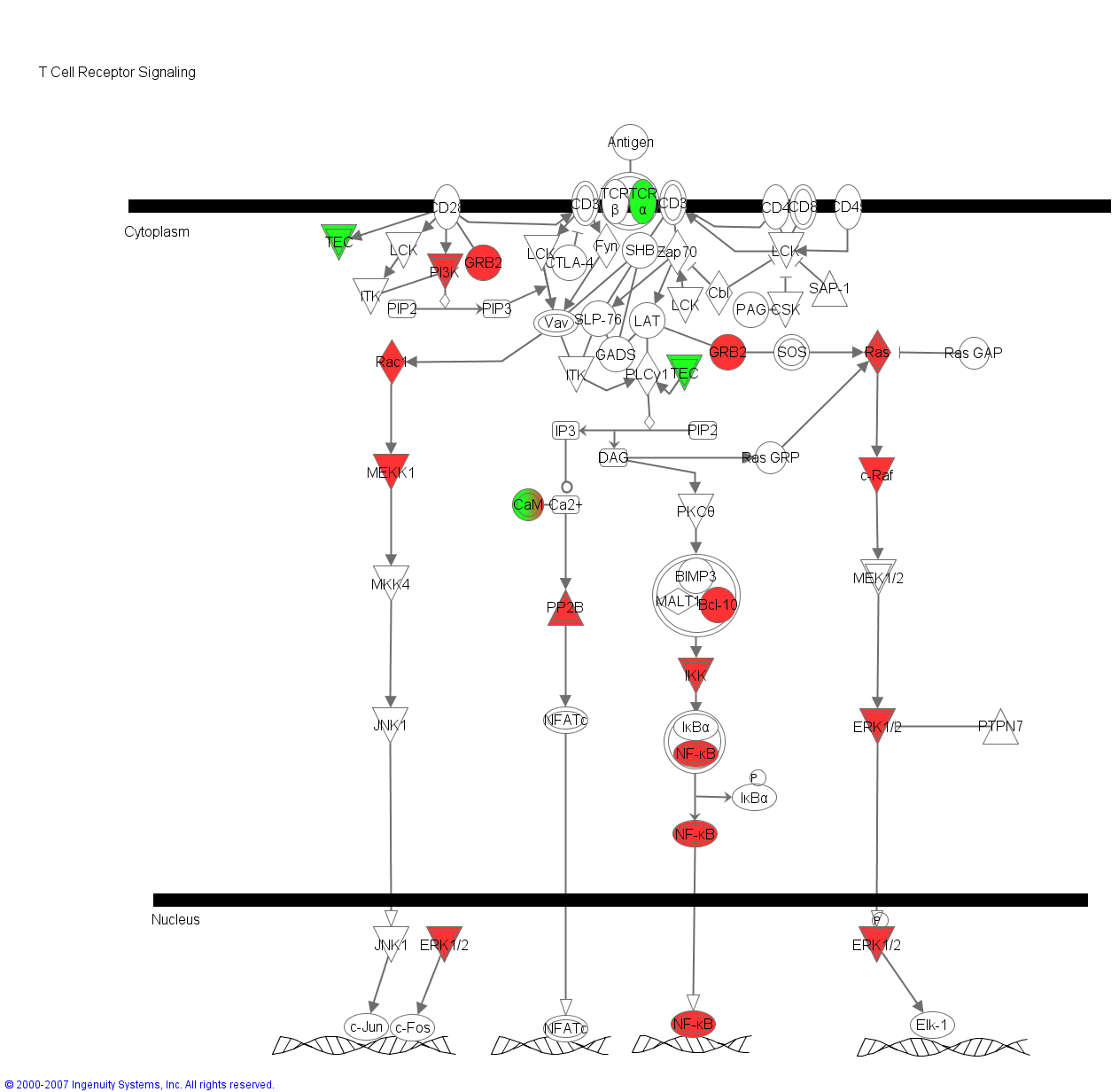


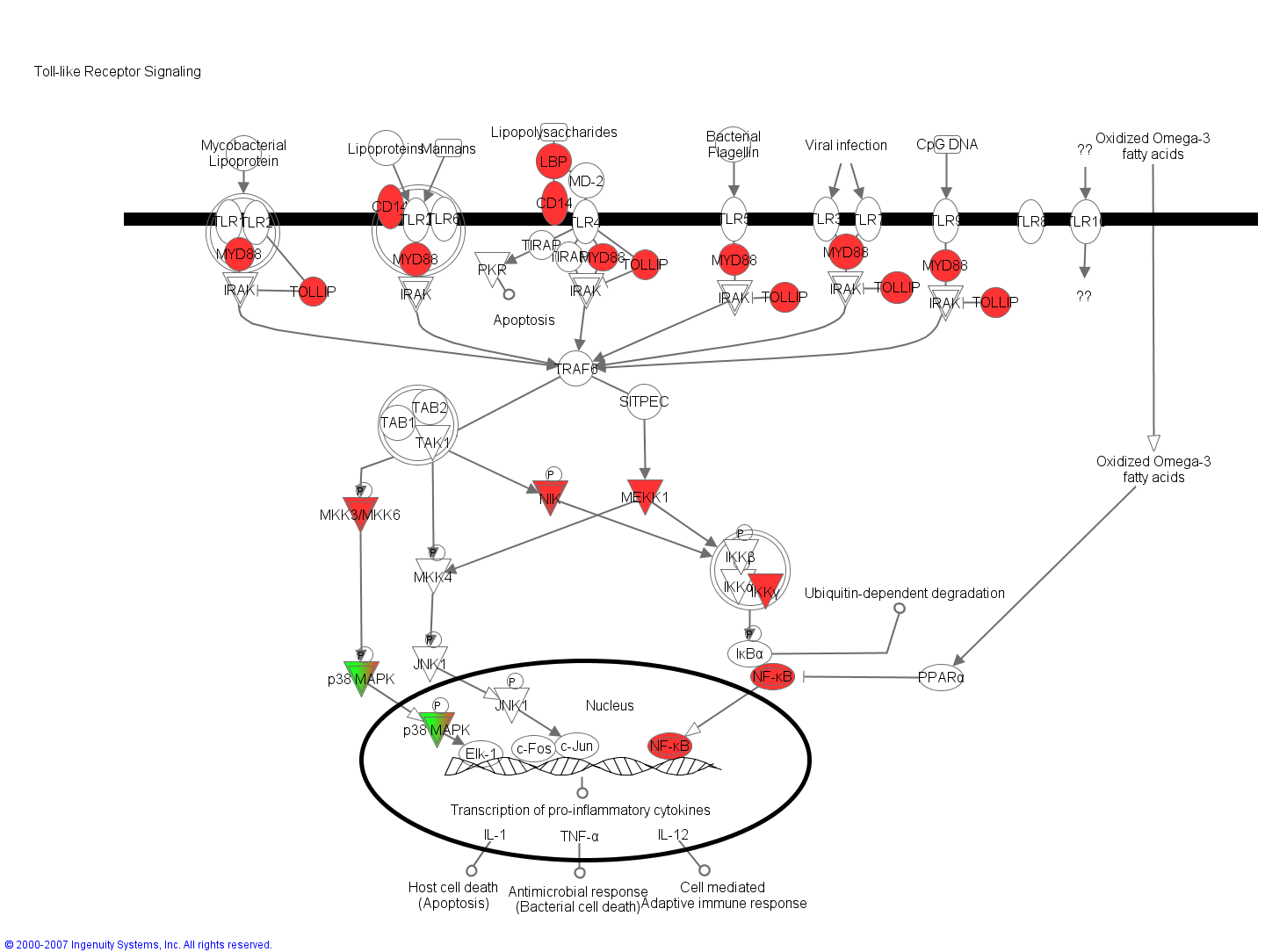


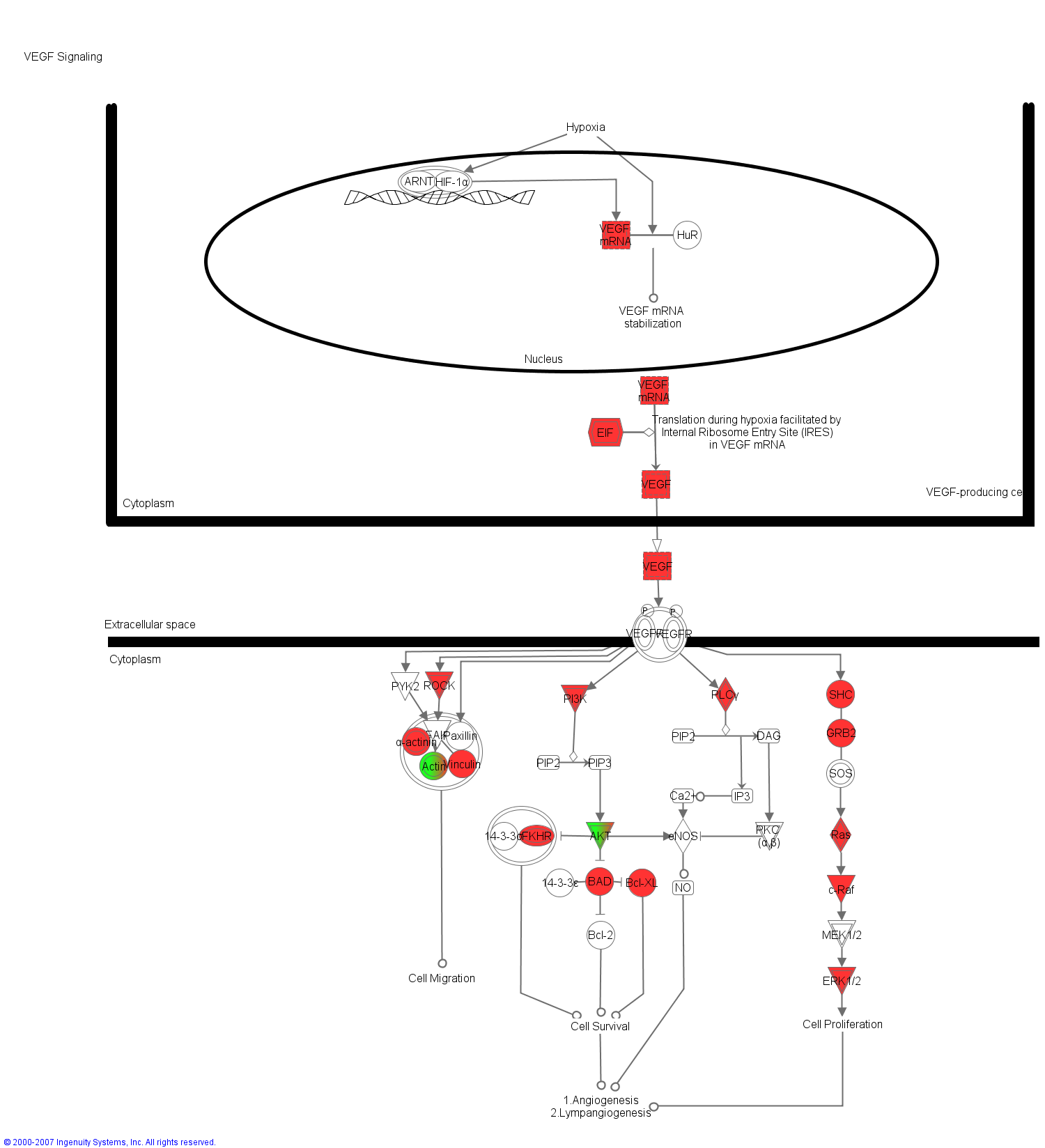


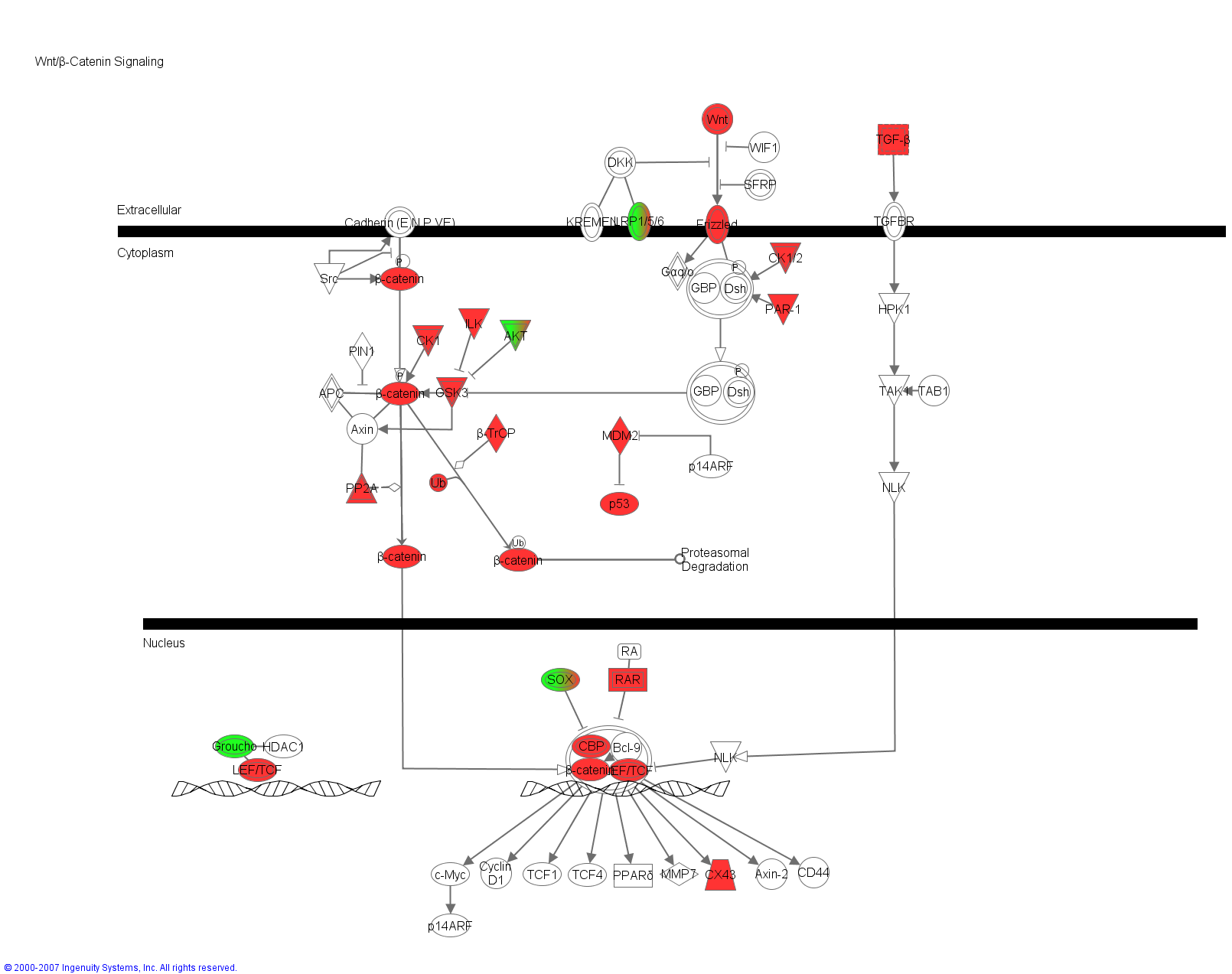


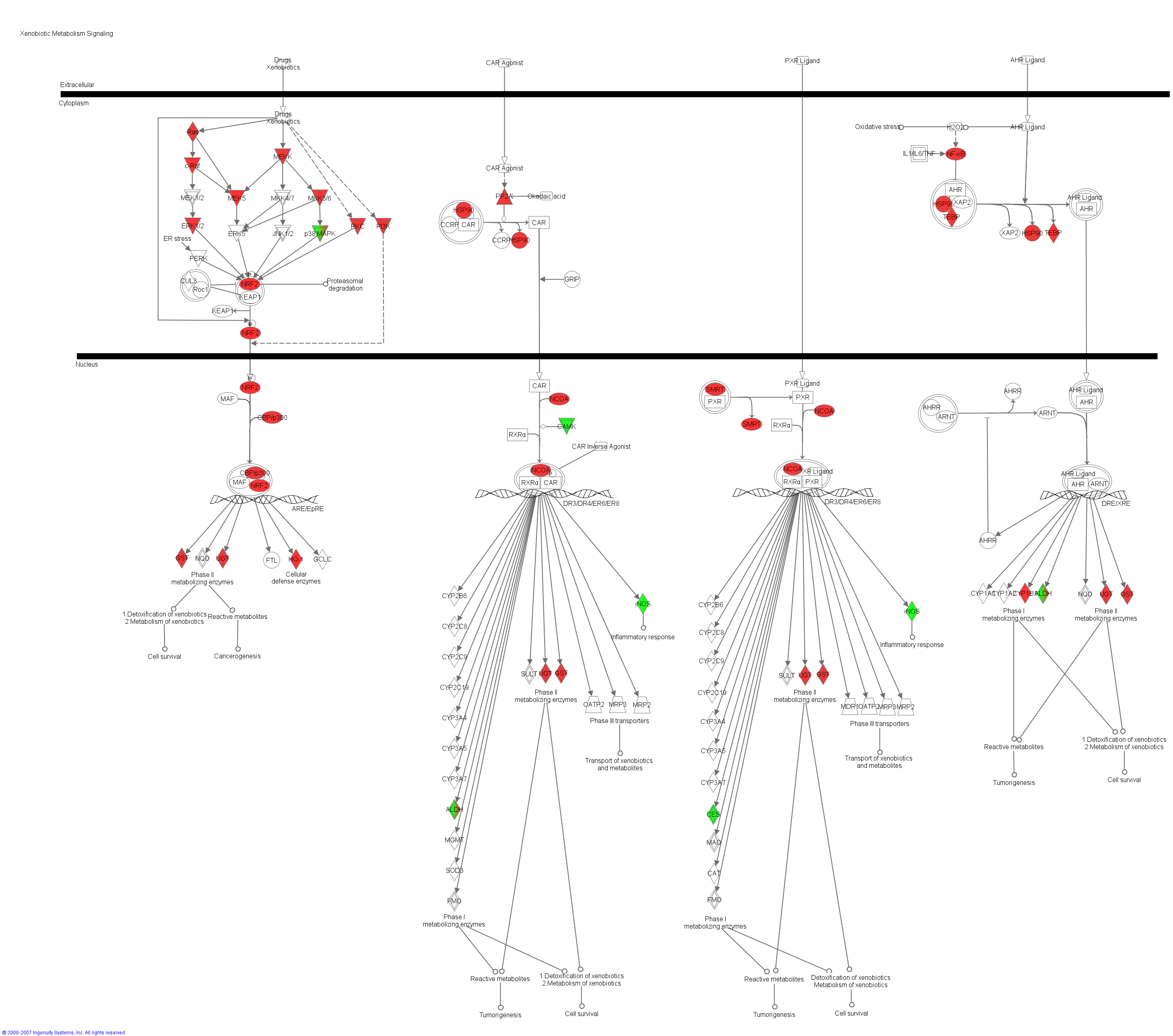

Supplement: Additional file 22 — Additional data file 22 is a Word document that contains pathway diagrams for the significant pathways associated with the Involution Gene Set. [file 1752-0509-1-56-S22.doc]
