# Supplementary material for: Gene regulatory networks in lactation: identification of global principles using bioinformatics
Source: BMC Syst Biol. 2007 Nov 27;1:56. doi: 10.1186/1752-0509-1-56 (PMC2225983; doi:10.1186/1752-0509-1-56)
Supplement: Additional file 23 — Additional data file 23 is a Word document that contains manually curated general and mammary-specific functions of the major hubs of the comprehensive lactation network in Figure 9. [file 1752-0509-1-56-S23.doc]

## Functional annotations of the major hubs of the comprehensive lactation network

Table 1.

| No. of Links | UniProt accession / UniProt ID / HGNC symbol | Name | General function (UniProt database [1]) | Mammary-specific function |
| --- | --- | --- | --- | --- |
| 21 | [PIR:P00533] / EGFR_HUMAN / EGFR | Epidermal growth factor receptor precursor | Receptor for EGF, but also for other members of the EGF family: involved in the control of cell growth and differentiation. | Required for growth and differentiation of immature MEC, and for survival of terminally differentiated MEC [2] |
| 17 | [PIR:P04181] / OAT_HUMAN / OAT | Ornithine aminotransferase, mitochondrial | Catalyzes this reaction:  L-ornithine + a 2-oxo acid = L-glutamate 5-semialdehyde + an L-amino acid | Catalyzes amino acid conversions for synthesis of milk proteins [3] |
| 14 | [PIR:P35520] / CBS_HUMAN / CBS | Cystathionine beta-synthase | Catalyzes this reaction:  L-serine + L-homocysteine = L-cystathionine + H(2)O. | None described |
| 13 | [PIR:O60674] / JAK2_HUMAN / JAK2 | Tyrosine-protein kinase JAK2 | Tyrosine kinase of the non-receptor type, involved in interleukin-3 and probably interleukin-23 signal transduction. | Prolactin- induced activation of JAK2/STAT5 pathway required for expression of milk protein genes [4, 5] |
| 12 | [PIR:P04626] / ERBB2_HUMAN / ERBB2 | Receptor tyrosine-protein kinase erbB-2 precursor | Part of a complex with EGFR and either PIK3C2A or PIK3C2B: essential component of a neuregulin-receptor complex, although neuregulins do not interact with it alone: GP30 is a potential ligand for this receptor: not activated by EGF, TGF-alpha and amphiregulin | Required for epithelial cell growth, functional differentiation, and morphogenesis of immature MECs, and the survival of terminally differentiated MECs [6] |
| 12 | [PIR:P40763] / STAT3_HUMAN / STAT3 | Signal transducer and activator of transcription 3 | Transcription factor that binds to the interleukin-6-responsive elements identified in the promoters of various acute-phase protein genes | Conditional knockout of Stat3 decreases apoptosis and dramatically delays involution [7]:  deletion of Stat3 blocks mammary gland involution: secretory epithelium remains functional in absence of lactogenic stimuli [8] |
| 12 | [PIR:P36543] / VATE_HUMAN / ATP6V1E1 | Vacuolar ATP synthase subunit E | Subunit of the peripheral V1 complex of vacuolar ATPase essential for assembly or catalytic function: V-ATPase is responsible for acidifying a variety of intracellular compartments in eukaryotic cells | None described |
| 11 | [PIR:P38398] / BRCA1_HUMAN / BRCA1 | Breast cancer type 1 susceptibility protein | Plays a central role in DNA repair by facilitating cellular response to DNA repair: involved in transcriptional regulation of P21 in response to DNA damage: required for FANCD2 targeting to sites of DNA damage: may function as a transcriptional regulator: mediates E2-dependent ubiquitination: inhibits lipid synthesis by binding to inactive phosphorylated ACACA and preventing its dephosphorylation | Suppresses activity of Stat5a: likely attenuator of growth and differentiation of MECs [9] |
| 11 | [PIR:P42229] / STA5A_HUMAN / STAT5A | Signal transducer and activator of transcription 5A | Binds to the GAS element and activates PRL-induced transcription. | Prolactin- induced activation of JAK2/STAT5 pathway required for expression of milk protein genes [4, 5] |
| 11 | [PIR:P52209] / 6PGD_HUMAN / PGD | 6-phosphogluconate dehydrogenase, decarboxylating | Catalyzes this reaction:  6-phospho-D-gluconate + NADP(+) = D-ribulose 5-phosphate + CO(2) + NADPH. | None described |
| 11 | [PIR:Q00341] / VIGLN_HUMAN / HDLBP | Vigilin | Appears to play a role in cell sterol metabolism: it may function to protect cells from over-accumulation of cholesterol | None described |
| 11 | [PIR:P35606] / COPB2_HUMAN / COPB2 | Coatomer subunit beta | This coatomer complex protein, essential for Golgi budding and vesicular trafficking, is a selective binding protein (RACK) for protein kinase C, epsilon type: it binds to Golgi membranes in a GTP-dependent manner | None described |
| 10 | [PIR:P10912] / GHR_HUMAN / GHR | Growth hormone receptor precursor | Receptor for pituitary gland growth hormone involved in regulating postnatal body growth: on ligand binding, couples to the JAK2/STAT5 pathway | Regulates transcription through JAK2/STAT5 pathway: another unknown signaling pathway is suspected during lactation [10] |
| 10 | [PIR:P29350] / PTN6_HUMAN / PTPN6 | Tyrosine-protein phosphatase non-receptor type 6 | Plays a key role in hematopoiesis: this PTPase activity may directly link growth factor receptors and other signaling proteins through protein-tyrosine phosphorylation | None described |
| 10 | [PIR:P06730] / IF4E_HUMAN / EIF4E | Eukaryotic translation initiation factor 4E | Recognizes and binds the 7-methylguanosine-containing mRNA cap during an early step in the initiation of protein synthesis and facilitates ribosome binding by inducing the unwinding of the mRNAs secondary structures | Regulates the proliferation of MECs [11]: elevated eIF-4E expression during lactation may be related to increased translation of certain mRNA or the acceleration of overall protein synthesis [12] |
| 10 | [PIR:P49591] / SYSC_HUMAN / SARS | Seryl-tRNA synthetase, cytoplasmic | Catalyzes this reaction:  ATP + L-serine + tRNA(Ser) = AMP + diphosphate + L-seryl-tRNA(Ser) | None described |
| 10 | [PIR:Q15437] /  SC23B_HUMAN / SEC23B | Protein transport protein Sec23B | Component of the COPII coat, that covers ER-derived vesicles involved in transport from the endoplasmic reticulum to the Golgi apparatus | None described |
| 10 | [PIR:O14929] / HAT1_HUMAN / HAT1 | Histone acetyltransferase type B catalytic subunit | May play a role in telomeric silencing: acetylates soluble but not nucleosomal H4 at 'Lys-5' and 'Lys-12' and acetylates histone H2A at 'Lys-5': HAT1 has intrinsic substrate specificity that modifies lysine in recognition sequence GXGKXG | None described |
| 9 | [PIR:O14543] /  SOCS3_HUMAN / SOCS3 | Suppressor of cytokine signaling 3 | SOCS3 is involved in negative regulation of cytokines that signal through the JAK/STAT pathway: inhibits cytokine signal transduction by binding to tyrosine kinase receptors including gp130, LIF, erythropoietin, insulin, IL12, GCSF and leptin receptors: binding to JAK2 inhibits its kinase activity | Prolactin-induced SOCS3 promotes apoptosis of stromal adipocytes during early pregnancy and of MECs during involution[13] |
| 9 | [PIR:P35568] / IRS1_HUMAN / IRS1 | Insulin receptor substrate 1 | When phosphorylated by the insulin receptor binds specifically to various cellular proteins containing SH2 domains such as phosphatidylinositol 3-kinase p85 subunit or GRB2: activates phosphatidylinositol 3-kinase when bound to the regulatory p85 subunit | In addition to being a signaling intermediate for the insulin receptor, IRS-1 is also responsive to Prolactin [14]: IRS-1 protein levels change by 200-fold during mammary gland development, but mRNA levels differ by less than 2-fold [15] |
| 9 | [PIR:O15524] / SOCS1_HUMAN / SOCS1 | Suppressor of cytokine signaling 1 | SOCS1 is involved in negative regulation of cytokines that signal through the JAK/STAT3 pathway: through binding to JAKs, inhibits their kinase activity: appears to be a major regulator of signaling by interleukin 6 (IL6) and leukemia inhibitory factor (LIF): regulates interferon-gamma mediated sensory neuron survival: implicated, through SOCS box binding, in ubiquitin-dependent protein degradation | SOCS1 is a negative regulator of prolactin signaling: SOCS1 is required for the prevention of lactation prior to parturition [16] |
| 9 | [PIR:Q15796] / SMAD2_HUMAN / SMAD2 | Mothers against decapentaplegic homolog 2 | Transcriptional modulator activated by TGF-beta and activin type 1 receptor kinase | Mammary development is controlled by many growth factors, one of which is activin, a member of the TGF-beta superfamily: low levels of TGF-beta may be required during lactation to avoid untimely apoptosis [17]: SMAD2 is one of several intracellular transducers of the activin signal |
| 9 | [PIR:P61960] / UFM1_HUMAN / UFM1 | Ubiquitin-fold modifier 1 precursor | Ubiquitin-like modifier protein which binds to a number of as yet unidentified target proteins | None described |
| 8 | [PIR:P06213] / INSR_HUMAN / INSR | Insulin receptor precursor | This receptor binds insulin and has a tyrosine-protein kinase activity: mediates the metabolic functions of insulin: binding to insulin stimulates association of the receptor with downstream mediators including IRS1 and phosphatidylinositol 3'-kinase (PI3K) | While adipose tissue becomes insulin resistant during late pregnancy, the mammary gland becomes insulin sensitive. This increased sensitivity for insulin is due to increased kinase activity of the insulin receptor which occurs during late pregnancy and lactation [18]. |
| 8 | [PIR:O75821] / IF34_HUMAN / EIF3S4 | Eukaryotic translation initiation factor 3 subunit 4 | Binds to the 40S ribosome and promotes the binding of methionyl-tRNAi and mRNA: this subunit binds to the 18S rRNA. | None described. |
| 8 | [PIR:P17676] / CEBPB_HUMAN / CEBPB | CCAAT/enhancer-binding protein beta | Important transcriptional activator in the regulation of genes involved in immune and inflammatory responses: specifically binds to an IL-1 response element in the IL-6 gene | Transcription factor involved in regulation of milk protein genes.[19]  Transcriptional activator required for ductal morphogenesis, lobuloalveolar development, and functional differentiation of MECs.[20] |
| 8 | [PIR:P04150] / GCR_HUMAN / NR3C1 | Glucocorticoid receptor | Receptor for glucocorticoids (GC): has a dual mode of action: as a transcription factor that binds to glucocorticoid response elements (GRE) and as a modulator of other transcription factors: affects inflammatory responses, cellular proliferation and differentiation in target tissues: could act as a coactivator for STAT5-dependent transcription upon growth hormone | Influences cell proliferation during lobuloalveolar development but is not essential for lactation or for expression of milk protein genes. [21] |

# References

1. **The Universal Protein Resource (UniProt).** *Nucleic Acids Res* 2007, **35:**D193-197.

2. Darcy KM, Wohlhueter AL, Zangani D, Vaughan MM, Russell JA, Masso-Welch PA, Varela LM, Shoemaker SF, Horn E, Lee PP, et al: **Selective changes in EGF receptor expression and function during the proliferation, differentiation and apoptosis of mammary epithelial cells.** *Eur J Cell Biol* 1999, **78:**511-523.

3. Mezl VA, Knox WE: **Metabolism of arginine in lactating rat mammary gland.** *Biochem J* 1977, **166:**105-113.

4. Schmitt-Ney M, Doppler W, Ball RK, Groner B: **Beta-casein gene promoter activity is regulated by the hormone-mediated relief of transcriptional repression and a mammary-gland-specific nuclear factor.** *Mol Cell Biol* 1991, **11:**3745-3755.

5. Gouilleux F, Wakao H, Mundt M, Groner B: **Prolactin induces phosphorylation of Tyr694 of Stat5 (MGF), a prerequisite for DNA binding and induction of transcription.** *Embo J* 1994, **13:**4361-4369.

6. Darcy KM, Zangani D, Wohlhueter AL, Huang RY, Vaughan MM, Russell JA, Ip MM: **Changes in ErbB2 (her-2/neu), ErbB3, and ErbB4 during growth, differentiation, and apoptosis of normal rat mammary epithelial cells.** *J Histochem Cytochem* 2000, **48:**63-80.

7. Chapman RS, Lourenco PC, Tonner E, Flint DJ, Selbert S, Takeda K, Akira S, Clarke AR, Watson CJ: **Suppression of epithelial apoptosis and delayed mammary gland involution in mice with a conditional knockout of Stat3.** *Genes Dev* 1999, **13:**2604-2616.

8. Humphreys RC, Bierie B, Zhao L, Raz R, Levy D, Hennighausen L: **Deletion of Stat3 blocks mammary gland involution and extends functional competence of the secretory epithelium in the absence of lactogenic stimuli.** *Endocrinology* 2002, **143:**3641-3650.

9. Vidarsson H, Mikaelsdottir EK, Rafnar T, Bertwistle D, Ashworth A, Eyfjord JE, Valgeirsdottir S: **BRCA1 and BRCA2 bind Stat5a and suppress its transcriptional activity.** *FEBS Lett* 2002, **532:**247-252.

10. Zhou Y, Jiang H: **Short communication: A milk trait-associated polymorphism in the bovine growth hormone receptor gene does not affect receptor signaling.** *J Dairy Sci* 2006, **89:**1761-1764.

11. Long E, Lazaris-Karatzas A, Karatzas C, Zhao X: **Overexpressing eukaryotic translation initiation factor 4E stimulates bovine mammary epithelial cell proliferation.** *Int J Biochem Cell Biol* 2001, **33:**133-141.

12. Long E, Capuco AV, Zhao X: **Cloning of bovine eukaryotic translation initiation factor 4E (eIF-4E) and its expression in the bovine mammary gland at different physiological stages.** *DNA Seq* 2001, **12:**319-329.

13. Le Provost F, Miyoshi K, Vilotte JL, Bierie B, Robinson GW, Hennighausen L: **SOCS3 promotes apoptosis of mammary differentiated cells.** *Biochem Biophys Res Commun* 2005, **338:**1696-1701.

14. Hovey RC, Harris J, Hadsell DL, Lee AV, Ormandy CJ, Vonderhaar BK: **Local insulin-like growth factor-II mediates prolactin-induced mammary gland development.** *Mol Endocrinol* 2003, **17:**460-471.

15. Lee AV, Zhang P, Ivanova M, Bonnette S, Oesterreich S, Rosen JM, Grimm S, Hovey RC, Vonderhaar BK, Kahn CR, et al: **Developmental and hormonal signals dramatically alter the localization and abundance of insulin receptor substrate proteins in the mammary gland.** *Endocrinology* 2003, **144:**2683-2694.

16. Lindeman GJ, Wittlin S, Lada H, Naylor MJ, Santamaria M, Zhang JG, Starr R, Hilton DJ, Alexander WS, Ormandy CJ, Visvader J: **SOCS1 deficiency results in accelerated mammary gland development and rescues lactation in prolactin receptor-deficient mice.** *Genes Dev* 2001, **15:**1631-1636.

17. Robinson SD, Silberstein GB, Roberts AB, Flanders KC, Daniel CW: **Regulated expression and growth inhibitory effects of transforming growth factor-beta isoforms in mouse mammary gland development.** *Development* 1991, **113:**867-878.

18. Carrascosa JM, Ramos P, Molero JC, Herrera E: **Changes in the kinase activity of the insulin receptor account for an increased insulin sensitivity of mammary gland in late pregnancy.** *Endocrinology* 1998, **139:**520-526.

19. Raught B, Liao WS, Rosen JM: **Developmentally and hormonally regulated CCAAT/enhancer-binding protein isoforms influence beta-casein gene expression.** *Mol Endocrinol* 1995, **9:**1223-1232.

20. Seagroves TN, Krnacik S, Raught B, Gay J, Burgess-Beusse B, Darlington GJ, Rosen JM: **C/EBPbeta, but not C/EBPalpha, is essential for ductal morphogenesis, lobuloalveolar proliferation, and functional differentiation in the mouse mammary gland.** *Genes Dev* 1998, **12:**1917-1928.

21. Wintermantel TM, Bock D, Fleig V, Greiner EF, Schutz G: **The epithelial glucocorticoid receptor is required for the normal timing of cell proliferation during mammary lobuloalveolar development but is dispensable for milk production.** *Mol Endocrinol* 2005, **19:**340-349.
